# Supplementary material for: Does Prior Respiratory Viral Infection Provide Cross-Protection Against Subsequent Respiratory Viral Infections? A Systematic Review and Meta-Analysis
Source: Viruses. 2024 Jun 19;16(6):982. doi: 10.3390/v16060982 (PMC11209343; doi:10.3390/v16060982)
Supplement: Supplementary file 1 [file viruses-16-00982-s001.zip › viruses-3029713-supplementary.pdf]

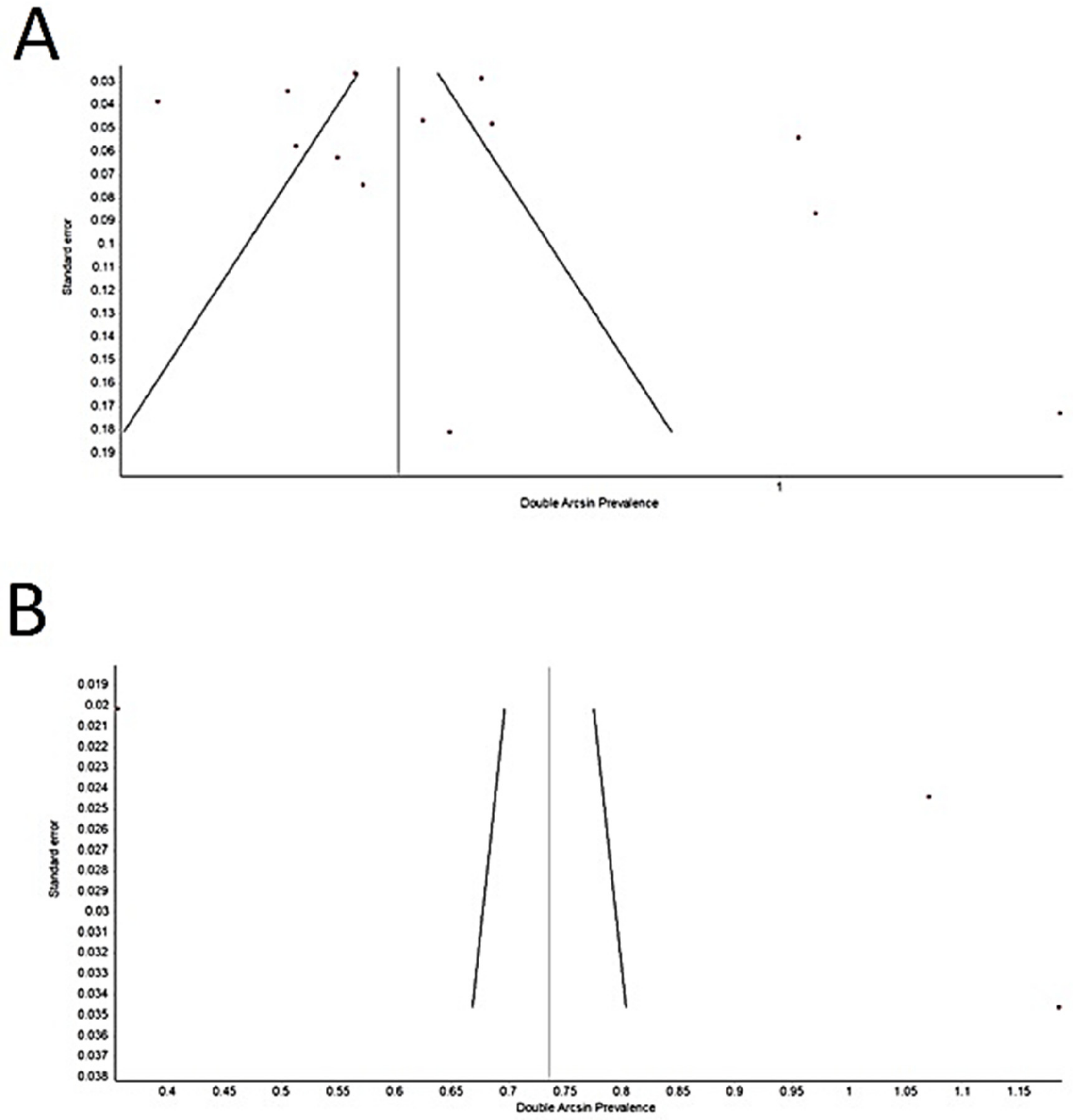

Supplementary Figure S1. (A) Funnel plot for studies showing positive viral swab (any virus) following an initial viral infection (any virus) (B) Funnel plot studies showing a positive viral swab (any virus) following an initial negative swab.

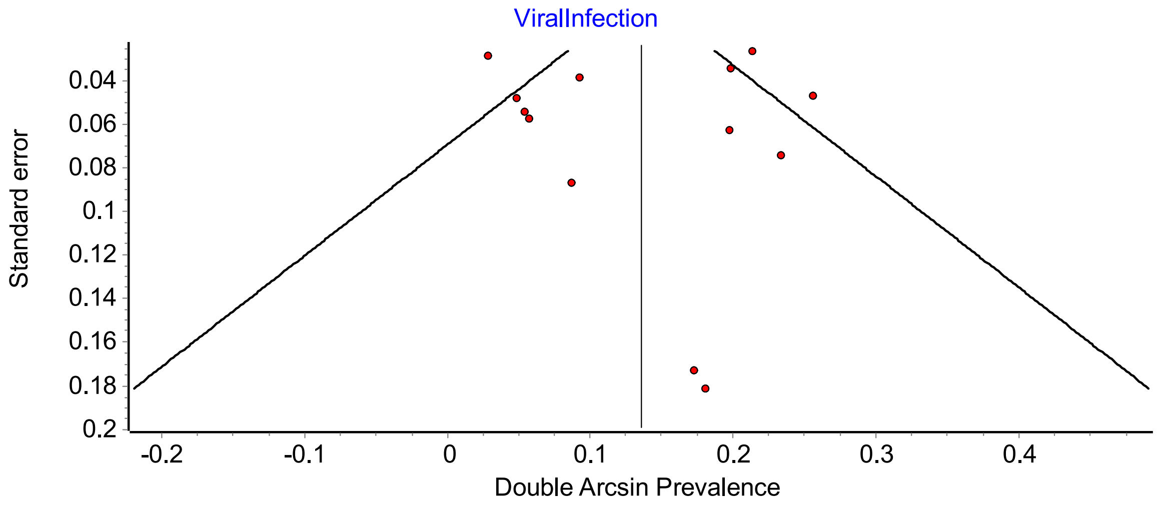

Supplementary Figure S2. Funnel plot for studies showing pooled prevalence of human coronavirus infection following an initial positive viral swab.

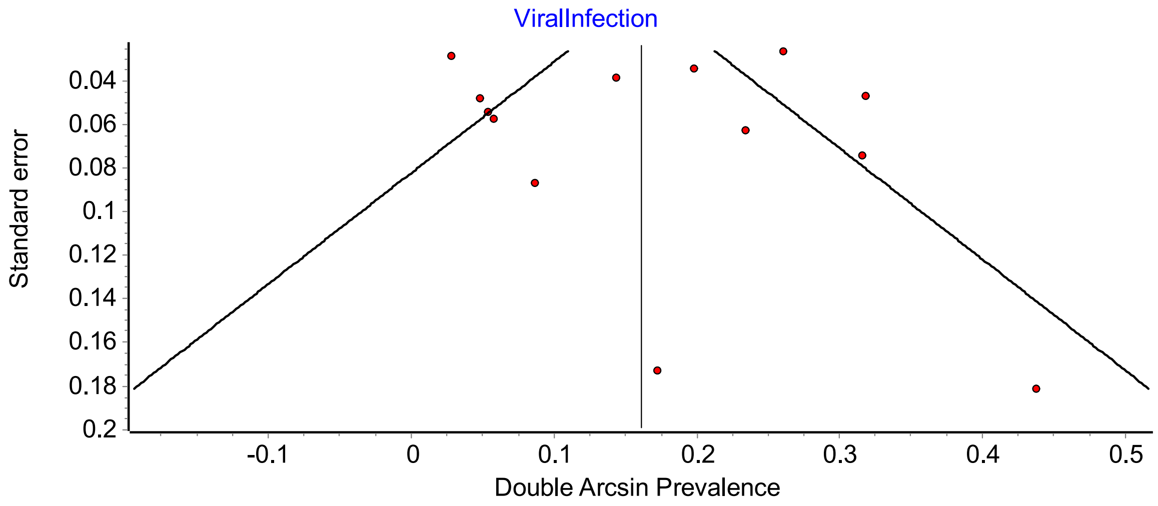

Supplementary Figure S3. Funnel plot for studies on positive enterovirus/rhinovirus infection, following an initial positive viral swab.

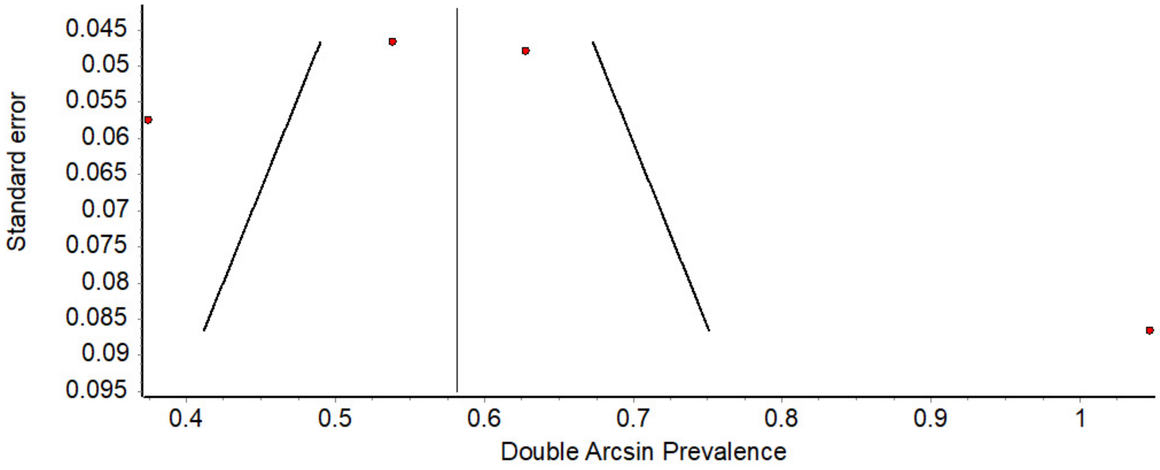

Supplementary Figure S4. Funnel plot for studies examining a positive viral swab following an initial human coronavirus infection.

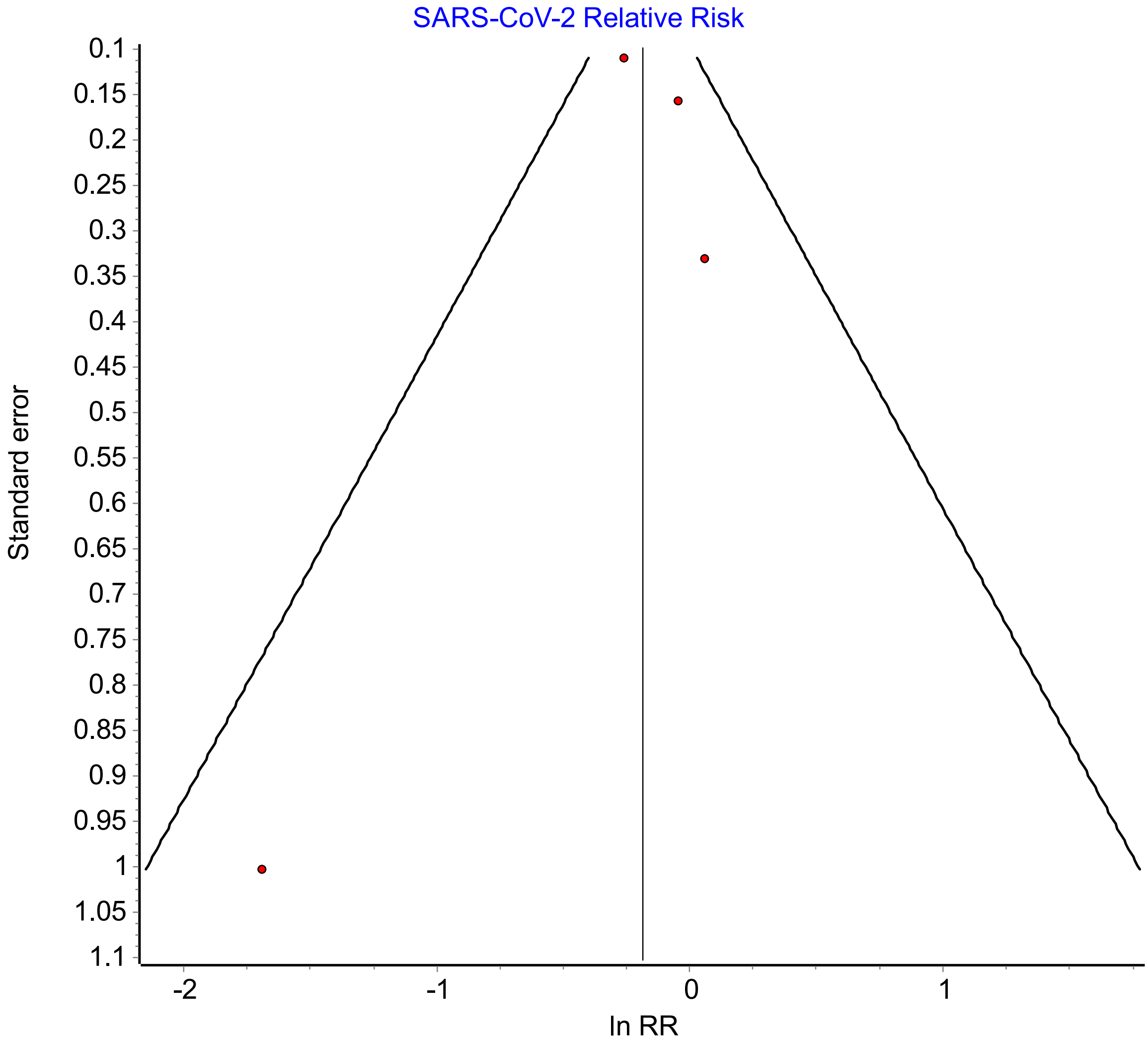

Supplemental Figure S5. Funnel plot of studies examining relative risk of SARS-CoV-2 infection following an initial positive viral swab compared with negative swab.

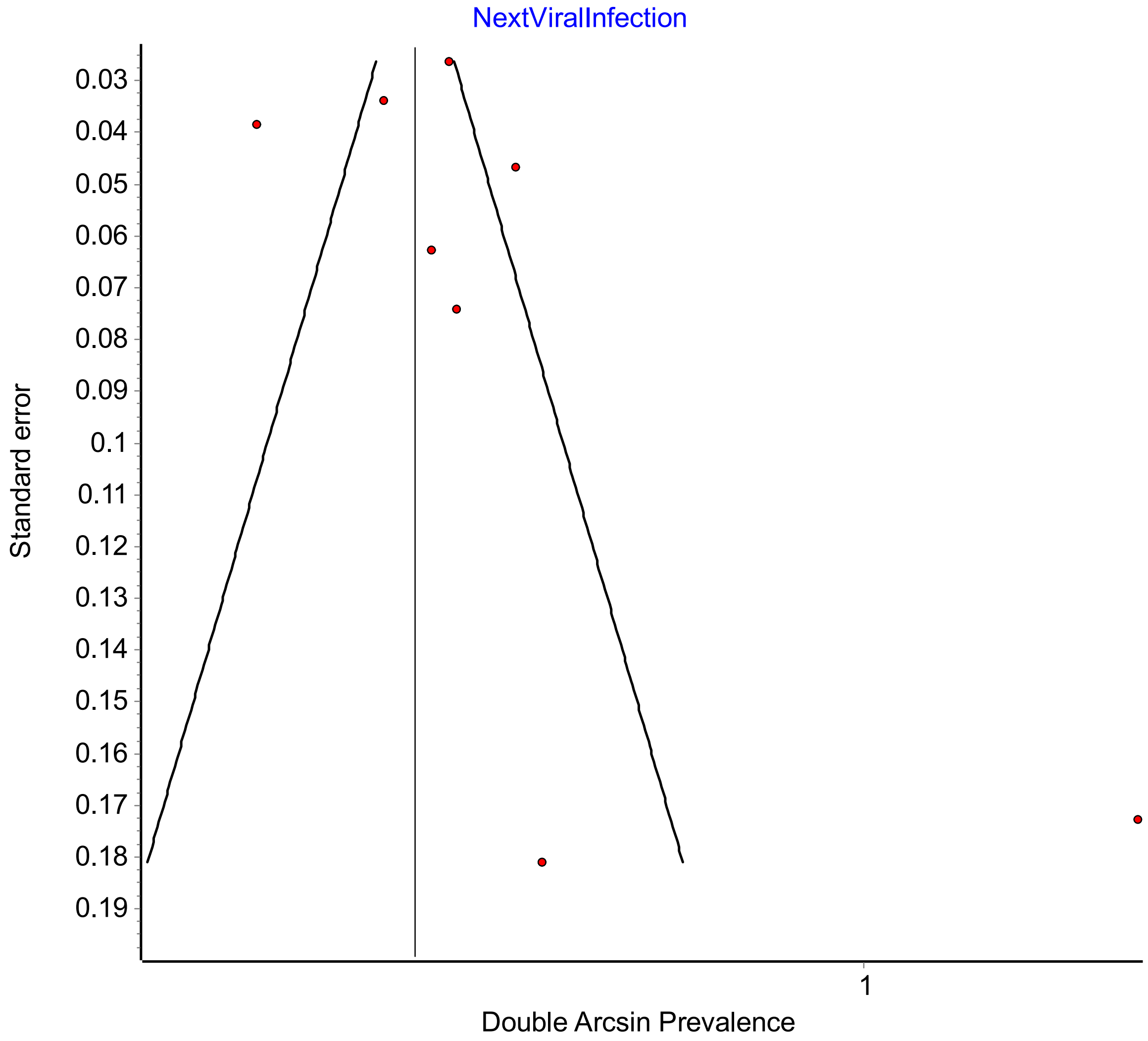

Supplemental Figure S6. Funnel plot of subgroup of studies excluding SARS-CoV-2, examining pooled prevalence of a secondary viral infection following an initial positive swab.

Supplementary Material. List of excluded papers from the review

1. Zanoni, L., et al., *[18F]-FDG PET/CT for suspected lymphoma relapse in a patient with concomitant pneumococcal pneumonia during COVID-19 outbreak: unexpected SARS-Cov-2 co-infection despite double RT-PCR negativity.* European Journal of Nuclear Medicine and Molecular Imaging, 2020. **47**(8): p. 2038-2039.
2. Miller, M.S., et al., *1976 and 2009 H1N1 influenza virus vaccines boost anti-hemagglutinin stalk antibodies in humans.* J Infect Dis, 2013. **207**(1): p. 98-105.
3. Kieffer, A., et al., *2009 A(H1N1) seroconversion rates and risk factors among the general population in Vientiane Capital, Laos.* PLoS One, 2013. **8**(4): p. e61909.
4. Zabaleta, N., et al., *An AAV-based, room-temperature-stable, single-dose COVID-19 vaccine provides durable immunogenicity and protection in non-human primates.* Cell Host and Microbe, 2021. **29**(9): p. 1437-1453.e8.
5. Vu, L.D., et al., *Absence of antibody responses to SARS-CoV-2 N protein in COVID-19 vaccine breakthrough cases.* Experimental Biology and Medicine, 2022. **247**(21): p. 1923-1936.
6. McVernon, J., et al., *Absence of cross-reactive antibodies to influenza A (H1N1) 2009 before and after vaccination with 2009 Southern Hemisphere seasonal trivalent influenza vaccine in children aged 6 months-9 years: a prospective study.* Influenza Other Respir Viruses, 2011. **5**(1): p. 7-11.

7. van Woensel, J.B., et al., *Absence of human metapneumovirus co-infection in cases of severe respiratory syncytial virus infection*. Pediatr Pulmonol, 2006. **41**(9): p. 872-4.
8. Chau, N.V.V., et al., *Absence of SARS-CoV-2 antibodies in pre-pandemic plasma from children and adults in Vietnam*. International Journal of Infectious Diseases, 2021. **111**: p. 127-129.
9. *Abstracts of the papers presented in the international conference of Indian virological society, ''Global viral epidemics: a challenging threat", during 12–14 November, 2018, at PGIMER, Chandigarh, India*. VirusDisease, 2019. **30**(1): p. 112-169.
10. Wang, R., et al., *Acceptance of seasonal influenza vaccination and associated factors among pregnant women in the context of COVID-19 pandemic in China: a multi-center cross-sectional study based on health belief model*. BMC Pregnancy and Childbirth, 2021. **21**(1).
11. Panchali, M.J.L., et al., *Accuracy of Real-Time Polymerase Chain Reaction in COVID-19 Patients*. Microbiology Spectrum, 2022. **10**(1).
12. Mação, P., et al., *[Acute bronchiolitis: a prospective study]*. Acta Med Port, 2011. **24 Suppl 2**: p. 407-12.
13. Wang, L., et al., *Acute graft-versus-host disease after liver transplantation in a close contact with COVID-19: A case report*. Transplant Immunology, 2021. **68**.
14. D'Abramo, A., et al., *Acute respiratory distress syndrome due to SARS-CoV-2 and Influenza A co-infection in an Italian patient: Mini-review of the literature*. International Journal of Infectious Diseases, 2020. **97**: p. 236-239.
15. Kitchin, D., et al., *Ad26.COV2.S breakthrough infections induce high titers of neutralizing antibodies against Omicron and other SARS-CoV-2 variants of concern*. Cell Reports Medicine, 2022. **3**(3).
16. Sharov, K.S., *Adaptation of a Russian population to SARS-CoV-2: Asymptomatic course, comorbidities, mortality, and other respiratory viruses - A reply to Fear versus Data*. Int J Antimicrob Agents, 2020. **56**(4): p. 106093.
17. Tan, H.X., et al., *Adaptive immunity to human coronaviruses is widespread but low in magnitude*. Clinical and Translational Immunology, 2021. **10**(3).
18. Baker, P.J., *Advantages of an Oral Vaccine to Control the COVID-19 Pandemic*. American Journal of Medicine, 2022. **135**(2): p. 133-134.
19. Rutkowski, K., et al., *Adverse reactions to COVID-19 vaccines: A practical approach*. Clinical and Experimental Allergy, 2021. **51**(6): p. 770-777.
20. Salvador García, C., et al., *[Aetiology of bronchiolitis in hospitalised children in South-East Spain]*. An Pediatr (Barc), 2012. **77**(6): p. 386-90.
21. Mermond, S., et al., *Aetiology of community-acquired pneumonia in hospitalized adult patients in New Caledonia*. Trop Med Int Health, 2010. **15**(12): p. 1517-24.
22. Boum, Y., L.M. Bebell, and A.C.Z.K. Bisseck, *Africa needs local solutions to face the COVID-19 pandemic*. The Lancet, 2021. **397**(10281): p. 1238-1240.
23. Tanunliong, G., et al., *Age-Associated Seroprevalence of Coronavirus Antibodies: Population-Based Serosurveys in 2013 and 2020, British Columbia, Canada*. Frontiers in Immunology, 2022. **13**.
24. Smith, F.S., et al., *Age-related development of human memory T-helper and B-cell responses toward parainfluenza virus type-1*. Virology, 1994. **205**(2): p. 453-61.
25. Goplen, N.P., I.S. Cheon, and J. Sun, *Age-Related Dynamics of Lung-Resident Memory CD8+ T Cells in the Age of COVID-19*. Frontiers in Immunology, 2021. **12**.
26. Ranjeva, S., et al., *Age-specific differences in the dynamics of protective immunity to influenza*. Nat Commun, 2019. **10**(1): p. 1660.
27. Tajbakhsh, A., et al., *Age-Specific Differences in the Severity of COVID-19 Between Children and Adults: Reality and Reasons*, in *Advances in Experimental Medicine and Biology*. 2021. p. 63-78.
28. Diniz, M.O., et al., *Airway-resident T cells from unexposed individuals cross-recognize SARS-CoV-2*. Nature Immunology, 2022.
29. Har-Noy, M. and R. Or, *Allo-priming as a universal anti-viral vaccine: Protecting elderly from current COVID-19 and any future unknown viral outbreak*. Journal of Translational Medicine, 2020. **18**(1).
30. Cantoni, D., et al., *Analysis of Antibody Neutralisation Activity against SARS-CoV-2 Variants and Seasonal Human Coronaviruses NL63, HKU1, and 229E Induced by Three Different COVID-19 Vaccine Platforms*. Vaccines (Basel), 2022. **11**(1).
31. Çağlayan, D., et al., *An analysis of antibody response following the second dose of CoronaVac and humoral response after booster dose with BNT162b2 or CoronaVac among healthcare workers in Turkey*. Journal of Medical Virology, 2022. **94**(5): p. 2212-2221.
32. Hirotsu, Y., et al., *Analysis of Covid-19 and non-Covid-19 viruses, including influenza viruses, to determine the influence of intensive preventive measures in Japan*. J Clin Virol, 2020. **129**: p. 104543.
33. Wen, C., et al., *[Analysis of epidemiological characteristics of human coronavirus in hospitalized children with respiratory tract infection in Hebei region]*. Zhonghua Yu Fang Yi Xue Za Zhi, 2021. **55**(11): p. 1321-1327.
34. Lu, D.E., et al., *Analysis of Fungal and Bacterial Co-Infections in Mortality Cases among Hospitalized Patients with COVID-19 in Taipei, Taiwan*. Journal of Fungi, 2022. **8**(1).
35. Henss, L., et al., *Analysis of Humoral Immune Responses in Patients With Severe Acute Respiratory Syndrome Coronavirus 2 Infection*. J Infect Dis, 2021. **223**(1): p. 56-61.

36. Meinberger, D., et al., *Analysis of IgM, IgA, and IgG isotype antibodies Directed against SARS-CoV-2 spike glycoprotein and ORF8 in the course of COVID-19.* Sci Rep, 2021. **11**(1): p. 8920.
37. Chan, C.W., et al., *Analytical and Clinical Evaluation of the Automated Elecsys Anti-SARS-CoV-2 Antibody Assay on the Roche cobas e602 Analyzer.* American Journal of Clinical Pathology, 2020. **154**(5): p. 620-626.
38. Chan, C.W., et al., *Analytical and Clinical Evaluation of the Semiquantitative Elecsys Anti-SARS-CoV-2 Spike Protein Receptor Binding Domain Antibody Assay on the Roche cobas e602 Analyzer.* American journal of clinical pathology, 2022. **157**(1): p. 109-118.
39. Tré-Hardy, M., et al., *Analytical and clinical validation of an ELISA for specific SARS-CoV-2 IgG, IgA, and IgM antibodies.* J Med Virol, 2021. **93**(2): p. 803-811.
40. Andreano, E., et al., *Anatomy of Omicron BA.1 and BA.2 neutralizing antibodies in COVID-19 mRNA vaccinees.* Nature Communications, 2022. **13**(1).
41. Jiang, Y., et al., *Angiotensin II receptor I auto-antibodies following SARS-CoV-2 infection.* PLoS ONE, 2021. **16**(11 November).
42. Saavedra, J.M., *Angiotensin receptor blockers and COVID-19.* Pharmacological Research, 2020. **156**.
43. Mavrikou, S., et al., *Angiotensin-converting enzyme 2 (Ace2) as a novel biorecognition element in a cell-based biosensor for the ultra-rapid, ultra-sensitive detection of the sars-cov-2 s1 spike protein antigen.* Chemosensors, 2021. **9**(12).
44. Frasca, F., et al., *Anti-IFN-α/-ω neutralizing antibodies from COVID-19 patients correlate with downregulation of IFN response and laboratory biomarkers of disease severity.* European Journal of Immunology, 2022. **52**(7): p. 1120-1128.
45. Borghi, M.O., et al., *Anti-Phospholipid Antibodies and Coronavirus Disease 2019: Vaccination Does Not Trigger Early Autoantibody Production in Healthcare Workers.* Frontiers in Immunology, 2022. **13**.
46. Dalakas, M.C., K. Bitzogli, and H. Alexopoulos, *Anti-SARS-CoV-2 Antibodies Within IVIg Preparations: Cross-Reactivities With Seasonal Coronaviruses, Natural Autoimmunity, and Therapeutic Implications.* Frontiers in Immunology, 2021. **12**.
47. Andrew Seaton, R., et al., *Antibiotic prescribing for respiratory tract infection in patients with suspected and proven COVID-19: Results from an antibiotic point prevalence survey in Scottish hospitals.* JAC-Antimicrobial Resistance, 2021. **3**(2).
48. Langford, B.J., et al., *Antibiotic prescribing in patients with COVID-19: rapid review and meta-analysis.* Clinical Microbiology and Infection, 2021. **27**(4): p. 520-531.
49. Silva, I.C., et al., *Antibiotic prescription in hospitalized patients with covid-19 in a private hospital in caracas: Retrospective study.* Archivos Venezolanos de Farmacologia y Terapeutica, 2021. **40**(4): p. 424-431.
50. Van Laethem, J., et al., *Antibiotic prescriptions in the context of suspected bacterial respiratory tract superinfections in the COVID-19 era: a retrospective quantitative analysis of antibiotic consumption and identification of antibiotic prescription drivers.* Intern Emerg Med, 2022. **17**(1): p. 141-151.
51. Kolář, M., et al., *Antibiotic treatment issues in patients with COVID-19.* Vnitrni Lekarstvi, 2021. **67**(8): p. 448-454.
52. Evans, T.J., et al., *Antibiotic usage and stewardship in patients with COVID-19: too much antibiotic in uncharted waters?* Journal of Infection Prevention, 2021. **22**(3): p. 119-125.
53. Salehi, M., et al., *Antibiotic use during the first 6 months of COVID-19 pandemic in Iran: A large-scale multi-centre study.* Journal of Clinical Pharmacy and Therapeutics, 2022.
54. Er, J.C., B. Lium, and T. Framstad, *Antibodies of influenza A(H1N1)pdm09 virus in pigs' sera cross-react with other influenza A virus subtypes. A retrospective epidemiological interpretation of Norway's serosurveillance data from 2009-2017.* Epidemiol Infect, 2020. **148**: p. e73.
55. Peddireddy, S.P., et al., *Antibodies targeting conserved non-canonical antigens and endemic coronaviruses associate with favorable outcomes in severe COVID-19.* Cell Reports, 2022. **39**(13).
56. Röltgen, K. and S.D. Boyd, *Antibody and B cell responses to SARS-CoV-2 infection and vaccination.* Cell Host and Microbe, 2021. **29**(7): p. 1063-1075.
57. Garanina, E., et al., *Antibody and T Cell Immune Responses to SARS-CoV-2 Peptides in COVID-19 Convalescent Patients.* Frontiers in Microbiology, 2022. **13**.
58. Rouhani, S.J., et al., *Antibody and T cell responses to COVID-19 vaccination in patients receiving anticancer therapies.* Journal for ImmunoTherapy of Cancer, 2022. **10**(6).
59. Ge, J., et al., *Antibody neutralization of SARS-CoV-2 through ACE2 receptor mimicry.* Nature Communications, 2021. **12**(1).
60. Bagri, A., et al., *Antibody profiles in COVID-19 convalescent plasma prepared with amotosalen/UVA pathogen reduction treatment.* Transfusion, 2022. **62**(3): p. 570-583.
61. Ansaldi, F., et al., *Antibody response against heterogeneous circulating influenza virus strains elicited by MF59- and non-adjuvanted vaccines during seasons with good or partial matching between vaccine strain and clinical isolates.* Vaccine, 2010. **28**(25): p. 4123-9.
62. Ruetalo, N., et al., *Antibody response against sars-cov-2 and seasonal coronaviruses in nonhospitalized covid-19 patients.* mSphere, 2021. **6**(1).

63. Liu, C., et al., *The antibody response to SARS-CoV-2 Beta underscores the antigenic distance to other variants.* Cell Host and Microbe, 2022. **30**(1): p. 53-68.e12.
64. Hueston, L., et al., *The antibody response to SARS-CoV-2 infection.* Open Forum Infectious Diseases, 2020. **7**(9).
65. Post, N., et al., *Antibody response to SARS-CoV-2 infection in humans: A systematic review.* PLoS ONE, 2020. **15**(12).
66. Morgenlander, W., et al., *Antibody responses to endemic coronaviruses modulate COVID-19 convalescent plasma functionality*. Journal of Clinical Investigation, 2021. **131**(7).
67. Ramos, I., et al., *Antibody Responses to SARS-CoV-2 Following an Outbreak Among Marine Recruits With Asymptomatic or Mild Infection.* Front Immunol, 2021. **12**: p. 681586.
68. Long, Q.X., et al., *Antibody responses to SARS-CoV-2 in patients with COVID-19.* Nature Medicine, 2020. **26**(6): p. 845-848.
69. Baumgarth, N., et al., *Antibody responses to SARS-CoV-2: Let's stick to known knowns.* Journal of Immunology, 2020. **205**(9): p. 2342-2350.
70. Agency, U.H.S. *Antibody testing for SARS-CoV-2: key information*. Testing for coronavirus (COVID-19) 2022 4 April 2022; Available from: https://www.gov.uk/government/publications/antibody-testing-for-sars-cov-2-key-information/antibody-testing-for-sars-cov-2-information-for-general-practitioners.
71. Shukla, A.K. and S. Misra, *Antibody-dependent enhancement of virus infection and disease: implications in COVID-19.* Journal of Basic and Clinical Physiology and Pharmacology, 2022. **33**(1): p. 13-16.
72. Wrobel, A.G., et al., *Antibody-mediated disruption of the SARS-CoV-2 spike glycoprotein.* Nature Communications, 2020. **11**(1).
73. Errico, J.M., L.J. Adams, and D.H. Fremont, *Antibody-mediated immunity to SARS-CoV-2 spike*, in *Advances in Immunology*. 2022.
74. Leroux-Roels, I., et al., *Antigen sparing and cross-reactive immunity with an adjuvanted rH5N1 prototype pandemic influenza vaccine: a randomised controlled trial.* Lancet, 2007. **370**(9587): p. 580-9.
75. Zhang, C., et al., *Antigenic Evolution on a Global Scale Reveals the Potential Natural Selection of Severe Acute Respiratory Syndrome-Coronavirus 2 by Pre-existing Cross-Reactive T-Cell Immunity.* Frontiers in Microbiology, 2021. **12**.
76. Fierens, J., et al., *Antimicrobial prescription in severe COVID-19 and CAP: a matched case-control study.* Acta Clin Belg, 2022. **77**(5): p. 837-844.
77. Kariyawasam, R.M., et al., *Antimicrobial resistance (AMR) in COVID-19 patients: a systematic review and meta-analysis (November 2019–June 2021).* Antimicrobial Resistance and Infection Control, 2022. **11**(1).
78. Schouten, J., et al., *Antimicrobial stewardship in the ICU in COVID-19 times: the known unknowns.* International Journal of Antimicrobial Agents, 2021. **58**(4).
79. Cong, W., et al., *Antimicrobial use in covid-19 patients in the first phase of the sars-cov-2 pandemic: A scoping review.* Antibiotics, 2021. **10**(6).
80. Rand, U., et al., *Antiviral activity of influenza a virus defective interfering particles against sars-cov-2 replication in vitro through stimulation of innate immunity.* Cells, 2021. **10**(7).
81. Lai, C.C. and W.L. Yu, *Appropriate use of antimicrobial therapy for COVID-19 co-infection.* Immunotherapy, 2021. **13**(13): p. 1067-1070.
82. Male, V., *Are COVID-19 vaccines safe in pregnancy?* Nature Reviews Immunology, 2021. **21**(4): p. 200-201.
83. Yin, J.K., et al., *Assessing seasonal vaccine-related cross-protection from 2009 pandemic H1N1 influenza through teacher absenteeism.* Aust N Z J Public Health, 2011. **35**(4): p. 393-4.
84. Kilonzo, C.M., et al., *Assessing the impact of non-pharmaceutical interventions (NPIs) and BCG vaccine cross-protection in the transmission dynamics of SARS-CoV-2 in eastern Africa.* BMC research notes, 2022. **15**(1): p. 283.
85. Santoso, M.S., et al., *Assessment of dengue and COVID-19 antibody rapid diagnostic tests cross-reactivity in Indonesia.* Virology Journal, 2021. **18**(1).
86. Arianezhad, A., B. Azizolahi, and R. Ghaffaripour, *The Association Between COVID-19 and Invasive Pulmonary Aspergillosis: A Challenge for Health Care System.* Acta Medica Iranica, 2022. **60**(6): p. 322-328.
87. Kasperkiewicz, M., *Association between COVID-19 vaccination and autoimmune bullous diseases: a random coincidence or rare event.* Journal of the European Academy of Dermatology and Venereology, 2022. **36**(9): p. e665-e666.
88. Su, W., et al., *The Association Between Previous Influenza Vaccination and COVID-19 Infection Risk and Severity: A Systematic Review and Meta-analysis.* Am J Prev Med, 2022. **63**(1): p. 121-130.
89. Hosseini, S.S., et al., *Association between respiratory viruses and exacerbation of COPD: a case-control study.* Infect Dis (Lond), 2015. **47**(8): p. 523-9.
90. Kasperkiewicz, M. and D.T. Woodley, *Association between vaccination and immunobullous disorders: a brief, updated systematic review with focus on COVID-19.* Journal of the European Academy of Dermatology and Venereology, 2022. **36**(7): p. e498-e500.
91. Zhu, Y., et al., *Association of neutralizing breadth against SARS-CoV-2 with inoculation orders of heterologous prime-boost vaccines.* Med, 2022. **3**(8): p. 568-578.e3.

92. Chibwana, M.G., et al., *AstraZeneca COVID-19 vaccine induces robust broadly cross-reactive antibody responses in Malawian adults previously infected with SARS-CoV-2*. BMC Med, 2022. **20**(1): p. 128.
93. Chaudhry, R., et al., *Atypical bacterial co-infections among patients with COVID-19: A study from India*. J Med Virol, 2022. **94**(1): p. 303-309.
94. Ou, X., et al., *Author Correction: Characterization of spike glycoprotein of SARS-CoV-2 on virus entry and its immune cross-reactivity with SARS-CoV (Nature Communications, (2020), 11, 1, (1620), 10.1038/s41467-020-15562-9)*. Nature Communications, 2021. **12**(1).
95. Tang, J., et al., *Author Correction: Cross-reactive immunity against the SARS-CoV-2 Omicron variant is low in pediatric patients with prior COVID-19 or MIS-C (Nature Communications, (2022), 13, 1, (2979), 10.1038/s41467-022-30649-1)*. Nature Communications, 2022. **13**(1).
96. Hojjat Jodaylami, M., et al., *Author Correction: Cross-reactivity of antibodies from non-hospitalized COVID-19 positive individuals against the native, B.1.351, B.1.617.2, and P.1 SARS-CoV-2 spike proteins (Scientific Reports, (2021), 11, 1, (21601), 10.1038/s41598-021-00844-z)*. Scientific Reports, 2021. **11**(1).
97. Taeschler, P., et al., *Autoantibodies in COVID-19 correlate with antiviral humoral responses and distinct immune signatures*. Allergy: European Journal of Allergy and Clinical Immunology, 2022. **77**(8): p. 2415-2430.
98. Matyushkina, D., et al., *Autoimmune Effect of Antibodies against the SARS-CoV-2 Nucleoprotein*. Viruses, 2022. **14**(6).
99. Garrido, I., et al., *Autoimmune hepatitis after COVID-19 vaccine – more than a coincidence*. Journal of Autoimmunity, 2021. **125**.
100. Peiris, M., *Avian influenza in humans: Impact, surveillance and research*. Influenza and other Respiratory Viruses, 2010. **4**: p. 5.
101. Meijer, E.F.J., et al., *Azole-resistant COVID-19-associated pulmonary aspergillosis in an immunocompetent host: A case report*. Journal of Fungi, 2020. **6**(2): p. 1-8.
102. Cao, Y., et al., *BA.2.12.1, BA.4 and BA.5 escape antibodies elicited by Omicron infection*. Nature, 2022. **608**(7923): p. 593-602.
103. Silva Júnior, J.V.J., et al., *Background immunity: How important is it for SARS-CoV-2?* Journal of Medical Virology, 2021. **93**(3): p. 1253-1254.
104. Ceccarelli, M., et al., *Bacterial and Fungal Co-Infections and Superinfections in a Cohort of COVID-19 Patients: Real-Life Data from an Italian Third Level Hospital*. Infectious Disease Reports, 2022. **14**(3): p. 372-382.
105. Hughes, S., et al., *Bacterial and fungal coinfection among hospitalized patients with COVID-19: a retrospective cohort study in a UK secondary-care setting*. Clin Microbiol Infect, 2020. **26**(10): p. 1395-1399.
106. Alnimr, A.M., et al., *Bacterial and Fungal Coinfection in Critically Ill COVID-19 Cases and Predictive Role of Procalcitonin During the First Wave at an Academic Health Center*. J Epidemiol Glob Health, 2022. **12**(2): p. 188-195.
107. Shah, N.S., et al., *Bacterial and viral co-infections complicating severe influenza: Incidence and impact among 507 U.S. patients, 2013-14*. J Clin Virol, 2016. **80**: p. 12-9.
108. Langford, B.J., et al., *Bacterial co-infection and secondary infection in patients with COVID-19: a living rapid review and meta-analysis*. Clinical Microbiology and Infection, 2020. **26**(12): p. 1622-1629.
109. Moreno-García, E., et al., *Bacterial co-infection at hospital admission in patients with COVID-19: Bacterial co-infections in COVID-19*. International Journal of Infectious Diseases, 2022. **118**: p. 197-202.
110. Santos, A.P., et al., *Bacterial Co-Infection in Patients with COVID-19 Hospitalized (ICU and Not ICU): Review and Meta-Analysis*. Antibiotics, 2022. **11**(7).
111. Cheng, L.S.K., et al., *Bacterial co-infections and antibiotic prescribing practice in adults with COVID-19: experience from a single hospital cluster*. Therapeutic Advances in Infectious Disease, 2020. **7**.
112. Ruiz-Bastián, M., et al., *Bacterial co-infections in COVID-19 pneumonia in a tertiary care hospital: Surfing the first wave*. Diagn Microbiol Infect Dis, 2021. **101**(3): p. 115477.
113. De Santis, V., et al., *Bacterial infections in critically ill patients with SARS-2-COVID-19 infection: results of a prospective observational multicenter study*. Infection, 2022. **50**(1): p. 139-148.
114. del Fresno, C., et al., *The Bacterial Mucosal Immunotherapy MV130 Protects Against SARS-CoV-2 Infection and Improves COVID-19 Vaccines Immunogenicity*. Frontiers in Immunology, 2021. **12**.
115. Buehler, P.K., et al., *Bacterial pulmonary superinfections are associated with longer duration of ventilation in critically ill COVID-19 patients*. Cell Rep Med, 2021. **2**(4): p. 100229.
116. Kelleher, P., *The battle of testing in COVID-19: the secrets of victory against the virus*. Cardiovascular Research, 2021. **117**(8): p. E101-E103.
117. Junqueira-Kipnis, A.P., et al., *BCG revaccination of health workers in Brazil to improve innate immune responses against COVID-19: A structured summary of a study protocol for a randomised controlled trial*. Trials, 2020. **21**(1).
118. Bates, M.N., et al., *BCG vaccination at birth and COVID-19: a case-control study among U.S. military Veterans*. Hum Vaccin Immunother, 2022. **18**(1): p. 1981084.
119. Pandita, A., et al., *BCG Vaccination Program Mitigates COVID19 Related Mortality: A Reality Check*. Current pharmaceutical biotechnology, 2021. **22**(12): p. 1574-1583.

120. Gong, W., et al., *BCG Vaccination: A potential tool against COVID-19 and COVID-19-like Black Swan incidents*. International Immunopharmacology, 2022. **108**.
121. Gonzalez-Perez, M., et al., *The BCG Vaccine for COVID-19: First Verdict and Future Directions*. Frontiers in Immunology, 2021. **12**.
122. Koneru, G., et al., *BCG vaccine-induced trained immunity and COVID-19: Protective or bystander?* Infection and Drug Resistance, 2021. **14**: p. 1169-1184.
123. Malik, Y.S., et al., *BCG vaccine: a hope to control COVID-19 pandemic amid crisis*. Human Vaccines and Immunotherapeutics, 2020. **16**(12): p. 2954-2962.
124. Hartley, G.E., M.C. van Zelm, and M.J. Robinson, *The benefit of boosters: diversity and inclusion in the COVID-19 memory response*. Immunology and Cell Biology, 2022. **100**(1): p. 15-17.
125. Sidiq, Z., et al., *Benefits and limitations of serological assays in COVID-19 infection*. Indian Journal of Tuberculosis, 2020. **67**(4): p. S163-S166.
126. Callaway, E., *Beyond Omicron: what's next for COVID's viral evolution*. Nature, 2021. **600**(7888): p. 204-207.
127. Si, Y., et al., *Beyond Vaccination Rates: A Synthetic Random Proxy Metric of Total SARS-CoV-2 Immunity Seroprevalence in the Community*. Epidemiology, 2022. **33**(4): p. 457-464.
128. Cabrera Muras, A., et al., *Bilateral facial nerve palsy associated with COVID-19 and Epstein–Barr virus co-infection*. European Journal of Neurology, 2021. **28**(1): p. 358-360.
129. Elkoshi, Z., *The Binary Model of Chronic Diseases Applied to COVID-19*. Frontiers in Immunology, 2021. **12**.
130. Mourya, D., et al., *Biorisk assessment for infrastructure & biosafety requirements for the laboratories providing coronavirus SARS-CoV-2/(COVID-19) diagnosis*. Indian Journal of Medical Research, 2020. **151**(2): p. 172-176.
131. Mondal, A., M. Gireeshwar, and L. Govindaraj, *Black Fungus Mutilating COVID-19 Pandemic in India: Facts and Immunological Perspectives*. European Journal of Biology, 2022. **81**(1): p. 96-106.
132. Ventoulis, I., et al., *Bloodstream infection by saccharomyces cerevisiae in two covid-19 patients after receiving supplementation of saccharomyces in the icu*. Journal of Fungi, 2020. **6**(3): p. 1-7.
133. Chang, X., et al., *BNT162b2 mRNA COVID-19 vaccine induces antibodies of broader cross-reactivity than natural infection, but recognition of mutant viruses is up to 10-fold reduced*. Allergy: European Journal of Allergy and Clinical Immunology, 2021. **76**(9): p. 2895-2998.
134. Vizcarra, P., et al., *BNT162b2 mRNA COVID-19 vaccine Reactogenicity: The key role of immunity*. Vaccine, 2021. **39**(51): p. 7367-7374.
135. Jahrsdörfer, B., et al., *BNT162b2 Vaccination Elicits Strong Serological Immune Responses Against SARS-CoV-2 Including Variants of Concern in Elderly Convalescents*. Front Immunol, 2021. **12**: p. 743422.
136. Lavinder, J.J. and G.C. Ippolito, *Boosted immunity to the common cold might protect children from COVID-19*. Nature Immunology, 2022. **23**(1): p. 8-10.
137. *Booster doses of covid-19 vaccines*. Medical Letter on Drugs and Therapeutics, 2021. **63**(1638): p. 186-188.
138. Crowley, A.R., et al., *Boosting of cross-reactive antibodies to endemic coronaviruses by SARS-CoV-2 infection but not vaccination with stabilized spike*. Elife, 2022. **11**.
139. Li, Y., et al., *Both simulation and sequencing data reveal coinfections with multiple SARS-CoV-2 variants in the COVID-19 pandemic*. Computational and Structural Biotechnology Journal, 2022. **20**: p. 1389-1401.
140. Videla, C.G., et al., *Brain death due to neuroaspergillosis in a patient with COVID-19*. Revista Espanola de Anestesiologia y Reanimacion, 2022.
141. Bardanzellu, F., M. Puddu, and V. Fanos, *Breast milk and covid-19: From conventional data to "omics" technologies to investigate changes occurring in sars-cov-2 positive mothers*. International Journal of Environmental Research and Public Health, 2021. **18**(11).
142. Bejarano, D.A. and A. Schlitzer, *Breathing more breadth into COVID-19 T cell responses*. Med, 2021. **2**(9): p. 999-1001.
143. Kaku, C.I., et al., *Broad anti–SARS-CoV-2 antibody immunity induced by heterologous ChAdOx1/mRNA-1273 vaccination*. Science, 2022. **375**(6584): p. 1041-1047.
144. Pinto, D., et al., *Broad betacoronavirus neutralization by a stem helix–specific human antibody*. Science, 2021. **373**(6559): p. 1109-1116.
145. Wang, J., et al., *Broad Cross-Reactive IgA and IgG against Human Coronaviruses in Milk Induced by COVID-19 Vaccination and Infection*. Vaccines, 2022. **10**(6).
146. Jette, C.A., et al., *Broad cross-reactivity across sarbecoviruses exhibited by a subset of COVID-19 donor-derived neutralizing antibodies*. Cell Reports, 2021. **36**(13).
147. Dubé, C., et al., *Broad neutralization against SARS-CoV-2 variants induced by ancestral and B.1.351 AS03-Adjuvanted recombinant Plant-Derived Virus-Like particle vaccines*. Vaccine, 2022. **40**(30): p. 4017-4025.
148. Wec, A.Z., et al., *Broad neutralization of SARS-related viruses by human monoclonal antibodies*. Science, 2020. **369**(6504): p. 731-736.
149. Marshall, N.C., et al., *Broad respiratory testing to identify SARS-CoV-2 viral co-circulation and inform diagnostic stewardship in the COVID-19 pandemic*. Virol J, 2021. **18**(1): p. 93.
150. Wang, J., et al., *Broadly Reactive IgG Responses to Heterologous H5 Prime-Boost Influenza Vaccination Are Shaped by Antigenic Relatedness to Priming Strains*. mBio, 2021. **12**(4): p. e0044921.

151. Becerra-Artiles, A., et al., *Broadly recognized, cross-reactive SARS-CoV-2 CD4 T cell epitopes are highly conserved across human coronaviruses and presented by common HLA alleles.* Cell Reports, 2022. **39**(11).
152. Shrestha, L.B., N. Tedla, and R.A. Bull, *Broadly-Neutralizing Antibodies Against Emerging SARS-CoV-2 Variants.* Frontiers in Immunology, 2021. **12**.
153. Cangiano, G., et al., *Bronchiolitis: Analysis of 10 consecutive epidemic seasons.* Pediatr Pulmonol, 2016. **51**(12): p. 1330-1335.
154. Cornelissen, C.G., et al., *Broncho-alveolar lavage in patients with acute respiratory distress syndrome due to COVID-19.* Intern Med J, 2021. **51**(6): p. 965-967.
155. Iyer, A., J. Shah, and R. Shah, *The burden and characteristics of HIV-infected COVID-19 patients at a tertiary care hospital in sub-Saharan Africa-A retrospective cohort study.* PLoS One, 2022. **17**(8): p. e0273859.
156. Tramuto, F., et al., *Burden and viral aetiology of influenza-like illness and acute respiratory infection in intensive care units.* Microbes Infect, 2016. **18**(4): p. 270-6.
157. Hupert, N., D. Marín-Hernández, and D.F. Nixon, *Can existing unrelated vaccines boost a COVID-19 vaccine prime?* EClinicalMedicine, 2021. **32**.
158. Eldanasory, O.A., A.A. Rabaan, and J.A. Al-Tawfiq, *Can influenza vaccine modify COVID-19 clinical course?* Travel Medicine and Infectious Disease, 2020. **37**.
159. Brikman, S., et al., *Candida bloodstream infection, a dire complication in hospitalized COVID-19 patients: Three cases from a single center in Northern Israel.* Israel Medical Association Journal, 2021. **23**(10): p. 615-617.
160. Segrelles-Calvo, G., et al., *Candida spp. co-infection in COVID-19 patients with severe pneumonia: Prevalence study and associated risk factors.* Respir Med, 2021. **188**: p. 106619.
161. Khan, M.S., et al., *Cardiovascular implications of COVID-19 versus influenza infection: a review.* BMC Medicine, 2020. **18**(1).
162. Gustafson, D., et al., *Cardiovascular signatures of COVID-19 predict mortality and identify barrier stabilizing therapies.* eBioMedicine, 2022. **78**.
163. Nasrullah, A., et al., *A case of acquired immunodeficiency syndrome-related Kaposi sarcoma in a patient with COVID-19 – A brief review of HIV-COVID Co-infection and its Therapeutic challenges!* Respiratory Medicine Case Reports, 2021. **34**.
164. Tomos, I., et al., *A case of influenza a and COVID-19 co-infection in a patient with severe asthma similarities and differences between the two viruses.* Pneumon, 2020. **33**(4): p. 1-5.
165. Yun, S., J. Kim, and H.R. Shin, *A Case Report of Varicella Zoster Meningitis as Co-Infection With Breakthrough COVID-19 in an Immunocompetent Patient.* Journal of Korean Medical Science, 2022. **37**(8).
166. Baala, L., et al., *Case Report: Co-infection with SARS-CoV-2 and influenza H1N1 in a patient with acute respiratory distress syndrome.* F1000Research, 2020. **9**: p. 1482.
167. Nasomsong, W., V. Luvira, and D. Phiboonbanakit, *Case report: Dengue and COVID-19 coinfection in Thailand.* American Journal of Tropical Medicine and Hygiene, 2021. **104**(2): p. 487-489.
168. Gjorgjievska, M., et al., *Case Report: Omicron BA.2 Subvariant of SARS-CoV-2 Outcompetes BA.1 in Two Co-infection Cases.* Frontiers in Genetics, 2022. **13**.
169. Menghua, W., et al., *Case report: One case of coronavirus disease 2019 (COVID-19) in a patient co-infected by HIV with a normal CD4+T cell count.* AIDS Research and Therapy, 2020. **17**(1).
170. Zvereva, N.N., et al., *The Cases of COVID-19 and Measles Co-Infection in Children.* Epidemiologiya i Vaktsinoprofilaktika, 2021. **20**(6): p. 81-87.
171. Farrell, J.M., et al., *Causes and Consequences of COVID-19-Associated Bacterial Infections.* Frontiers in Microbiology, 2021. **12**.
172. Yousaf, Z., et al., *Cavitary pulmonary tuberculosis with COVID-19 coinfection.* IDCases, 2020. **22**.
173. Woldemeskel, B.A., et al., *CD4+ T cells from COVID-19 mRNA vaccine recipients recognize a conserved epitope present in diverse coronaviruses.* Journal of Clinical Investigation, 2022. **132**(5).
174. Lineburg, K.E., et al., *CD8+ T cells specific for an immunodominant SARS-CoV-2 nucleocapsid epitope cross-react with selective seasonal coronaviruses.* Immunity, 2021. **54**(5): p. 1055-1065.e5.
175. Nguyen, T.H.O., et al., *CD8+ T cells specific for an immunodominant SARS-CoV-2 nucleocapsid epitope display high naive precursor frequency and TCR promiscuity.* Immunity, 2021. **54**(5): p. 1066-1082.e5.
176. Bourgoin, P., et al., *CD169 and CD64 could help differentiate bacterial from CoVID-19 or other viral infections in the Emergency Department.* Cytometry A, 2021. **99**(5): p. 435-445.
177. Lopez-Munoz, A.D., et al., *Cell surface SARS-CoV-2 nucleocapsid protein modulates innate and adaptive immunity.* Science Advances, 2022. **8**(31).
178. Vallejo, A., et al., *Cellular Responses to Membrane and Nucleocapsid Viral Proteins Are Also Boosted After SARS-CoV-2 Spike mRNA Vaccination in Individuals With Either Past Infection or Cross-Reactivity.* Frontiers in Microbiology, 2022. **12**.
179. Poulain, M., D. Chambre, and G.M. Pes, *Centenarians exposed to the Spanish flu in their early life better survived to COVID-19.* Aging, 2021. **13**(18): p. 21855-21865.
180. Bellanti, J.A. and R.A. Settipane, *The challenge of COVID-19 that permeates the practice of allergy/immunology.* Allergy and Asthma Proceedings, 2021. **42**(1): p. 1-4.

181. Steuten, K., et al., *Challenges for Targeting SARS-CoV-2 Proteases as a Therapeutic Strategy for COVID-19*. ACS Infectious Diseases, 2021. **7**(6): p. 1457-1468.
182. Rajamahanthi, Y. and G. Singh, *Challenges in management of severe COVID-19 in a post renal transplant patient co-infected with cytomegalovirus*. Bali Journal of Anesthesiology, 2021. **5**(4): p. 275-278.
183. Pradhan, A.U., et al., *Challenges of addressing neglected tropical diseases amidst the COVID-19 pandemic in Africa: A case of Chagas Disease*. Annals of Medicine and Surgery, 2022. **81**.
184. Olsen, S.J., et al., *Changes in Influenza and Other Respiratory Virus Activity During the COVID-19 Pandemic - United States, 2020-2021*. MMWR Morb Mortal Wkly Rep, 2021. **70**(29): p. 1013-1019.
185. Tang, H.J., C.C. Lai, and C.M. Chao, *Changing Epidemiology of Respiratory Tract Infection during COVID-19 Pandemic*. Antibiotics, 2022. **11**(3).
186. Kim, J., et al., *Changing influenza activity in the Southern hemisphere countries during the COVID-19 pandemic*. International Journal of Infectious Diseases, 2021. **108**: p. 109-111.
187. Agha, R. and J. Avner, *The changing pattern of respiratory viruses during covid-19—what does the future hold? BMJ Opinion. Aug 2021*.
188. Lees, E.A., et al., *Characterisation of acute respiratory infections at a United Kingdom paediatric teaching hospital: observational study assessing the impact of influenza A (2009 pdmH1N1) on predominant viral pathogens*. BMC Infect Dis, 2014. **14**: p. 343.
189. Lendorf, M.E., et al., *Characteristics and early outcomes of patients hospitalised for COVID-19 in North Zealand, Denmark*. Dan Med J, 2020. **67**(9).
190. Chi, H., et al., *Characteristics and etiology of hospitalized pediatric community-acquired pneumonia in Taiwan*. J Formos Med Assoc, 2020. **119**(10): p. 1490-1499.
191. Guntur, V.P., et al., *Characteristics and outcomes of ambulatory patients with suspected COVID-19 at a respiratory referral center*. Respir Med, 2022. **197**: p. 106832.
192. Ghazaly, M. and S. Nadel, *Characteristics of children admitted to intensive care with acute bronchiolitis*. Eur J Pediatr, 2018. **177**(6): p. 913-920.
193. Loubet, P., et al., *Characteristics of human metapneumovirus infection in adults hospitalized for community-acquired influenza-like illness in France, 2012-2018: a retrospective observational study*. Clin Microbiol Infect, 2021. **27**(1): p. 127.e1-127.e6.
194. So, W., et al., *Characteristics of procalcitonin in hospitalized COVID-19 patients and clinical outcomes of antibiotic use stratified by procalcitonin levels*. Intern Emerg Med, 2022. **17**(5): p. 1405-1412.
195. Hu, B., et al., *Characteristics of SARS-CoV-2 and COVID-19*. Nature Reviews Microbiology, 2021. **19**(3): p. 141-154.
196. Schaffner, A., et al., *Characterization of a pan-immunoglobulin assay quantifying antibodies directed against the receptor binding domain of the sars-cov-2 s1-subunit of the spike protein: A population-based study*. Journal of Clinical Medicine, 2020. **9**(12): p. 1-20.
197. Lingscheid, T., et al., *Characterization of antimicrobial use and co-infections among hospitalized patients with COVID-19: a prospective observational cohort study*. Infection, 2022.
198. Lu, R.J., et al., *[Characterization of human coronavirus 229E infection among patients with respiratory symptom in Beijing, Oct-Dec, 2007]*. Zhonghua Shi Yan He Lin Chuang Bing Du Xue Za Zhi, 2009. **23**(5): p. 367-70.
199. Borena, W., et al., *Characterization of immune responses to sars-cov-2 and other human pathogenic coronaviruses using a multiplex bead-based immunoassay*. Vaccines, 2021. **9**(6).
200. Schulien, I., et al., *Characterization of pre-existing and induced SARS-CoV-2-specific CD8(+) T cells*. Nat Med, 2021. **27**(1): p. 78-85.
201. Zeng, W., et al., *Characterization of SARS-CoV-2-specific antibodies in COVID-19 patients reveals highly potent neutralizing IgA*. Signal Transduction and Targeted Therapy, 2021. **6**(1).
202. Ou, X., et al., *Characterization of spike glycoprotein of SARS-CoV-2 on virus entry and its immune cross-reactivity with SARS-CoV*. Nature Communications, 2020. **11**(1).
203. Tai, W., et al., *Characterization of the receptor-binding domain (RBD) of 2019 novel coronavirus: implication for development of RBD protein as a viral attachment inhibitor and vaccine*. Cellular and Molecular Immunology, 2020. **17**(6): p. 613-620.
204. Dowell, A.C., et al., *Children develop robust and sustained cross-reactive spike-specific immune responses to SARS-CoV-2 infection*. Nature Immunology, 2022. **23**(1): p. 40-49.
205. Yan, Y., et al., *Clinical and epidemiological profiles including meteorological factors of low respiratory tract infection due to human rhinovirus in hospitalized children*. Ital J Pediatr, 2017. **43**(1): p. 23.
206. Chen, S., et al., *Clinical and etiological analysis of co-infections and secondary infections in COVID-19 patients: An observational study*. Clin Respir J, 2021. **15**(7): p. 815-825.
207. Zheng, J., et al., *Clinical and virological impact of single and dual infections with influenza A (H1N1) and SARS-CoV-2 in adult inpatients*. PLoS Negl Trop Dis, 2021. **15**(11): p. e0009997.
208. Lv, Z., et al., *Clinical characteristics and co-infections of 354 hospitalized patients with COVID-19 in Wuhan, China: a retrospective cohort study*. Microbes Infect, 2020. **22**(4-5): p. 195-199.
209. Tong, X., et al., *Clinical characteristics and outcome of influenza virus infection among adults hospitalized with severe COVID-19: a retrospective cohort study from Wuhan, China*. BMC Infect Dis, 2021. **21**(1): p. 341.

210. Echenique, I.A., et al., *Clinical characteristics and outcomes in hospitalized patients with respiratory viral co-infection during the 2009 H1N1 influenza pandemic.* PLoS One, 2013. **8**(4): p. e60845.
211. He, S., et al., *Clinical characteristics of "re-positive" discharged COVID-19 pneumonia patients in Wuhan, China.* Sci Rep, 2020. **10**(1): p. 17365.
212. Schneider, H., et al., *Clinical characteristics of children with viral single- and co-infections and a petechial rash.* Pediatr Infect Dis J, 2013. **32**(5): p. e186-91.
213. Ma, S., et al., *Clinical characteristics of critically ill patients co-infected with SARS-CoV-2 and the influenza virus in Wuhan, China.* International Journal of Infectious Diseases, 2020. **96**: p. 683-687.
214. Khandaker, G., et al., *Clinical epidemiology and predictors of outcome in children hospitalised with influenza A(H1N1)pdm09 in 2009: a prospective national study.* Influenza Other Respir Viruses, 2014. **8**(6): p. 636-45.
215. Wang, C., et al., *Clinical features and epidemiological analysis of respiratory human adenovirus infection in hospitalized children: a cross-sectional study in Zhejiang.* Virol J, 2021. **18**(1): p. 234.
216. Liu, Y.J., et al., *[Clinical features of asymptomatic or subclinical COVID-19 in children].* Zhongguo Dang Dai Er Ke Za Zhi, 2020. **22**(6): p. 578-582.
217. Findeisen, P., et al., *Clinical performance evaluation of a SARS-CoV-2 Rapid Antibody Test for determining past exposure to SARS-CoV-2.* International Journal of Infectious Diseases, 2021. **103**: p. 636-641.
218. Zhou, J.A., et al., *Clinical Performance of SARS-CoV-2 IgG and IgM Tests Using an Automated Chemiluminescent Assay.* Curr Med Sci, 2021. **41**(2): p. 318-322.
219. Wang, Z., et al., *Clonally diverse CD38(+)HLA-DR(+)CD8(+) T cells persist during fatal H7N9 disease.* Nat Commun, 2018. **9**(1): p. 824.
220. Sarton, B., et al., *Co-Infection and Ventilator-Associated Pneumonia in Critically Ill COVID-19 Patients Requiring Mechanical Ventilation: A Retrospective Cohort Study.* Biomedicines, 2022. **10**(8).
221. Heshmat-Ghahdarijani, K., et al., *Co-infection between the severe acute respiratory syndrome coronavirus 2 and the influenza Type B in Isfahan, Iran.* Journal of Research in Medical Sciences, 2021. **26**(1).
222. Allou, N., et al., *Co-infection in patients with hypoxemic pneumonia due to COVID-19 in Reunion Island.* Medicine (Baltimore), 2021. **100**(4): p. e24524.
223. Khodamoradi, Z., M. Moghadami, and M. Lotfi, *Co-infection of coronavirus disease 2019 and influenza a: A report from Iran.* Archives of Iranian Medicine, 2020. **23**(4): p. 239-243.
224. Jing, R., et al., *Co-infection of COVID-19 and influenza A in a hemodialysis patient: a case report.* BMC Infectious Diseases, 2021. **21**(1).
225. Cheng, Y., et al., *Co-infection of influenza A virus and SARS-CoV-2: A retrospective cohort study.* J Med Virol, 2021. **93**(5): p. 2947-2954.
226. Huang, B.R., et al., *Co-infection of influenza B virus and SARS-CoV-2: A case report from Taiwan.* Journal of Microbiology, Immunology and Infection, 2021. **54**(2): p. 336-338.
227. Wehl, G., M. Laible, and M. Rauchenzauner, *Co-infection of SARS CoV-2 and influenza A in a Pediatric Patient in Germany.* Klinische Padiatrie, 2020. **232**(4): p. 217-218.
228. Xiang, X., et al., *Co-infection of SARS-COV-2 and Influenza A Virus: A Case Series and Fast Review.* Curr Med Sci, 2021. **41**(1): p. 51-57.
229. Kinoshita, T., et al., *Co-infection of SARS-CoV-2 and influenza virus causes more severe and prolonged pneumonia in hamsters.* Scientific Reports, 2021. **11**(1).
230. Zheng, X., et al., *Co-infection of SARS-CoV-2 and Influenza virus in Early Stage of the COVID-19 Epidemic in Wuhan, China.* Journal of Infection, 2020. **81**(2): p. e128-e129.
231. Dao, T.L., et al., *Co-infection of SARS-CoV-2 and influenza viruses: A systematic review and meta-analysis.* Journal of Clinical Virology Plus, 2021. **1**(3).
232. Burrel, S., et al., *Co-infection of SARS-CoV-2 with other respiratory viruses and performance of lower respiratory tract samples for the diagnosis of COVID-19.* International Journal of Infectious Diseases, 2021. **102**: p. 10-13.
233. Arguni, E., et al., *Co-infection of SARS-CoV-2 with other viral respiratory pathogens in Yogyakarta, Indonesia: A cross-sectional study.* Annals of Medicine and Surgery, 2022. **77**.
234. Zhang, W., et al., *Co-infection with Avian (H7N9) and Pandemic (H1N1) 2009 Influenza Viruses, China.* Emerg Infect Dis, 2015. **21**(4): p. 715-8.
235. Tang, M.L., et al., *Co-Infection with Common Respiratory Pathogens and SARS-CoV-2 in Patients with COVID-19 Pneumonia and Laboratory Biochemistry Findings: A Retrospective Cross-Sectional Study of 78 Patients from a Single Center in China.* Med Sci Monit, 2021. **27**: p. e929783.
236. Hashemi, S.A., et al., *Co-infection with COVID-19 and influenza A virus in two died patients with acute respiratory syndrome, Bojnurd, Iran.* Journal of Medical Virology, 2020. **92**(11): p. 2319-2321.
237. Calcagno, A., et al., *Co-infection with other respiratory pathogens in COVID-19 patients.* Clin Microbiol Infect, 2021. **27**(2): p. 297-298.
238. Zhu, X., et al., *Co-infection with respiratory pathogens among COVID-2019 cases.* Virus Res, 2020. **285**: p. 198005.
239. Azekawa, S., et al., *Co-infection with SARS-CoV-2 and influenza A virus.* IDCases, 2020. **20**.

240. Wu, X., et al., *Co-infection with SARS-CoV-2 and influenza a virus in patient with pneumonia, China.* Emerging Infectious Diseases, 2020. **26**(6): p. 1324-1326.
241. Rodriguez, J.A., et al., *Co-Infection with SARS-COV-2 and Parainfluenza in a young adult patient with pneumonia: Case Report.* IDCases, 2020. **20**.
242. He, H., et al., *Co-infection with SARS-CoV-2 and parainfluenza virus in a hemodialysis patient: A case report.* Clinical Nephrology, 2020. **94**(4): p. 207-211.
243. Lai, C.C., C.Y. Wang, and P.R. Hsueh, *Co-infections among patients with COVID-19: The need for combination therapy with non-anti-SARS-CoV-2 agents?* Journal of Microbiology, Immunology and Infection, 2020. **53**(4): p. 505-512.
244. Huang, Y.T., et al., *Co-Infections by Double-Stranded DNA Viruses after Ex Vivo T Cell-Depleted, CD34(+) Selected Hematopoietic Cell Transplantation.* Biol Blood Marrow Transplant, 2017. **23**(10): p. 1759-1766.
245. Li, Y., et al., *Co-infections of SARS-CoV-2 with multiple common respiratory pathogens in infected children: A retrospective study.* Medicine (Baltimore), 2021. **100**(11): p. e24315.
246. Wussow, F., et al., *COH04S1 and beta sequence-modified vaccine protect hamsters from SARS-CoV-2 variants.* iScience, 2022. **25**(6).
247. Özdemir, Ö. and Ü. Dikici, *Coinfection between SARS-CoV-2 and other respiratory tract viruses.* Journal of Clinical Laboratory Analysis, 2022. **36**(6).
248. Haney, J., et al., *Coinfection by influenza A virus and respiratory syncytial virus produces hybrid virus particles.* Nature Microbiology, 2022.
249. Fahim, M., et al., *Coinfection with SARS-CoV-2 and influenza a(H1N1) in a patient seen at an influenza-like illness surveillance site in egypt: Case report.* JMIR Public Health and Surveillance, 2021. **7**(4).
250. Alhumaid, S., et al., *Coinfections with bacteria, fungi, and respiratory viruses in patients with sars-cov-2: A systematic review and meta-analysis.* Pathogens, 2021. **10**(7).
251. Jangra, S., et al., *A Combination Adjuvant for the Induction of Potent Antiviral Immune Responses for a Recombinant SARS-CoV-2 Protein Vaccine.* Frontiers in Immunology, 2021. **12**.
252. Liu, H., et al., *A combination of cross-neutralizing antibodies synergizes to prevent SARS-CoV-2 and SARS-CoV pseudovirus infection.* Cell Host and Microbe, 2021. **29**(5): p. 806-818.e6.
253. Palermo, A., et al., *Comments on: "Unexpected detection of SARS-CoV-2 antibodies in the prepandemic period in Italy".* Tumori, 2021. **107**(5): p. 470-471.
254. Kandeil, A., et al., *Common childhood vaccines do not elicit a cross-reactive antibody response against SARS-CoV-2.* PLoS ONE, 2020. **15**(10 October).
255. De la Puerta, R., et al., *Common seasonal respiratory virus infections in allogeneic stem cell transplant recipients during the SARS-COV-2 pandemic.* Bone Marrow Transplant, 2021. **56**(9): p. 2212-2220.
256. Søgaard, K.K., et al., *Community-acquired and hospital-acquired respiratory tract infection and bloodstream infection in patients hospitalized with COVID-19 pneumonia.* Journal of Intensive Care, 2021. **9**(1).
257. Wee, L.E., et al., *Community-acquired viral respiratory infections amongst hospitalized inpatients during a COVID-19 outbreak in Singapore: co-infection and clinical outcomes.* Journal of Clinical Virology, 2020. **128**.
258. Milman, O., et al., *Community-level evidence for SARS-CoV-2 vaccine protection of unvaccinated individuals.* Nature Medicine, 2021. **27**(8): p. 1367-1369.
259. Leach, S., et al., *Comparable endemic coronavirus nucleoprotein-specific antibodies in mild and severe Covid-19 patients.* Journal of Medical Virology, 2021. **93**(9): p. 5614-5617.
260. Mazzini, L., et al., *Comparative analyses of SARS-CoV-2 binding (IgG, IgM, IgA) and neutralizing antibodies from human serum samples.* Journal of Immunological Methods, 2021. **489**.
261. Belik, M., et al., *Comparative analysis of COVID-19 vaccine responses and third booster dose-induced neutralizing antibodies against Delta and Omicron variants.* Nature Communications, 2022. **13**(1).
262. Ding, Q., et al., *Comparison of clinical features of acute lower respiratory tract infections in infants with RSV/HRV infection, and incidences of subsequent wheezing or asthma in childhood.* BMC Infect Dis, 2020. **20**(1): p. 387.
263. Ekinci Sert, S., et al., *Comparison of Clinical, Demographic Features, and Costs in Respiratory Syncytial Virus, Rhinovirus, and Viral Co-infections in Children Hospitalized with Viral Infections of the Lower Respiratory Tract.* Jpn J Infect Dis, 2022. **75**(2): p. 164-168.
264. Van Praet, J.T., et al., *Comparison of four commercial SARS-CoV-2 IgG immuno-assays in RT-PCR negative patients with suspect CT findings.* Infection, 2021. **49**(1): p. 145-148.
265. Thümmler, L., et al., *Comparison of sars-cov-2-and hcov-specific t cell response using ifn-γ elispot.* Diagnostics, 2021. **11**(8).
266. Miller, N.L., et al., *Complexity of Viral Epitope Surfaces as Evasive Targets for Vaccines and Therapeutic Antibodies.* Frontiers in Immunology, 2022. **13**.
267. Hönemann, M., et al., *Comprehensive evaluation of eight commercial SARS-CoV-2 IgG assays.* Diagnostic Microbiology and Infectious Disease, 2021. **100**(4).

268. Zhang, T., et al., *Consideration on implementation of co-administration of Seasonal Influenza and COVID-19 vaccines during pandemic in China*. Zhonghua yu fang yi xue za zhi [Chinese journal of preventive medicine], 2022. **56**(2): p. 103-107.
269. Stefano, G.B. and R.M. Kream, *Convalescent memory t cell immunity in individuals with mild or asymptomatic sars-cov-2 infection may result from an evolutionarily adapted immune response to coronavirus and the 'common cold'*. Medical Science Monitor, 2020. **26**.
270. Harvala, H., et al., *Convalescent plasma donors show enhanced cross-reactive neutralizing antibody response to antigenic variants of SARS-CoV-2 following immunization*. Transfusion, 2022. **62**(7): p. 1347-1354.
271. Daisley, H., et al., *Coronavirus 229E with rhinovirus co-infection causing severe acute respiratory distress syndrome with thrombotic microangiopathy and death during Covid-19 pandemic: Lessons to be learnt*. Autopsy and Case Reports, 2020. **10**(3).
272. Gorse, G.J., et al., *Coronavirus and Other Respiratory Illnesses Comparing Older with Young Adults*. Am J Med, 2015. **128**(11): p. 1251.e11-20.
273. Collier, A.R.Y., et al., *Coronavirus Disease 2019 Messenger RNA Vaccine Immunogenicity in Immunosuppressed Individuals*. Journal of Infectious Diseases, 2022. **225**(7): p. 1124-1128.
274. Hara, M. and S. Takao, *Coronavirus Infections in Pediatric Outpatients with Febrile Respiratory Tract Infections in Hiroshima, Japan, over a 3-Year Period*. Jpn J Infect Dis, 2015. **68**(6): p. 523-5.
275. Petrie, J.G., et al., *Coronavirus Occurrence in the Household Influenza Vaccine Evaluation (HIVE) Cohort of Michigan Households: Reinfection Frequency and Serologic Responses to Seasonal and Severe Acute Respiratory Syndrome Coronaviruses*. Journal of Infectious Diseases, 2021. **224**(1): p. 49-59.
276. Sampson, A.T., et al., *Coronavirus pseudotypes for all circulating human coronaviruses for quantification of cross-neutralizing antibody responses*. Viruses, 2021. **13**(8).
277. Jacob-Dolan, C., et al., *Coronavirus-specific antibody cross reactivity in rhesus macaques following SARS-CoV-2 vaccination and infection*. Journal of Virology, 2021. **95**(11).
278. *CORONAVIRUS: Probing CD4 T cell immunity to SARS-CoV-2*. Science, 2021. **372**(6548): p. 1302B.
279. Wielgat, P., et al., *Coronaviruses: Is Sialic Acid a Gate to the Eye of Cytokine Storm? From the Entry to the Effects*. Cells, 2020. **9**(9).
280. Tang, K.H.D. and B.L.F. Chin, *Correlations between control of COVID-19 transmission and influenza occurrences in Malaysia*. Public Health, 2021. **198**: p. 96-101.
281. Pathak, S., M.K. Jolly, and D. Nandi, *Countries with high deaths due to flu and tuberculosis demonstrate lower COVID-19 mortality: roles of vaccinations*. Human Vaccines and Immunotherapeutics, 2021. **17**(9): p. 2851-2862.
282. Raychaudhuri, D., et al., *COVID-19 and Co-infection in Children: The Indian Perspectives*. J Trop Pediatr, 2021. **67**(4).
283. Bolourian, A. and Z. Mojtahedi, *COVID-19 and Flu Pandemics Follow a Pattern: A Possible Cross-immunity in the Pandemic Origin and Graver Disease in Farther Regions*. Archives of Medical Research, 2021. **52**(2): p. 240-241.
284. Dadashi, M., et al., *COVID-19 and Influenza Co-infection: A Systematic Review and Meta-Analysis*. Frontiers in medicine, 2021. **8**: p. 681469-681469.
285. Stephens, D.S. and M.J. McElrath, *COVID-19 and the Path to Immunity*. JAMA - Journal of the American Medical Association, 2020. **324**(13): p. 1279-1281.
286. Schwab, N., et al., *COVID-19 Autopsies Reveal Underreporting of SARS-CoV-2 Infection and Scarcity of Co-infections*. Frontiers in Medicine, 2022. **9**.
287. Langerbeins, P., et al., *COVID-19 complicated by parainfluenza co-infection in a patient with chronic lymphocytic leukemia*. European Journal of Haematology, 2020. **105**(4): p. 508-511.
288. Focosi, D., et al., *Covid-19 convalescent plasma is more than neutralizing antibodies: A narrative review of potential beneficial and detrimental co-factors*. Viruses, 2021. **13**(8).
289. Fontanet, A. and S. Cauchemez, *COVID-19 herd immunity: where are we?* Nature Reviews Immunology, 2020. **20**(10): p. 583-584.
290. Palmer, S., N. Cunniffe, and R. Donnelly, *COVID-19 hospitalization rates rise exponentially with age, inversely proportional to thymic T-cell production*. Journal of the Royal Society Interface, 2021. **18**(176).
291. Darwish, R.M., *COVID-19 immunity and vaccines: What a pharmacist needs to know*. Asian Biomedicine, 2021. **15**(2): p. 51-67.
292. Amiri, M.R., *COVID-19 in Children: Do They Have a Lower Risk of Severe Infection Than Adults?* Journal of Comprehensive Pediatrics, 2021. **12**(3).
293. Roberts, M.B., et al., *COVID-19 in solid organ transplant recipients: Dynamics of disease progression and inflammatory markers in ICU and non-ICU admitted patients*. Transplant Infectious Disease, 2020. **22**(5).
294. Devulapalli, C.S., *COVID-19 is milder in children possibly due to cross-immunity*. Acta Paediatrica, International Journal of Paediatrics, 2020. **109**(11): p. 2422.
295. Cañete, P.F. and C.G. Vinuesa, *COVID-19 Makes B Cells Forget, but T Cells Remember*. Cell, 2020. **183**(1): p. 13-15.

296. Jalkanen, P., et al., *COVID-19 mRNA vaccine induced antibody responses against three SARS-CoV-2 variants.* Nat Commun, 2021. **12**(1): p. 3991.
297. Secchi, M., et al., *COVID-19 survival associates with the immunoglobulin response to the SARS-CoV-2 spike receptor binding domain.* J Clin Invest, 2020. **130**(12): p. 6366-6378.
298. Fernandez-Nieto, D., et al., *COVID-19 vaccination challenge: history lessons from a dermatologist's perspective.* International Journal of Dermatology, 2021. **60**(5): p. 620-621.
299. Elko, E.A., et al., *COVID-19 vaccination elicits an evolving, cross-reactive antibody response to epitopes conserved with endemic coronavirus spike proteins.* Cell Reports, 2022. **40**(1).
300. Alhoufie, S.T., et al., *COVID-19 with underdiagnosed influenza B and parainfluenza-2 co-infections in Saudi Arabia: Two case reports.* J Infect Public Health, 2021. **14**(11): p. 1567-1570.
301. Kashir, J., K. AlKattan, and A. Yaqinuddin, *COVID-19: cross-immunity of viral epitopes may influence severity of infection and immune response.* Signal Transduction and Targeted Therapy, 2021. **6**(1).
302. Ahmad, T., et al., *COVID-19: The Emerging Immunopathological Determinants for Recovery or Death.* Frontiers in Microbiology, 2020. **11**.
303. Manselle Cocco, M.N., et al., *COVID-T: A functional platform to monitor SARS-CoV-2-specific T cell responses in vaccinated individuals and COVID-19 recovered patients.* Medicina (Argentina), 2021. **81**(5): p. 683-687.
304. Faustini, S., et al., *Cross reactivity of spike glycoprotein induced antibody against Delta and Omicron variants before and after third SARS-CoV-2 vaccine dose in healthy and immunocompromised individuals.* Journal of Infection, 2022. **84**(4): p. 579-613.
305. Antoine-Reid, T., et al., *Cross-Comparison of a Chemiluminescent Platform and a Commercial Receptor Binding Domain-Based ELISA for Detecting SARS-CoV-2 IgG.* The journal of applied laboratory medicine, 2020. **5**(6): p. 1416-1420.
306. Liu, W.J., et al., *Cross-immunity Against Avian Influenza A(H7N9) Virus in the Healthy Population Is Affected by Antigenicity-Dependent Substitutions.* J Infect Dis, 2016. **214**(12): p. 1937-1946.
307. Chakrabarti, S., et al., *Cross-immunity and trained immunity in explaining variable COVID-19 mortality—Guidance for future pandemics.* Journal of Medical Virology, 2021. **93**(7): p. 4094-4096.
308. Yaqinuddin, A., *Cross-immunity between respiratory coronaviruses may limit COVID-19 fatalities.* Medical Hypotheses, 2020. **144**.
309. Piccaluga, P.P., et al., *Cross-Immunization Against Respiratory Coronaviruses May Protect Children From SARS-CoV2: More Than a Simple Hypothesis?* Frontiers in Pediatrics, 2020. **8**.
310. Díez, J.M., et al., *Cross-neutralization activity against SARS-CoV-2 is present in currently available intravenous immunoglobulins.* Immunotherapy, 2020. **12**(17): p. 1247-1255.
311. Liu, H., et al., *Cross-Neutralization of a SARS-CoV-2 Antibody to a Functionally Conserved Site Is Mediated by Avidity.* Immunity, 2020. **53**(6): p. 1272-1280.e5.
312. Meylan, P., *Cross-neutralization of variants after SARS-CoV-2 infection despite vaccine.* Revue Medicale Suisse, 2022. **18**(766): p. 134-135.
313. Dangi, T., et al., *Cross-protective immunity following coronavirus vaccination and coronavirus infection.* Journal of Clinical Investigation, 2021. **131**(24).
314. *Cross-reactive adaptive immunity against coronaviruses in young children.* Nature Immunology, 2022. **23**(1): p. 11-12.
315. Johansson, A.M., et al., *Cross-reactive and mono-reactive SARS-CoV-2 CD4+ T cells in prepandemic and COVID-19 convalescent individuals.* PLoS Pathogens, 2021. **17**(12).
316. Grobben, M., et al., *Cross-reactive antibodies after sars-cov-2 infection and vaccination.* eLife, 2021. **10**.
317. Geanes, E.S., et al., *Cross-reactive antibodies elicited to conserved epitopes on SARS-CoV-2 spike protein after infection and vaccination.* Scientific Reports, 2022. **12**(1).
318. Guo, L., et al., *Cross-reactive antibody against human coronavirus OC43 spike protein correlates with disease severity in COVID-19 patients: a retrospective study.* Emerging Microbes and Infections, 2021. **10**(1): p. 664-676.
319. Fraley, E., et al., *Cross-reactive antibody immunity against SARS-CoV-2 in children and adults.* Cell Mol Immunol, 2021. **18**(7): p. 1826-1828.
320. Lv, H., et al., *Cross-reactive Antibody Response between SARS-CoV-2 and SARS-CoV Infections.* Cell Reports, 2020. **31**(9).
321. Schultz-Cherry, S., et al., *Cross-reactive Antibody Response to mRNA SARS-CoV-2 Vaccine after Recent COVID-19-Specific Monoclonal Antibody Therapy.* Open Forum Infectious Diseases, 2021. **8**(9).
322. Loyal, L., et al., *Cross-reactive CD4+ T cells enhance SARS-CoV-2 immune responses upon infection and vaccination.* Science, 2021. **374**(6564).
323. García-Jiménez, Á.F., et al., *Cross-reactive cellular, but not humoral, immunity is detected between OC43 and SARS-CoV-2 NPs in people not infected with SARS-CoV-2: Possible role of cTFH cells.* Journal of Leukocyte Biology, 2022. **112**(2): p. 339-346.
324. Shiakolas, A.R., et al., *Cross-reactive coronavirus antibodies with diverse epitope specificities and Fc effector functions.* Cell Reports Medicine, 2021. **2**(6).

325. Ejemel, M., et al., *A cross-reactive human IgA monoclonal antibody blocks SARS-CoV-2 spike-ACE2 interaction.* Nature Communications, 2020. **11**(1).
326. Imai, K., et al., *Cross-reactive humoral immune responses against seasonal human coronaviruses in COVID-19 patients with different disease severities.* International Journal of Infectious Diseases, 2021. **111**: p. 68-75.
327. Sealy, R.E. and J.L. Hurwitz, *Cross-reactive immune responses toward the common cold human coronaviruses and severe acute respiratory syndrome coronavirus 2 (Sars-cov-2): Mini-review and a murine study.* Microorganisms, 2021. **9**(8).
328. Pedersen, J., et al., *Cross-reactive immunity against SARS-CoV-2 N protein in Central and West Africa precedes the COVID-19 pandemic.* Scientific Reports, 2022. **12**(1).
329. Gatti, L., et al., *Cross-reactive immunity potentially drives global oscillation and opposed alternation patterns of seasonal influenza A viruses.* Sci Rep, 2022. **12**(1): p. 8883.
330. Flemming, A., *Cross-reactive memory T cells abort SARS-CoV-2 infection.* Nature Reviews Immunology, 2022. **22**(1): p. 5.
331. Lipsitch, M., et al., *Cross-reactive memory T cells and herd immunity to SARS-CoV-2.* Nature Reviews Immunology, 2020. **20**(11): p. 709-713.
332. Kundu, R., et al., *Cross-reactive memory T cells associate with protection against SARS-CoV-2 infection in COVID-19 contacts.* Nature Communications, 2022. **13**(1).
333. Zhu, Y., et al., *Cross-reactive neutralization of SARS-CoV-2 by serum antibodies from recovered SARS patients and immunized animals.* Sci Adv, 2020. **6**(45).
334. Song, G., et al., *Cross-reactive serum and memory B-cell responses to spike protein in SARS-CoV-2 and endemic coronavirus infection.* Nature Communications, 2021. **12**(1).
335. Flemming, A., *Cross-reactive tissue-resident CD8+ T cells may provide first line of defence against SARS-CoV-2.* Nature Reviews Immunology, 2021. **21**(11): p. 693.
336. Lukaszuk, K., et al., *Cross-Reactivity between Half Doses of Pfizer and AstraZeneca Vaccines—A Preliminary Study.* Vaccines, 2022. **10**(4).
337. Bates, T.A., et al., *Cross-reactivity of SARS-CoV structural protein antibodies against SARS-CoV-2.* Cell Reports, 2021. **34**(7).
338. Ma, Z., et al., *Cross-reactivity towards SARS-CoV-2: the potential role of low-pathogenic human coronaviruses.* The Lancet Microbe, 2020. **1**(4): p. e151.
339. Tajuelo, A., et al., *Cross-Recognition of SARS-CoV-2 B-Cell Epitopes with Other Betacoronavirus Nucleoproteins.* Int J Mol Sci, 2022. **23**(6).
340. Prévost, J., et al., *Cross-Sectional Evaluation of Humoral Responses against SARS-CoV-2 Spike.* Cell Reports Medicine, 2020. **1**(7).
341. Reed, S.G., *Cross-viral protection against SARS-CoV-2?* Nature Reviews Immunology, 2021. **21**(1): p. 3.
342. Joag, V., et al., *Cutting edge: Mouse sars-cov-2 epitope reveals infection and vaccine-elicited cd8 t cell responses.* Journal of Immunology, 2021. **206**(5): p. 931-935.
343. Meyer-Arndt, L., et al., *Cutting Edge: Serum but Not Mucosal Antibody Responses Are Associated with Pre-Existing SARS-CoV-2 Spike Cross-Reactive CD41 T Cells following BNT162b2 Vaccination in the Elderly.* Journal of Immunology, 2022. **208**(5): p. 1001-1005.
344. Meshram, H.S., et al., *Cytomegalovirus and Severe Acute Respiratory Syndrome Coronavirus 2 Co-infection in Renal Transplants: A Retrospective Study from a Single Center.* Saudi J Kidney Dis Transpl, 2021. **32**(4): p. 929-938.
345. Watcharananan, S.P., et al., *Cytomegalovirus, adenovirus, and polyomavirus co-infection among pediatric recipients of allogeneic stem cell transplantation: characteristics and outcome.* Pediatr Transplant, 2010. **14**(5): p. 675-81.
346. Linsky, T.W., et al., *De novo design of potent and resilient hACE2 decoys to neutralize SARS-CoV-2.* Science, 2020. **370**(6521): p. 1208-1214.
347. Beaudoin-Bussières, G., et al., *Decline of humoral responses against sars-cov-2 spike in convalescent individuals.* mBio, 2020. **11**(5): p. 1-7.
348. Lehmann, A.A., et al., *Deconvoluting the T Cell Response to SARS-CoV-2: Specificity Versus Chance and Cognate Cross-Reactivity.* Frontiers in Immunology, 2021. **12**.
349. Pablo-Marcos, D., et al., *Description of influenza B in seasonal epidemic in cantabria during the beginning of the pandemia due to SARS-CoV-2.* Revista Espanola de Quimioterapia, 2020. **33**(6): p. 444-447.
350. Ahmad, S., et al., *Design of a Novel Multi Epitope-Based Vaccine for Pandemic Coronavirus Disease (COVID-19) by Vaccinomics and Probable Prevention Strategy against Avenging Zoonotics.* European Journal of Pharmaceutical Sciences, 2020. **151**.
351. Ehara, H., *Detailed Analysis of Immune Tolerance Mechanisms to SARS-CoV-2 in Children Is Needed.* Frontiers in Pediatrics, 2021. **9**.
352. Shalash, A.O., et al., *Detection and quantification of sars-cov-2 receptor binding domain neutralization by a sensitive competitive elisa assay.* Vaccines, 2021. **9**(12).

353. Zhao, L., et al., *Detection of Antibodies Against the SARS-CoV-2 Spike Protein and Analysis of the Peripheral Blood Mononuclear Cell Transcriptomic Profile, 15 Years After Recovery From SARS.* Frontiers in Cellular and Infection Microbiology, 2021. **11**.
354. Rachow, T., et al., *Detection of community-acquired respiratory viruses in allogeneic stem-cell transplant recipients and controls-A prospective cohort study.* Transpl Infect Dis, 2020. **22**(6): p. e13415.
355. Tsukinoki, K., et al., *Detection of cross-reactive immunoglobulin A against the severe acute respiratory syndrome-coronavirus-2 spike 1 subunit in saliva.* PLoS ONE, 2021. **16**(11 November).
356. Perotin, J.M., et al., *Detection of multiple viral and bacterial infections in acute exacerbation of chronic obstructive pulmonary disease: a pilot prospective study.* J Med Virol, 2013. **85**(5): p. 866-73.
357. Chong, Y.M., et al., *Detection of respiratory viruses in adults with suspected COVID-19 in Kuala Lumpur, Malaysia.* Journal of Clinical Virology, 2021. **145**.
358. Srinivasan, A., et al., *Detection of respiratory viruses in asymptomatic children undergoing allogeneic hematopoietic cell transplantation.* Pediatr Blood Cancer, 2013. **60**(1): p. 149-51.
359. Thieme, C.J., et al., *Detection of SARS-CoV-2-specific memory B cells to delineate long-term COVID-19 immunity.* Allergy: European Journal of Allergy and Clinical Immunology, 2021. **76**(8): p. 2595-2599.
360. Shrwani, K., et al., *Detection of Serum Cross-Reactive Antibodies and Memory Response to SARS-CoV-2 in Prepandemic and Post-COVID-19 Convalescent Samples.* Journal of Infectious Diseases, 2021. **224**(8): p. 1305-1315.
361. Kumari, S., et al., *Development and validation of novel kit for quantification of SARS-CoV-2 antibodies on clinical samples.* Journal of Virological Methods, 2022. **300**.
362. Hammarström, L., et al., *Development of passive immunity against SARS-CoV-2 for management of immunodeficient patients—a perspective.* Journal of Allergy and Clinical Immunology, 2020. **146**(1): p. 58-60.
363. Ignatyev, G.M., et al., *Development of specific immunity in laboratory animals after co-immunization against seasonal influenza and COVID-19.* Zhurnal Mikrobiologii Epidemiologii i Immunobiologii, 2021. **98**(6): p. 648-656.
364. Zar, H.J., et al., *Diagnosis of community-acquired pneumonia in children: South African Thoracic Society guidelines (part 2).* South African Medical Journal, 2020. **110**(7): p. 588-593.
365. Huber, T., et al., *Diagnostic performance of four SARS-CoV-2 antibody assays in patients with COVID-19 or with bacterial and non-SARS-CoV-2 viral respiratory infections.* European Journal of Clinical Microbiology and Infectious Diseases, 2021. **40**(9): p. 1983-1997.
366. Fresco-Taboada, A., et al., *Diagnostic performance of two serological assays for the detection of SARS-CoV-2 specific antibodies: surveillance after vaccination.* Diagnostic Microbiology and Infectious Disease, 2022. **102**(4).
367. Lee, B.R., et al., *Differences in pediatric SARS-CoV-2 symptomology and Co-infection rates among COVID-19 Pandemic waves.* Journal of Clinical Virology, 2022. **154**.
368. Khoo, N.K.H., et al., *Differential immunogenicity of homologous versus heterologous boost in Ad26.COV2.S vaccine recipients.* Med, 2022. **3**(2): p. 104-118.e4.
369. Gajbhiye, R.K., et al., *Differential impact of COVID-19 in pregnant women from high-income countries and low- to middle-income countries: A systematic review and meta-analysis.* International Journal of Gynecology and Obstetrics, 2021. **155**(1): p. 48-56.
370. Rambal, V., et al., *Differential influenza H1N1-specific humoral and cellular response kinetics in kidney transplant patients.* Med Microbiol Immunol, 2014. **203**(1): p. 35-45.
371. Collier, A.Y., et al., *Differential Kinetics of immune responses elicited by Covid-19 vaccines.* New England Journal of Medicine, 2021. **385**(21): p. 2010-2012.
372. Da Silva Antunes, R., et al., *Differential T-Cell Reactivity to Endemic Coronaviruses and SARS-CoV-2 in Community and Health Care Workers.* Journal of Infectious Diseases, 2021. **224**(1): p. 70-80.
373. Fazekas de St, G. and R.G. Webster, *Disquisitions of Original Antigenic Sin. I. Evidence in man.* J Exp Med, 1966. **124**(3): p. 331-45.
374. Zohar, T. and G. Alter, *Dissecting antibody-mediated protection against SARS-CoV-2.* Nature Reviews Immunology, 2020. **20**(7): p. 392-394.
375. Khan, T., et al., *Distinct antibody repertoires against endemic human coronaviruses in children and adults.* JCI Insight, 2021. **6**(4).
376. Stoddard, C.I., et al., *Distinct Antibody Responses to Endemic Coronaviruses Pre- and Post-SARS-CoV-2 Infection in Kenyan Infants and Mothers.* Viruses, 2022. **14**(7).
377. He, X.S., et al., *Distinct patterns of B-cell activation and priming by natural influenza virus infection versus inactivated influenza vaccination.* J Infect Dis, 2015. **211**(7): p. 1051-9.
378. Assis, R., et al., *Distinct SARS-CoV-2 antibody reactivity patterns elicited by natural infection and mRNA vaccination.* npj Vaccines, 2021. **6**(1).
379. Şık, N., et al., *Distribution of Viral Respiratory Pathogens During the COVID-19 Pandemic: A Single-Center Pediatric Study from Turkey.* Turkish Archives of Pediatrics, 2022. **57**(3): p. 354-359.
380. Sasson, J.M., et al., *Diverse Humoral Immune Responses in Younger and Older Adult COVID-19 Patients.* mBio, 2021. **12**(3).

381. Chai, K.M., et al., *Dna vaccination induced protective immunity against sars cov-2 infection in hamsterss*. PLoS Neglected Tropical Diseases, 2021. **15**(5).
382. Chen, J., et al., *DNA Vaccines Expressing the Envelope and Membrane Proteins Provide Partial Protection Against SARS-CoV-2 in Mice*. Frontiers in Immunology, 2022. **13**.
383. Nasrallah, G.K., *Do preexisting antibodies against seasonal coronaviruses have a protective role against SARS-CoV-2 infections and impact on COVID-19 severity?* eBioMedicine, 2022. **76**.
384. Cohen, J., *The dream vaccine*. Science, 2021. **372**(6539): p. 227-231.
385. Burns, M.D., et al., *Durability and Cross-Reactivity of SARS-CoV-2 mRNA Vaccine in Adolescent Children*. Vaccines, 2022. **10**(4).
386. Cruz, A.T. and S.L. Zeichner, *Duration of effective antibody levels after COVID-19*. Pediatrics, 2021. **148**(3).
387. Parr, T., et al., *Dynamic causal modelling of immune heterogeneity*. Scientific Reports, 2021. **11**(1).
388. Mallon, P.W.G., et al., *Dynamic Change and Clinical Relevance of Postinfectious SARS-CoV-2 Antibody Responses*. Open Forum Infectious Diseases, 2021. **8**(8).
389. Cheemarla, N.R., et al., *Dynamic innate immune response determines susceptibility to SARS-CoV-2 infection and early replication kinetics*. Journal of Experimental Medicine, 2021. **218**(8).
390. Mandelia, Y., et al., *Dynamics and predisposition of respiratory viral co-infections in children and adults*. Clin Microbiol Infect, 2021. **27**(4): p. 631.e1-631.e6.
391. Pedro, N., et al., *Dynamics of a dual sars-cov-2 lineage co-infection on a prolonged viral shedding COVID-19 case: Insights into clinical severity and disease duration*. Microorganisms, 2021. **9**(2): p. 1-10.
392. Montague, Z., et al., *Dynamics of B cell repertoires and emergence of cross-reactive responses in patients with different severities of COVID-19*. Cell Reports, 2021. **35**(8).
393. Koblischke, M., et al., *Dynamics of CD4 T Cell and Antibody Responses in COVID-19 Patients With Different Disease Severity*. Frontiers in Medicine, 2020. **7**.
394. Kaplonek, P., et al., *Early cross-coronavirus reactive signatures of humoral immunity against COVID-19*. Science Immunology, 2021. **6**(64).
395. Simpson, C.R., et al., *Early estimation of pandemic influenza Antiviral and Vaccine Effectiveness (EAVE): use of a unique community and laboratory national data-linked cohort study*. Health Technol Assess, 2015. **19**(79): p. 1-32.
396. Lünemann, J.D., et al., *EBNA1-specific T cells from patients with multiple sclerosis cross react with myelin antigens and co-produce IFN-gamma and IL-2*. J Exp Med, 2008. **205**(8): p. 1763-73.
397. Chen, A.T., et al., *Effect of inactivated influenza vaccination on human coronavirus infection: Secondary analysis of a randomized trial in Hutterite colonies*. Vaccine, 2021. **39**(48): p. 7058-7065.
398. Jiang, S. and L. Du, *Effect of Low-Pathogenic Human Coronavirus-Specific Antibodies on SARS-CoV-2*. Trends in Immunology, 2020. **41**(10): p. 853-854.
399. Reilly, E.C., K. Lambert-Emo, and D.J. Topham, *The effects of acute neutrophil depletion on resolution of acute influenza infection, establishment of tissue resident memory (TRM), and heterosubtypic immunity*. PLoS ONE, 2016. **11**(10).
400. Greco, M., et al., *Effects of Influenza Vaccination on the Response to BNT162b2 Messenger RNA COVID-19 Vaccine in Healthcare Workers*. Journal of Clinical Medicine Research, 2021. **13**(12): p. 549-555.
401. Frumkin, L.R., et al., *Egg-Derived Anti-SARS-CoV-2 Immunoglobulin Y (IgY) With Broad Variant Activity as Intranasal Prophylaxis Against COVID-19*. Frontiers in Immunology, 2022. **13**.
402. Veldhoen, M. and J.P. Simas, *Endemic SARS-CoV-2 will maintain post-pandemic immunity*. Nature Reviews Immunology, 2021. **21**(3): p. 131-132.
403. Piñana, J.L., et al., *Epidemiologic and Clinical Characteristics of Coronavirus and Bocavirus Respiratory Infections after Allogeneic Stem Cell Transplantation: A Prospective Single-Center Study*. Biol Blood Marrow Transplant, 2018. **24**(3): p. 563-570.
404. Kong, D., et al., *Epidemiological and co-infection characteristics of common human coronaviruses in Shanghai, 2015-2020: a retrospective observational study*. Emerg Microbes Infect, 2021. **10**(1): p. 1660-1668.
405. Comte, A., et al., *Epidemiological characteristics and clinical outcomes of human rhinovirus infections in a hospitalized population. Severity is independently linked to RSV coinfection and comorbidities*. J Clin Virol, 2020. **125**: p. 104290.
406. Si, Y., et al., *Epidemiological surveillance of common respiratory viruses in patients with suspected COVID-19 in Southwest China*. BMC Infect Dis, 2020. **20**(1): p. 688.
407. Jallow, M.M., et al., *Epidemiological, clinical and genotypic features of human Metapneumovirus in patients with influenza-like illness in Senegal, 2012 to 2016*. BMC Infect Dis, 2019. **19**(1): p. 457.
408. Yue, H., et al., *The epidemiology and clinical characteristics of co-infection of SARS-CoV-2 and influenza viruses in patients during COVID-19 outbreak*. J Med Virol, 2020. **92**(11): p. 2870-2873.
409. Zeng, Z.Q., et al., *Epidemiology and clinical characteristics of human coronaviruses OC43, 229E, NL63, and HKU1: a study of hospitalized children with acute respiratory tract infection in Guangzhou, China*. Eur J Clin Microbiol Infect Dis, 2018. **37**(2): p. 363-369.
410. Chamseddine, S., et al., *Epidemiology and clinical characteristics of viral infections in hospitalized children and adolescents with cancer in Lebanon*. PLoS One, 2020. **15**(9): p. e0239258.

411. Cantais, A., et al., *Epidemiology and microbiological investigations of community-acquired pneumonia in children admitted at the emergency department of a university hospital.* J Clin Virol, 2014. **60**(4): p. 402-7.
412. Nickbakhsh, S., et al., *Epidemiology of Seasonal Coronaviruses: Establishing the Context for the Emergence of Coronavirus Disease 2019.* Journal of Infectious Diseases, 2020. **222**(1): p. 17-25.
413. Núñez-Samudio, V. and I. Landires, *Epidemiology of viral respiratory infections in a pediatric reference hospital in Central Panama.* BMC Infect Dis, 2021. **21**(1): p. 43.
414. Fairchok, M.P., et al., *Epidemiology of viral respiratory tract infections in a prospective cohort of infants and toddlers attending daycare.* J Clin Virol, 2010. **49**(1): p. 16-20.
415. Garazzino, S., et al., *Epidemiology, Clinical Features and Prognostic Factors of Pediatric SARS-CoV-2 Infection: Results From an Italian Multicenter Study.* Frontiers in Pediatrics, 2021. **9**.
416. Crotty, M.P., et al., *Epidemiology, Co-Infections, and Outcomes of Viral Pneumonia in Adults: An Observational Cohort Study.* Medicine (Baltimore), 2015. **94**(50): p. e2332.
417. Camporesi, A., et al., *Epidemiology, Microbiology and Severity of Bronchiolitis in the First Post-Lockdown Cold Season in Three Different Geographical Areas in Italy: A Prospective, Observational Study.* Children, 2022. **9**(4).
418. Stoddard, C.I., et al., *Epitope profiling reveals binding signatures of SARS-CoV-2 immune response in natural infection and cross-reactivity with endemic human CoVs.* Cell Reports, 2021. **35**(8).
419. Ladner, J.T., et al., *Epitope-resolved profiling of the SARS-CoV-2 antibody response identifies cross-reactivity with endemic human coronaviruses.* Cell Reports Medicine, 2021. **2**(1).
420. Hamamoto, I. and N. Shimasaki, *Erratum to: Erratum for "The Importance of Monitoring Viral Respiratory Infections During the COVID-19 Crisis" (Vol.17, pp. 73-81, 2022).* Journal of Disaster Research, 2022. **17**(5): p. 839-840.
421. Oumei, H., et al., *Etiology of community-acquired pneumonia in 1500 hospitalized children.* J Med Virol, 2018. **90**(3): p. 421-428.
422. Zakharenkov, I.A., et al., *[Etiology of severe community - acquired pneumonia in adults: results of the first Russian multicenter study].* Ter Arkh, 2020. **92**(1): p. 36-42.
423. Nicholson, S., et al., *Evaluation of 6 Commercial SARS-CoV-2 Serology Assays Detecting Different Antibodies for Clinical Testing and Serosurveillance.* Open Forum Infectious Diseases, 2021. **8**(7).
424. Nandakumar, V., et al., *Evaluation of a Surrogate Enzyme-Linked Immunosorbent Assay-Based Severe Acute Respiratory Syndrome Coronavirus 2 (SARS-CoV-2) cPass Neutralization Antibody Detection Assay and Correlation With Immunoglobulin G Commercial Serology Assays.* Arch Pathol Lab Med, 2021. **145**(10): p. 1212-1220.
425. Kouni, S., et al., *Evaluation of viral co-infections in hospitalized and non-hospitalized children with respiratory infections using microarrays.* Clin Microbiol Infect, 2013. **19**(8): p. 772-7.
426. Mveang Nzoghe, A., et al., *Evidence and implications of pre-existing humoral cross-reactive immunity to SARS-CoV-2.* Immunity, Inflammation and Disease, 2021. **9**(1): p. 128-133.
427. Blair, P.J., et al., *Evidence for avian H9N2 influenza virus infections among rural villagers in Cambodia.* J Infect Public Health, 2013. **6**(2): p. 69-79.
428. Sen, S.R., et al., *Evidence for deleterious effects of immunological history in SARS-CoV-2.* PLoS ONE, 2022. **17**(8 August).
429. Wratil, P.R., et al., *Evidence for increased SARS-CoV-2 susceptibility and COVID-19 severity related to pre-existing immunity to seasonal coronaviruses.* Cell Rep, 2021. **37**(13): p. 110169.
430. Loos, C., et al., *Evolution of early SARS-CoV-2 and cross-coronavirus immunity.* mSphere, 2020. **5**(5).
431. Banoun, H., *Evolution of SARS-CoV-2: Review of Mutations, Role of the Host Immune System.* Nephron, 2021. **145**(4): p. 392-403.
432. Becker, M., et al., *Exploring beyond clinical routine SARS-CoV-2 serology using MultiCoV-Ab to evaluate endemic coronavirus cross-reactivity.* Nature Communications, 2021. **12**(1).
433. Saini, S., et al., *Exploring the role of framework mutations in enabling breadth of a cross-reactive antibody (CR3022) against the SARS-CoV-2 RBD and its variants of concern.* Journal of Biomolecular Structure and Dynamics, 2022.
434. Bean, D.J. and M. Sagar, *Family matters for coronavirus disease and vaccines.* Journal of Clinical Investigation, 2021. **131**(24).
435. McNaughton, A.L., et al., *Fatal COVID-19 outcomes are associated with an antibody response targeting epitopes shared with endemic coronaviruses.* JCI Insight, 2022. **7**(13).
436. Carvalho, T., F. Krammer, and A. Iwasaki, *The first 12 months of COVID-19: a timeline of immunological insights.* Nature Reviews Immunology, 2021. **21**(4): p. 245-256.
437. Poulakou, G., et al., *First influenza season after the 2009 pandemic influenza: characteristics of intensive care unit admissions in adults and children in Vall d'Hebron Hospital.* Clin Microbiol Infect, 2012. **18**(4): p. 374-80.
438. Rodríguez, A., et al., *First influenza season after the 2009 pandemic influenza: report of the first 300 ICU admissions in Spain.* Med Intensiva, 2011. **35**(4): p. 208-16.
439. Kelly, P.M., et al., *FluCAN 2009: initial results from sentinel surveillance for adult influenza and pneumonia in eight Australian hospitals.* Med J Aust, 2011. **194**(4): p. 169-74.

440. Embong, A.K., et al., *Formation and Expansion of Memory B Cells against Coronavirus in Acutely Infected COVID-19 Individuals*. Pathogens, 2022. **11**(2).
441. Ishiguro, T., et al., *Frequency and Significance of Coinfection in Patients with COVID-19 at Hospital Admission*. Intern Med, 2021. **60**(23): p. 3709-3719.
442. Boschiero, M.N., et al., *Frequency of respiratory pathogens other than SARS-CoV-2 detected during COVID-19 testing*. Diagnostic Microbiology and Infectious Disease, 2022. **102**(2).
443. Kanduc, D., *From anti-SARS-CoV-2 immune responses to COVID-19 via molecular mimicry*. Antibodies, 2020. **9**(3): p. 1-12.
444. Vanderheijden, N., et al., *Functional Analysis of Human and Feline Coronavirus Cross-Reactive Antibodies Directed Against the SARS-CoV-2 Fusion Peptide*. Frontiers in Immunology, 2021. **12**.
445. Dykema, A.G., et al., *Functional characterization of CD4+ T cell receptors crossreactive for SARS-CoV-2 and endemic coronaviruses*. Journal of Clinical Investigation, 2021. **131**(10).
446. Duray, A., et al., *Galectin fingerprinting in naso-sinusal diseases*. Oncol Rep, 2014. **32**(1): p. 23-32.
447. Zhou, H.Y., et al., *Genomic evidence for divergent co-infections of co-circulating SARS-CoV-2 lineages*. Computational and Structural Biotechnology Journal, 2022. **20**: p. 4015-4024.
448. Muelenaer, P.M., et al., *Group-specific serum antibody responses in children with primary and recurrent respiratory syncytial virus infections*. J Infect Dis, 1991. **164**(1): p. 15-21.
449. Steiner, S., et al., *HCoV- and SARS-CoV-2 Cross-Reactive T Cells in CVID Patients*. Frontiers in Immunology, 2020. **11**.
450. Simula, E.R., et al., *HCoV-NL63 and SARS-CoV-2 share recognized epitopes by the humoral response in sera of people collected pre-and during CoV-2 pandemic*. Microorganisms, 2020. **8**(12): p. 1-15.
451. Ansaldi, F., et al., *Head-to-head comparison of an intradermal and a virosome influenza vaccine in patients over the age of 60: evaluation of immunogenicity, cross-protection, safety and tolerability*. Hum Vaccin Immunother, 2013. **9**(3): p. 591-8.
452. Gouma, S., et al., *Health care worker seromonitoring reveals complex relationships between common coronavirus antibodies and COVID-19 symptom duration*. JCI Insight, 2021. **6**(16).
453. Woldemeskel, B.A., et al., *Healthy donor T cell responses to common cold coronaviruses and SARS-CoV-2*. Journal of Clinical Investigation, 2020. **130**(12): p. 6631-6638.
454. Ng, K.W., et al., *Heterologous humoral immunity to human and zoonotic coronaviruses: Aiming for the achilles heel*. Seminars in Immunology, 2021. **55**.
455. Smit, W.L., et al., *Heterologous Immune Responses of Serum IgG and Secretory IgA Against the Spike Protein of Endemic Coronaviruses During Severe COVID-19*. Frontiers in Immunology, 2022. **13**.
456. Patel, R.S. and B. Agrawal, *Heterologous immunity induced by 1st generation COVID-19 vaccines and its role in developing a pan-coronavirus vaccine*. Frontiers in Immunology, 2022. **13**.
457. Reynolds, C.J., et al., *Heterologous infection and vaccination shapes immunity against SARS-CoV-2 variants*. Science, 2022. **375**(6577): p. 183-192.
458. Rai, S., B. Shamantha Rai, and P. Rithesh Pakkala, *Heterologous Prime-boost Approach for COVID-19 Vaccine-A Light at the End of the Tunnel?* Pharmacologyonline, 2021. **3**: p. 471-483.
459. Shaw, R.H., et al., *Heterologous prime-boost COVID-19 vaccination: initial reactogenicity data*. The Lancet, 2021. **397**(10289): p. 2043-2046.
460. Tenbusch, M., et al., *Heterologous prime–boost vaccination with ChAdOx1 nCoV-19 and BNT162b2*. The Lancet Infectious Diseases, 2021. **21**(9): p. 1212-1213.
461. Hupert, N., et al., *Heterologous vaccination interventions to reduce pandemic morbidity and mortality: Modeling the US winter 2020 COVID-19 wave*. Proceedings of the National Academy of Sciences of the United States of America, 2022. **119**(3).
462. Marín-Hernández, D., D.F. Nixon, and N. Hupert, *Heterologous vaccine interventions: boosting immunity against future pandemics*. Molecular Medicine, 2021. **27**(1).
463. Budziar, W., et al., *Hidden fraction of Polish population immune to SARS-CoV-2 in May 2021*. PLoS ONE, 2022. **17**(2 February).
464. Mairesse, A., et al., *High clinical performance and quantitative assessment of antibody kinetics using a dual recognition assay for the detection of SARS-CoV-2 IgM and IgG antibodies*. Clin Biochem, 2020. **86**: p. 23-27.
465. Ghietto, L.M., et al., *High frequency of human bocavirus 1 DNA in infants and adults with lower acute respiratory infection*. J Med Microbiol, 2012. **61**(Pt 4): p. 548-551.
466. Petat, H., et al., *High Frequency of Viral Co-Detections in Acute Bronchiolitis*. Viruses, 2021. **13**(6).
467. Greenbaum, U., et al., *High Levels of Common Cold Coronavirus Antibodies in Convalescent Plasma Are Associated With Improved Survival in COVID-19 Patients*. Frontiers in Immunology, 2021. **12**.
468. Tso, F.Y., et al., *High prevalence of pre-existing serological cross-reactivity against severe acute respiratory syndrome coronavirus-2 (SARS-CoV-2) in sub-Saharan Africa*. International Journal of Infectious Diseases, 2021. **102**: p. 577-583.
469. Pape, K.A., et al., *High-affinity memory B cells induced by SARS-CoV-2 infection produce more plasmablasts and atypical memory B cells than those primed by mRNA vaccines*. Cell Reports, 2021. **37**(2).

470. Zhang, D., et al., *A high-throughput microsphere-based immunoassay of anti-SARS-CoV-2 IgM testing for COVID-19 diagnostics*. PLoS ONE, 2021. **16**(9 September).
471. Cihakova, D., et al., *High-value laboratory testing for hospitalized COVID-19 patients: A review*. Future Virology, 2021. **16**(10): p. 691-705.
472. Gao, F., et al., *A Highly Conserved Peptide Vaccine Candidate Activates Both Humoral and Cellular Immunity Against SARS-CoV-2 Variant Strains*. Frontiers in Immunology, 2021. **12**.
473. Meyers, L.M., et al., *Highly conserved, non-human-like, and cross-reactive SARS-CoV-2 T cell epitopes for COVID-19 vaccine design and validation*. npj Vaccines, 2021. **6**(1).
474. Rashid, F., et al., *Hip fracture mortality in patients co-infected with coronavirus disease 2019: a comparison of the first two waves of the United Kingdom pandemic during the pre-vaccine era*. International Orthopaedics, 2022. **46**(2): p. 171-178.
475. Nesterenko, P.A., et al., *HLA-A*02:01 restricted T cell receptors against the highly conserved SARS-CoV-2 polymerase cross-react with human coronaviruses*. Cell Reports, 2021. **37**(13).
476. Buckley, P.R., et al., *HLA-dependent variation in SARS-CoV-2 CD8 + T cell cross-reactivity with human coronaviruses*. Immunology, 2022. **166**(1): p. 78-103.
477. He, X., et al., *A homologous or variant booster vaccine after Ad26.COV2.S immunization enhances SARS-CoV-2-specific immune responses in rhesus macaques*. Science Translational Medicine, 2022. **14**(638).
478. Liu, W., et al., *Host immune response to A(H1N1)pdm09 vaccination and infection: a one-year prospective study on six cohorts of subjects*. Vaccine, 2012. **30**(32): p. 4785-9.
479. Park, J.J., et al., *How COVID-19 has fundamentally changed clinical research in global health*. The Lancet Global Health, 2021. **9**(5): p. e711-e720.
480. Waterlow, N.R., et al., *How immunity from and interaction with seasonal coronaviruses can shape SARS-CoV-2 epidemiology*. Proceedings of the National Academy of Sciences of the United States of America, 2021. **118**(49).
481. Zhou, P., et al., *A human antibody reveals a conserved site on beta-coronavirus spike proteins and confers protection against SARS-CoV-2 infection*. Science Translational Medicine, 2022. **14**(637).
482. Nielsen, S.C.A., et al., *Human B Cell Clonal Expansion and Convergent Antibody Responses to SARS-CoV-2*. Cell Host and Microbe, 2020. **28**(4): p. 516-525.e5.
483. Gelder, C.M., et al., *Human CD4+ T-cell repertoire of responses to influenza A virus hemagglutinin after recent natural infection*. J Virol, 1995. **69**(12): p. 7497-506.
484. Baloch, Z., et al., *Human Coronavirus Spike Protein Based Multi-Epitope Vaccine against COVID-19 and Potential Future Zoonotic Coronaviruses by Using Immunoinformatic Approaches*. Vaccines, 2022. **10**(7).
485. Guido, M., et al., *Human metapneumovirus and human bocavirus associated with respiratory infection in Apulian population*. Virology, 2011. **417**(1): p. 64-70.
486. Xiao, C., et al., *Human post-infection serological response to the spike and nucleocapsid proteins of SARS-CoV-2*. Influenza and other Respiratory Viruses, 2021. **15**(1): p. 7-12.
487. Barbosa Ramirez, J., et al., *Human respiratory syncytial virus and metapneumovirus in patients with acute respiratory infection in Colombia, 2000 - 2011*. Rev Panam Salud Publica, 2014. **36**(2): p. 101-9.
488. Dhanasekaran, V., et al., *Human seasonal influenza under COVID-19 and the potential consequences of influenza lineage elimination*. Nature Communications, 2022. **13**(1): p. 1721.
489. Wan, J., et al., *Human-IgG-Neutralizing Monoclonal Antibodies Block the SARS-CoV-2 Infection*. Cell Reports, 2020. **32**(3).
490. Wagar, L.E., et al., *Humoral and cell-mediated immunity to pandemic H1N1 influenza in a Canadian cohort one year post-pandemic: implications for vaccination*. PLoS One, 2011. **6**(11): p. e28063.
491. Garland, P., et al., *Humoral and cellular immunity to primary H1N1 infection in patients with hematologic malignancies following stem cell transplantation*. Biol Blood Marrow Transplant, 2011. **17**(5): p. 632-9.
492. Dhochak, N., et al., *Humoral cross-reactivity towards SARS-CoV-2 in young children with acute respiratory infection with low-pathogenicity coronaviruses*. Journal of Clinical Virology Plus, 2022. **2**(1).
493. Lee, E. and J.E. Oh, *Humoral immunity against SARS-CoV-2 and the impact on COVID-19 pathogenesis*. Molecules and Cells, 2021. **44**(6): p. 392-400.
494. Qi, H., et al., *The humoral response and antibodies against SARS-CoV-2 infection*. Nature Immunology, 2022. **23**(7): p. 1008-1020.
495. Adams, O., et al., *Humoral response to SARS-CoV-2 and seasonal coronaviruses in COVID-19 patients*. Journal of Medical Virology, 2022. **94**(3): p. 1096-1103.
496. Galipeau, Y., et al., *Humoral Responses and Serological Assays in SARS-CoV-2 Infections*. Frontiers in Immunology, 2020. **11**.
497. Achiron, A., et al., *Humoral SARS-COV-2 IgG decay within 6 months in COVID-19 healthy vaccinees: The need for a booster vaccine dose?* European Journal of Internal Medicine, 2021. **94**: p. 105-107.
498. Corral-Lugo, A., et al., *Identification and analysis of unstructured, linear b-cell epitopes in SARS-CoV-2 virion proteins for vaccine development*. Vaccines, 2020. **8**(3): p. 1-21.
499. Ianevski, A., et al., *Identification and tracking of antiviral drug combinations*. Viruses, 2020. **12**(10).
500. Powell, T.J., et al., *Identification of H5N1-specific T-cell responses in a high-risk cohort in vietnam indicates the existence of potential asymptomatic infections*. J Infect Dis, 2012. **205**(1): p. 20-7.

501. Pushpakumara, P.D., et al., *Identification of novel candidate cd8+ t cell epitopes of the sars-cov2 with homology to other seasonal coronaviruses*. Viruses, 2021. **13**(6).
502. Hardick, J., et al., *Identification of pathogens from the upper respiratory tract of adult emergency department patients at high risk for influenza complications in a pre-Sars-CoV-2 environment*. Diagnostic Microbiology and Infectious Disease, 2021. **100**(2).
503. Tai, W., et al., *Identification of SARS-CoV RBD-targeting monoclonal antibodies with cross-reactive or neutralizing activity against SARS-CoV-2*. Antiviral Research, 2020. **179**.
504. Lee, E., et al., *Identification of SARS-CoV-2 nucleocapsid and spike T-cell epitopes for assessing T-cell immunity*. Journal of Virology, 2021. **95**(6).
505. Kesarwani, V., et al., *Identification of Unique Peptides for SARS-CoV-2 Diagnostics and Vaccine Development by an In Silico Proteomics Approach*. Frontiers in Immunology, 2021. **12**.
506. Abreu, R.B., et al., *IgA Responses Following Recurrent Influenza Virus Vaccination*. Front Immunol, 2020. **11**: p. 902.
507. Wang, J., et al., *IgG Against Human Betacoronavirus Spike Proteins Correlates With SARS-CoV-2 Anti-Spike IgG Responses and COVID-19 Disease Severity*. The Journal of infectious diseases, 2022. **226**(3): p. 474-484.
508. Kazachinskaia, E., et al., *IgG study of blood sera of patients with COVID-19*. Pathogens, 2021. **10**(11).
509. Garrido, J.L., et al., *IgG targeting distinct seasonal coronavirus- conserved SARS-CoV-2 spike subdomains correlates with differential COVID-19 disease outcomes*. Cell Reports, 2022. **39**(9).
510. Saad-Roy, C.M., et al., *Immune life history, vaccination, and the dynamics of SARS-CoV-2 over the next 5 years*. Science, 2020. **370**(6518): p. 811-818.
511. Long, Q.X., et al., *Immune memory in convalescent patients with asymptomatic or mild COVID-19*. Cell Discovery, 2021. **7**(1).
512. Ansari, A., et al., *Immune Memory in Mild COVID-19 Patients and Unexposed Donors Reveals Persistent T Cell Responses After SARS-CoV-2 Infection*. Front Immunol, 2021. **12**: p. 636768.
513. Ravindran, R., et al., *Immune response dynamics in COVID-19 patients to SARS-CoV-2 and other human coronaviruses*. PLoS ONE, 2021. **16**(7 July).
514. Azkur, A.K., et al., *Immune response to SARS-CoV-2 and mechanisms of immunopathological changes in COVID-19*. Allergy: European Journal of Allergy and Clinical Immunology, 2020. **75**(7): p. 1564-1581.
515. Trombetta, C.M., et al., *Immune response to SARS-CoV-2 Omicron variant in patients and vaccinees following homologous and heterologous vaccinations*. Communications biology, 2022. **5**(1): p. 903.
516. Cluff, E., et al., *Immune Response to SARS-CoV-2 Vaccine and Following Breakthrough Omicron Infection in an Autoimmune Patient with Hashimoto's Thyroiditis, Pernicious Anemia, and Chronic Atrophic Autoimmune Gastritis: A Case Report*. Vaccines, 2022. **10**(3).
517. Becker, M., et al., *Immune response to SARS-CoV-2 variants of concern in vaccinated individuals*. Nature Communications, 2021. **12**(1).
518. Alefishat, E., et al., *Immune response to SARS-CoV-2 variants: A focus on severity, susceptibility, and preexisting immunity*. Journal of Infection and Public Health, 2022. **15**(2): p. 277-288.
519. Mohn, K.G., et al., *Immune Responses in Acute and Convalescent Patients with Mild, Moderate and Severe Disease during the 2009 Influenza Pandemic in Norway*. PLoS One, 2015. **10**(11): p. e0143281.
520. Prompetchara, E., C. Ketloy, and T. Palaga, *Immune responses in COVID-19 and potential vaccines: Lessons learned from SARS and MERS epidemic*. Asian Pacific Journal of Allergy and Immunology, 2020. **38**(1): p. 1-9.
521. Kreijtz, J.H.C.M., R.A.M. Fouchier, and G.F. Rimmelzwaan, *Immune responses to influenza virus infection*. Virus Research, 2011. **162**(1-2): p. 19-30.
522. Hernández, C.R. and J.C.S. Moreno, *Immunity against SARS-CoV-2: Walking to the vaccination*. Revista Espanola de Quimioterapia, 2020. **33**(6): p. 392-398.
523. Yamaguchi, T., et al., *Immunity against seasonal human coronavirus OC43 mitigates fatal deterioration of COVID-19*. International Journal of Infectious Diseases, 2021. **109**: p. 261-268.
524. Tostanoski, L.H., et al., *Immunity elicited by natural infection or Ad26.COV2.S vaccination protects hamsters against SARS-CoV-2 variants of concern*. Science Translational Medicine, 2021. **13**(618).
525. Fergie, J. and A. Srivastava, *Immunity to SARS-CoV-2: Lessons Learned*. Frontiers in Immunology, 2021. **12**.
526. Herrera, L.R.M., *Immuno informatics approach in designing a novel vaccine using epitopes from all the structural proteins of SARS-CoV-2*. Biomedical and Pharmacology Journal, 2020. **13**(4): p. 1845-1862.
527. Levy, Y., et al., *Immunodominant Linear B-Cell Epitopes of SARS-CoV-2 Spike, Identified by Sera from K18-hACE2 Mice Infected with the WT or Variant Viruses*. Vaccines, 2022. **10**(2).
528. Quiros-Fernandez, I., et al., *Immunogenic T cell epitopes of SARS-CoV-2 are recognized by circulating memory and naïve CD8 T cells of unexposed individuals*. EBioMedicine, 2021. **72**.
529. Dobaño, C., et al., *Immunogenicity and crossreactivity of antibodies to the nucleocapsid protein of SARS-CoV-2: utility and limitations in seroprevalence and immunity studies*. Translational Research, 2021. **232**: p. 60-74.

530. Moghnieh, R., et al., *Immunogenicity and reactogenicity of BNT162b2 booster in BBIBP-CorV-vaccinated individuals compared with homologous BNT162b2 vaccination: Results of a pilot prospective cohort study from Lebanon*. Vaccine, 2021. **39**(46): p. 6713-6719.
531. Schmidt, T., et al., *Immunogenicity and reactogenicity of heterologous ChAdOx1 nCoV-19/mRNA vaccination*. Nature Medicine, 2021. **27**(9): p. 1530-1535.
532. Collier, A.R.Y., et al., *Immunogenicity of COVID-19 mRNA Vaccines in Pregnant and Lactating Women*. JAMA - Journal of the American Medical Association, 2021. **325**(23): p. 2370-2380.
533. do Carmo Debur, M., et al., *Immunohistochemical assessment of respiratory viruses in necropsy samples from lethal non-pandemic seasonal respiratory infections*. J Clin Pathol, 2010. **63**(10): p. 930-4.
534. Oliveira, S.C., M.T.Q. de Magalhães, and E.J. Homan, *Immunoinformatic Analysis of SARS-CoV-2 Nucleocapsid Protein and Identification of COVID-19 Vaccine Targets*. Frontiers in Immunology, 2020. **11**.
535. Tilocca, B., et al., *Immunoinformatic analysis of the SARS-CoV-2 envelope protein as a strategy to assess cross-protection against COVID-19*. Microbes and Infection, 2020. **22**(4-5): p. 182-187.
536. De Mesa Herrera, L.R., *Immunoinformatics approach in designing SARS-CoV-2 vaccine from experimentally determined SARS-CoV T-cell epitopes*. Journal of Applied Pharmaceutical Science, 2021. **11**(3): p. 29-36.
537. Mathew, S., et al., *Immunoinformatics prediction of potential immunodominant epitopes from human coronaviruses and association with autoimmunity*. Immunogenetics, 2022. **74**(2): p. 213-229.
538. Li, D. and J. Li, *Immunologic testing for SARS-CoV-2 infection from the antigen perspective*. Journal of Clinical Microbiology, 2021. **59**(5).
539. Kedl, R.M., *An immunological autobiography: my year as a COVID-19 vaccine trial participant*. npj Vaccines, 2022. **7**(1).
540. Jeyanathan, M., et al., *Immunological considerations for COVID-19 vaccine strategies*. Nature Reviews Immunology, 2020. **20**(10): p. 615-632.
541. Aydillo, T., et al., *Immunological imprinting of the antibody response in COVID-19 patients*. Nature Communications, 2021. **12**(1).
542. Khorramdelazad, H., et al., *Immunopathological similarities between COVID-19 and influenza: Investigating the consequences of Co-infection*. Microbial Pathogenesis, 2021. **152**.
543. Zheng, Y., R. Li, and S. Liu, *Immunoregulation with mTOR inhibitors to prevent COVID-19 severity: A novel intervention strategy beyond vaccines and specific antiviral medicines*. Journal of Medical Virology, 2020. **92**(9): p. 1495-1500.
544. Schiaffino, M.T., et al., *Immunoserologic Detection and Diagnostic Relevance of Cross-Reactive Autoantibodies in Coronavirus Disease 2019 Patients*. J Infect Dis, 2020. **222**(9): p. 1439-1443.
545. Li, Q., et al., *Impact Of China's Covid-19 Prevention And Control Efforts On Outbreaks Of Influenza*. BioScience Trends, 2021. **15**(3): p. 192-195.
546. Sarkar, S., P. Khanna, and A.K. Singh, *Impact of COVID-19 in patients with concurrent co-infections: A systematic review and meta-analyses*. Journal of Medical Virology, 2021. **93**(4): p. 2385-2395.
547. Agarwal, A., et al., *Impact of influenza A co-infection with COVID-19*. International Journal of Tuberculosis and Lung Disease, 2021. **25**(5): p. 413-415.
548. Chen, L.L., et al., *Impact of Severe Acute Respiratory Syndrome Coronavirus 2 (SARS-CoV-2) Variant-Associated Receptor Binding Domain (RBD) Mutations on the Susceptibility to Serum Antibodies Elicited by Coronavirus Disease 2019 (COVID-19) Infection or Vaccination*. Clinical Infectious Diseases, 2022. **74**(9): p. 1623-1630.
549. Conlon, A., et al., *Impact of the influenza vaccine on COVID-19 infection rates and severity*. Am J Infect Control, 2021. **49**(6): p. 694-700.
550. Park, S.H., *An impaired inflammatory and innate immune response in COVID-19*. Molecules and Cells, 2021. **44**(6): p. 384-391.
551. Dhawan, M., T.B. Emran, and O.P. Choudhary, *Implications of COVID-19 vaccine boosters amid the emergence of novel variants of SARS-CoV-2*. Annals of Medicine and Surgery, 2022. **77**.
552. Lee, C.H., et al., *In silico identification of vaccine targets for 2019-nCoV*. F1000Research, 2020. **9**: p. 1-10.
553. Silva-Arrieta, S., P.J.R. Goulder, and C. Brander, *In silico veritas? Potential limitations for SARSCoV-2 vaccine development based on T-cell epitope prediction*. PLoS Pathogens, 2020. **16**(6).
554. Marguet, C., et al., *In very young infants severity of acute bronchiolitis depends on carried viruses*. PLoS One, 2009. **4**(2): p. e4596.
555. Gendrot, M., et al., *In Vitro Antiviral Activity of Doxycycline against SARS-CoV-2*. Molecules, 2020. **25**(21).
556. Liu, M.Q., et al., *Inactivated SARS-CoV-2 Vaccine Shows Cross-Protection against Bat SARS-Related Coronaviruses in Human ACE2 Transgenic Mice*. Journal of Virology, 2022. **96**(8).
557. Lee, V.J., et al., *Inactivated trivalent seasonal influenza vaccine induces limited cross-reactive neutralizing antibody responses against 2009 pandemic and 1934 PR8 H1N1 strains*. Vaccine, 2010. **28**(42): p. 6852-7.
558. Ham, J.Y., K.E. Song, and N.Y. Lee, *Incidence and Distribution of Respiratory Microorganisms Causing Acute Respiratory Infections at the University Hospital of Korea*. Clin Lab, 2020. **66**(12).
559. Garcia-Vidal, C., et al., *Incidence of co-infections and superinfections in hospitalized patients with COVID-19: a retrospective cohort study*. Clin Microbiol Infect, 2021. **27**(1): p. 83-88.

560. Shields, A.M., et al., *Increased Seroprevalence and Improved Antibody Responses Following Third Primary SARS-CoV-2 Immunisation: An Update From the COV-AD Study*. Frontiers in Immunology, 2022. **13**.
561. Chang, X., et al., *Induction of Broadly Cross-Reactive Antibodies by Displaying Receptor Binding Domains of SARS-CoV-2 on Virus-like Particles*. Vaccines, 2022. **10**(2).
562. Sun, C., et al., *Induction of Broadly Cross-Reactive Antibody Responses to SARS-CoV-2 Variants by S1 Nanoparticle Vaccines*. Journal of Virology, 2022. **96**(13).
563. Kositanont, U., et al., *Induction of cross-neutralizing antibody against H5N1 virus after vaccination with seasonal influenza vaccine in COPD patients*. Viral Immunol, 2010. **23**(3): p. 329-34.
564. Rouchka, E.C., et al., *Induction of interferon response by high viral loads at early stage infection may protect against severe outcomes in COVID-19 patients*. Scientific Reports, 2021. **11**(1).
565. Romero-Olmedo, A.J., et al., *Induction of robust cellular and humoral immunity against SARS-CoV-2 after a third dose of BNT162b2 vaccine in previously unresponsive older adults*. Nature Microbiology, 2022. **7**(2): p. 195-199.
566. Debisarun, P.A., et al., *Induction of trained immunity by influenza vaccination - Impact on COVID-19*. PLoS Pathogens, 2021. **17**(10).
567. Albrecht, M., et al., *Infant immunity against viral infections is advanced by the placenta-dependent vertical transfer of maternal antibodies*. Vaccine, 2022. **40**(11): p. 1563-1571.
568. Fortmann, I., et al., *Infants Younger Than 90 Days Admitted for Late-Onset Sepsis Display a Reduced Abundance of Regulatory T Cells*. Front Immunol, 2021. **12**: p. 666447.
569. Rizzo, K.R., et al., *Infl uenza and sars-cov-2 co-infections in california, usa, september 2020-april 2021*. Emerging Infectious Diseases, 2021. **27**(11): p. 2923-2926.
570. *The Influence of Immune Immaturity on Outcome After Virus Infections*. Journal of Allergy and Clinical Immunology: In Practice, 2021. **9**(2): p. 651-652.
571. Gilbert, G.L., et al., *Influenza A (H1N1) 2009 antibodies in residents of New South Wales, Australia, after the first pandemic wave in the 2009 southern hemisphere winter*. PLoS One, 2010. **5**(9): p. e12562.
572. Pérez-García, F., et al., *Influenza A and B co-infection: a case-control study and review of the literature*. Eur J Clin Microbiol Infect Dis, 2016. **35**(6): p. 941-6.
573. Drori, Y., et al., *Influenza A Virus Inhibits RSV Infection via a Two-Wave Expression of IFIT Proteins*. Viruses, 2020. **12**(10).
574. Fage, C., et al., *Influenza A(H1N1)pdm09 Virus but Not Respiratory Syncytial Virus Interferes with SARS-CoV-2 Replication during Sequential Infections in Human Nasal Epithelial Cells*. Viruses, 2022. **14**(2).
575. Bartolo, N.S., et al., *Influenza and COVID-19 vaccine hesitancy in pharmacists and pharmacy students*. Malta Medical Journal, 2022. **34**(1): p. 58-68.
576. Fritsch, A., B. Schweiger, and B. Biere, *Influenza C virus in pre-school children with respiratory infections: retrospective analysis of data from the national influenza surveillance system in Germany, 2012 to 2014*. Euro Surveill, 2019. **24**(10).
577. Alosaimi, B., et al., *Influenza co-infection associated with severity and mortality in COVID-19 patients*. Virology Journal, 2021. **18**(1).
578. Arcana, R.I.D., et al., *Influenza vaccine in COVID-19 patients: Who?, why?, when?* Pneumologia, 2021. **69**(3): p. 151-158.
579. Beumer, M.C., et al., *Influenza virus and factors that are associated with ICU admission, pulmonary co-infections and ICU mortality*. J Crit Care, 2019. **50**: p. 59-65.
580. Sandor, A.M., M.S. Sturdivant, and J.P.Y. Ting, *Influenza virus and sars-cov-2 vaccines*. Journal of Immunology, 2021. **206**(11): p. 2509-2520.
581. Cruz-Cañete, M., et al., *[Influenza virus in pediatrics. A reason for hospitalization]*. Enferm Infecc Microbiol Clin, 2007. **25**(3): p. 177-83.
582. Liao, Q., et al., *Inhaled Dry Powder Formulation of Tamibarotene, a Broad-Spectrum Antiviral against Respiratory Viruses Including SARS-CoV-2 and Influenza Virus*. Advanced Therapeutics, 2021. **4**(7).
583. Ramasamy, R., *Innate and Adaptive Immune Responses in the Upper Respiratory Tract and the Infectivity of SARS-CoV-2*. Viruses, 2022. **14**(5).
584. Jordan, S.C., *Innate and adaptive immune responses to SARS-CoV-2 in humans: relevance to acquired immunity and vaccine responses*. Clinical and Experimental Immunology, 2021. **204**(3): p. 310-320.
585. Diamond, M.S. and T.-D. Kanneganti, *Innate immunity: the first line of defense against SARS-CoV-2*. Nature Immunology, 2022. **23**(2): p. 165-176.
586. Andrade, V.M., et al., *INO-4800 DNA vaccine induces neutralizing antibodies and T cell activity against global SARS-CoV-2 variants*. npj Vaccines, 2021. **6**(1).
587. Ward, D., et al., *An integrated in silico immuno-genetic analytical platform provides insights into COVID-19 serological and vaccine targets*. Genome Medicine, 2021. **13**(1).
588. Pascalis, H., et al., *Intense co-circulation of non-influenza respiratory viruses during the first wave of pandemic influenza pH1N1/2009: a cohort study in Reunion Island*. PLoS One, 2012. **7**(9): p. e44755.
589. Ehrlich, H., D. Boneva, and A. Elkbuli, *The intersection of viral illnesses: A seasonal influenza epidemic amidst the COVID-19 pandemic*. Annals of Medicine and Surgery, 2020. **60**: p. 41-43.

590. Vesin, B., et al., *An intranasal lentiviral booster reinforces the waning mRNA vaccine-induced SARS-CoV-2 immunity that it targets to lung mucosa*. Molecular Therapy, 2022.
591. Fehervari, Z., *Intranasal vaccination*. Nature Immunology, 2021. **22**(9): p. 1071.
592. Hassan, A.O., et al., *An intranasal vaccine durably protects against SARS-CoV-2 variants in mice*. Cell Reports, 2021. **36**(4).
593. Raffaelli, F., et al., *Invasive Respiratory Fungal Infections in COVID-19 Critically Ill Patients*. Journal of Fungi, 2022. **8**(4).
594. Al-Dulaimi, A., et al., *Investigating the human rhinovirus co-infection in patients with asthma exacerbations and COVID-19*. Pharmacy Practice, 2022. **20**(2).
595. Chan, K.F., et al., *Investigating Viral Interference Between Influenza A Virus and Human Respiratory Syncytial Virus in a Ferret Model of Infection*. J Infect Dis, 2018. **218**(3): p. 406-417.
596. Bakir, A., et al., *Investigation of human bocavirus in pediatric patients with respiratory tract infection*. J Infect Dev Ctries, 2020. **14**(10): p. 1191-1196.
597. Kim, G.Y., et al., *Investigation of occurrence patterns of respiratory syncytial virus A and B in infected-patients from Cheonan, Korea*. Respir Res, 2020. **21**(1): p. 191.
598. Beretta, A., M. Cranage, and D. Zipeto, *Is Cross-Reactive Immunity Triggering COVID-19 Immunopathogenesis?* Frontiers in Immunology, 2020. **11**.
599. Zedan, H.T. and G.K. Nasrallah, *Is preexisting immunity to seasonal coronaviruses limited to cross-reactivity with SARS-CoV-2? A seroprevalence cross-sectional study in north-eastern France*. EBioMedicine, 2021. **71**.
600. Capoor, M.N., et al., *Is the "Common Cold" Our Greatest Ally in the Battle Against SARS-CoV-2?* Front Cell Infect Microbiol, 2020. **10**: p. 605334.
601. El-Qutob, D., et al., *Is there any effect of flu vaccine on the SARS-CoV-2 infected patients?* Vacunas, 2022. **23**(2): p. 71-76.
602. Jennewein, M.F., et al., *Isolation and characterization of cross-neutralizing coronavirus antibodies from COVID-19+ subjects*. Cell Reports, 2021. **36**(2).
603. Yi, C., et al., *Jigsaw puzzle of SARS-CoV-2 RBD evolution and immune escape*. Cellular and Molecular Immunology, 2022. **19**(7): p. 848-851.
604. Li, H., et al., *Key points of technical review for the registration of SARS-CoV-2 antigen/antibody tests*. Bioanalysis, 2021. **13**(2): p. 77-88.
605. Yi, C., et al., *Key residues of the receptor binding motif in the spike protein of SARS-CoV-2 that interact with ACE2 and neutralizing antibodies*. Cellular and Molecular Immunology, 2020. **17**(6): p. 621-630.
606. Mallapaty, S., *Kids and COVID: why young immune systems are still on top*. Nature, 2021. **597**(7875): p. 166-168.
607. Karlsson, A.C., M. Humbert, and M. Buggert, *The known unknowns of T cell immunity to COVID-19*. Science Immunology, 2020. **5**(53).
608. Dugas, M., et al., *Lack of antibodies against seasonal coronavirus OC43 nucleocapsid protein identifies patients at risk of critical COVID-19*. Journal of Clinical Virology, 2021. **139**: p. 104847.
609. Yang, R., et al., *Lack of antibody-mediated cross-protection between SARS-CoV-2 and SARS-CoV infections*. EBioMedicine, 2020. **58**.
610. Franchini, M., et al., *Lack of cross-reactivity between anti-A IgG isoagglutinins and anti-SARS-CoV-2 IgG antibodies*. Clinical Chemistry and Laboratory Medicine, 2021. **59**(7): p. E279-E281.
611. Pellegrino, G., et al., *Lack of cross-reactivity between rheumatoid factor IgM and anti-S1 receptor binding domain of SARS-CoV-2 IgM: a case-control study*. Clin Exp Rheumatol, 2022. **40**(7): p. 1417-1419.
612. Mehta, R., et al., *Lactate dehydrogenase and caspase activity in nasopharyngeal secretions are predictors of bronchiolitis severity*. Influenza Other Respir Viruses, 2014. **8**(6): p. 617-25.
613. Coutinho, A., et al., *Lessons of the month: Co-infection with SARS-CoV-2 and influenza B virus in a patient with community-acquired pneumonia*. Clinical Medicine, Journal of the Royal College of Physicians of London, 2020. **20**(6): p. E262-E263.
614. Zandi, M., E. Behboudi, and S. Soltani, *Letter to the Editor: Can the seasonal influenza vaccine for 2019/2020 have cross reactivity with some of the SARS-CoV-2 proteins?* International Journal of Infectious Diseases, 2021. **110**: p. 235-236.
615. Cohen, S.R., et al., *Leukocytoclastic vasculitis flare following the COVID-19 vaccine*. International Journal of Dermatology, 2021. **60**(8): p. 1032-1033.
616. Komadina, N., et al., *Likelihood of prior exposure to circulating influenza viruses resulting in cross-protection by CD8+ T cells against emergent H3N2v swine viruses infecting humans*. J Med Virol, 2022. **94**(2): p. 567-574.
617. Chen, J., et al., *A live attenuated virus-based intranasal COVID-19 vaccine provides rapid, prolonged, and broad protection against SARS-CoV-2*. Science Bulletin, 2022. **67**(13): p. 1372-1387.
618. Baluch, A., et al., *Long term immune responses to pandemic influenza A/H1N1 infection in solid organ transplant recipients*. PLoS One, 2011. **6**(12): p. e28627.
619. Balint, B., et al., *Long-term antibody-response monitoring following primary exposure to SARS-COV-2 and afterward mRNA COVID-19 vaccination: A case report*. Vojnosanitetski Pregled, 2021. **78**(3): p. 379-381.

620. Hollstein, M.M., et al., *Long-term effects of homologous and heterologous SARS-CoV-2 vaccination on humoral and cellular immune responses.* Allergy: European Journal of Allergy and Clinical Immunology, 2022. **77**(8): p. 2560-2564.
621. Lehners, N., et al., *Long-Term Shedding of Influenza Virus, Parainfluenza Virus, Respiratory Syncytial Virus and Nosocomial Epidemiology in Patients with Hematological Disorders.* PLoS One, 2016. **11**(2): p. e0148258.
622. Wirsching, S., et al., *Long-Term, CD4+ Memory T Cell Response to SARS-CoV-2.* Frontiers in Immunology, 2022. **13**.
623. Minervina, A.A., et al., *Longitudinal high-throughput TCR repertoire profiling reveals the dynamics of T-cell memory formation after mild COVID-19 infection.* Elife, 2021. **10**.
624. Legros, V., et al., *A longitudinal study of SARS-CoV-2-infected patients reveals a high correlation between neutralizing antibodies and COVID-19 severity.* Cellular and Molecular Immunology, 2021. **18**(2): p. 318-327.
625. Kawabuchi-Kurata, T., et al., *Longitudinal study on respiratory viral co-infections in the presence or absence of clinical manifestation in infants aged 0-2 years.* Jpn J Infect Dis, 2014. **67**(3): p. 216-20.
626. Bacher, P., et al., *Low-Avidity CD4+ T Cell Responses to SARS-CoV-2 in Unexposed Individuals and Humans with Severe COVID-19.* Immunity, 2020. **53**(6): p. 1258-1271.e5.
627. Mateus, J., et al., *Low-dose mRNA-1273 COVID-19 vaccine generates durable memory enhanced by cross-reactive T cells.* Science, 2021. **374**(6566).
628. Rolla, G., L. Brussino, and I. Badiu, *Maintaining safety with SARS-CoV-2 vaccines.* New England Journal of Medicine, 2021. **384**(10): p. E37.
629. Zhuang, Z., et al., *Mapping and role of T cell response in SARS-CoV-2-infected mice.* Journal of Experimental Medicine, 2021. **218**(4).
630. Rajan, M., et al., *Maternal and neonatal outcomes of COVID-19 co-infection in pregnant women with chronic hepatitis B virus infection: A prospective cohort study.* Int J Gynaecol Obstet, 2022. **158**(1): p. 221-222.
631. Rwezaura, H., et al., *Mathematical modeling and optimal control of SARS-CoV-2 and tuberculosis co-infection: a case study of Indonesia.* Modeling Earth Systems and Environment, 2022.
632. Sokal, A., et al., *Maturation and persistence of the anti-SARS-CoV-2 memory B cell response.* Cell, 2021. **184**(5): p. 1201-1213.e14.
633. Lee, M.S., et al., *Measuring antibody responses to a live attenuated influenza vaccine in children.* Pediatr Infect Dis J, 2004. **23**(9): p. 852-6.
634. Shafqat, A., et al., *Mechanistic Insights Into the Immune Pathophysiology of COVID-19; An In-Depth Review.* Frontiers in Immunology, 2022. **13**.
635. Kanjanapan, Y., et al., *Medical Oncology Group of Australia position statement: COVID-19 vaccination in patients with solid tumours.* Internal Medicine Journal, 2021. **51**(6): p. 955-959.
636. Tong, P., et al., *Memory B cell repertoire for recognition of evolving SARS-CoV-2 spike.* Cell, 2021. **184**(19): p. 4969-4980.e15.
637. Pušnik, J., et al., *Memory B cells targeting SARS-CoV-2 spike protein and their dependence on CD4+ T cell help.* Cell Reports, 2021. **35**(13).
638. He, B., et al., *The Metabolic Changes and Immune Profiles in Patients With COVID-19.* Frontiers in Immunology, 2020. **11**.
639. Hoque, M.N., et al., *Microbial co-infections in COVID-19: Associated microbiota and underlying mechanisms of pathogenesis.* Microbial Pathogenesis, 2021. **156**.
640. Chen, X., et al., *The microbial coinfection in COVID-19.* Applied Microbiology and Biotechnology, 2020. **104**(18): p. 7777-7785.
641. Denninger, V., et al., *Microfluidic Antibody Affinity Profiling Reveals the Role of Memory Reactivation and Cross-Reactivity in the Defense Against SARS-CoV-2.* ACS Infectious Diseases, 2022. **8**(4): p. 790-799.
642. Schneider, M.M., et al., *Microfluidic characterisation reveals broad range of SARS-CoV-2 antibody affinity in human plasma.* Life Science Alliance, 2022. **5**(2).
643. Adam, A., et al., *A modified porous silicon microparticle potentiates protective systemic and mucosal immunity for SARS-CoV-2 subunit vaccine.* Translational Research, 2022.
644. Routhu, N.K., et al., *A modified vaccinia Ankara vaccine expressing spike and nucleocapsid protects rhesus macaques against SARS-CoV-2 Delta infection.* Science Immunology, 2022. **7**(72).
645. Lin, G.L., et al., *Molecular epidemiology and clinical features of adenovirus infection in Taiwanese children, 2014.* J Microbiol Immunol Infect, 2019. **52**(2): p. 215-224.
646. Kanduc, D. and Y. Shoenfeld, *Molecular mimicry between SARS-CoV-2 spike glycoprotein and mammalian proteomes: implications for the vaccine.* Immunologic Research, 2020. **68**(5): p. 310-313.
647. Lee, C.Y., et al., *Molecular viral epidemiology and clinical characterization of acute febrile respiratory infections in hospitalized children in Taiwan.* J Med Virol, 2015. **87**(11): p. 1860-6.
648. Fischer, N., et al., *Monitoring of human coronaviruses in Belgian primary care and hospitals, 2015-20: a surveillance study.* Lancet Microbe, 2021. **2**(3): p. e105-e114.

649. Zheng, Z., et al., *Monoclonal antibodies for the S2 subunit of spike of SARS-CoV-1 cross-react with the newly-emerged SARSCoV-2.* Eurosurveillance, 2020. **25**(28): p. 19-28.
650. Abduljaleel, Z., et al., *Monoclonal antibody designed for SARS-nCoV-2 spike protein of receptor binding domain on antigenic targeted epitopes for inhibition to prevent viral entry.* Molecular Diversity, 2022.
651. Wang, P., et al., *A monoclonal antibody that neutralizes SARS-CoV-2 variants, SARS-CoV, and other sarbecoviruses.* Emerging Microbes and Infections, 2022. **11**(1): p. 147-157.
652. de Sousa, E., et al., *Mortality in COVID-19 disease patients: Correlating the association of major histocompatibility complex (MHC) with severe acute respiratory syndrome 2 (SARS-CoV-2) variants.* International Journal of Infectious Diseases, 2020. **98**: p. 454-459.
653. Wang, H., et al., *mRNA based vaccines provide broad protection against different SARS-CoV-2 variants of concern.* Emerging Microbes and Infections, 2022. **11**(1): p. 1550-1553.
654. Koo, G., et al., *mRNA COVID-19 vaccine safety in patients with previous immediate hypersensitivity to pegaspargase.* Journal of Allergy and Clinical Immunology: In Practice, 2022. **10**(1): p. 322-325.
655. Giannotta, G. and N. Giannotta, *Mrna covid-19 vaccines and long-lived plasma cells: A complicated relationship.* Vaccines, 2021. **9**(12).
656. Gagne, M., et al., *mRNA-1273 or mRNA-Omicron boost in vaccinated macaques elicits similar B cell expansion, neutralizing responses, and protection from Omicron.* Cell, 2022. **185**(9): p. 1556-1571.e18.
657. Tejedor Vaquero, S., et al., *The mRNA-1273 Vaccine Induces Cross-Variant Antibody Responses to SARS-CoV-2 With Distinct Profiles in Individuals With or Without Pre-Existing Immunity.* Frontiers in Immunology, 2021. **12**.
658. Kaplonek, P., et al., *mRNA-1273 vaccine-induced antibodies maintain Fc effector functions across SARS-CoV-2 variants of concern.* Immunity, 2022. **55**(2): p. 355-365.e4.
659. Garcia-Beltran, W.F., et al., *mRNA-based COVID-19 vaccine boosters induce neutralizing immunity against SARS-CoV-2 Omicron variant.* Cell, 2022. **185**(3): p. 457-466.e4.
660. Pierantoni, A., et al., *Mucosal delivery of a vectored RSV vaccine is safe and elicits protective immunity in rodents and nonhuman primates.* Molecular Therapy - Methods and Clinical Development, 2015. **2**: p. 15018.
661. Singh, S. and I.A. Qureshi, *Multi-epitope vaccine against SARS-CoV-2 applying immunoinformatics and molecular dynamics simulation approaches.* Journal of Biomolecular Structure and Dynamics, 2022. **40**(7): p. 2917-2933.
662. Russo, G., et al., *A multi-step and multi-scale bioinformatic protocol to investigate potential SARS-CoV-2 vaccine targets.* Briefings in Bioinformatics, 2022. **23**(1).
663. Akbay, B., et al., *Multi-subunit sars-cov-2 vaccine design using evolutionarily conserved t-and b-cell epitopes.* Vaccines, 2021. **9**(7).
664. Abela, I.A., et al., *Multifactorial seroprofiling dissects the contribution of pre-existing human coronaviruses responses to SARS-CoV-2 immunity.* Nature Communications, 2021. **12**(1).
665. Stempel, H.E., et al., *Multiple viral respiratory pathogens in children with bronchiolitis.* Acta Paediatr, 2009. **98**(1): p. 123-6.
666. Fang, Z.F., et al., *Multiplexed analysis of circulating IgA antibodies for SARS-CoV-2 and common respiratory pathogens in COVID-19 patients.* Journal of Medical Virology, 2021. **93**(5): p. 3257-3260.
667. Peng, L., et al., *Multiplexed LNP-mRNA vaccination against pathogenic coronavirus species.* Cell Reports, 2022. **40**(5).
668. Kim, S.A., et al., *A Multivalent Vaccine Based on Ferritin Nanocage Elicits Potent Protective Immune Responses against SARS-CoV-2 Mutations.* International Journal of Molecular Sciences, 2022. **23**(11).
669. Huang, J., et al., *Nasal Nanovaccines for SARS-CoV-2 to Address COVID-19.* Vaccines, 2022. **10**(3).
670. Hagemann, K., et al., *Natural killer cell-mediated ADCC in SARS-CoV-2-infected individuals and vaccine recipients.* European Journal of Immunology, 2022. **52**(8): p. 1297-1307.
671. Wu, N.C., et al., *A natural mutation between SARS-CoV-2 and SARS-CoV determines neutralization by a cross-reactive antibody.* PLoS Pathogens, 2020. **16**(12).
672. Netea, M.G., et al., *Natural resistance against infections: focus on COVID-19.* Trends in Immunology, 2022. **43**(2): p. 106-116.
673. Poland, G.A., I.G. Ovsyannikova, and R.B. Kennedy, *The need for broadly protective COVID-19 vaccines: Beyond S-only approaches.* Vaccine, 2021. **39**(31): p. 4239-4241.
674. Kobayashi, K., M. Tachibana, and Y. Tsutsumi, *Neglected roles of IgG Fc-binding protein secreted from airway mucin-producing cells in protecting against SARS-CoV-2 infection.* Innate Immunity, 2021. **27**(6): p. 423-436.
675. Lavezzo, E., et al., *Neutralising reactivity against SARS-CoV-2 Delta and Omicron variants by vaccination and infection history.* Genome Medicine, 2022. **14**(1).
676. Sun, X., et al., *Neutralization mechanism of a human antibody with pan-coronavirus reactivity including SARS-CoV-2.* Nature Microbiology, 2022. **7**(7): p. 1063-1074.
677. Lei, C., et al., *Neutralization of SARS-CoV-2 spike pseudotyped virus by recombinant ACE2-Ig.* Nature Communications, 2020. **11**(1).

678. Sapkal, G., et al., *Neutralization of VUI B.1.1.28 P2 variant with sera of COVID-19 recovered cases and recipients of Covaxin an inactivated COVID-19 vaccine*. Journal of Travel Medicine, 2021. **28**(7).
679. Lai, G.C., et al., *Neutralization or enhancement of SARS-CoV-2 infection by a monoclonal antibody targeting a specific epitope in the spike receptor-binding domain*. Antiviral Research, 2022. **200**.
680. Rössler, A., et al., *Neutralization Profile after Recovery from SARS-CoV-2 Omicron Infection*. New England Journal of Medicine, 2022. **386**(18): p. 1764-1766.
681. Choi, J.Y., et al., *Neutralizing Activity Against SARS-CoV-2 Delta and Omicron Variants Following a Third BNT162b2 Booster Dose According to Three Homologous or Heterologous COVID-19 Vaccination Schedules*. Frontiers in Cellular and Infection Microbiology, 2022. **12**.
682. Yu, X., et al., *Neutralizing activity of BBIBP-CorV vaccine-elicited sera against Beta, Delta and other SARS-CoV-2 variants of concern*. Nature Communications, 2022. **13**(1).
683. Cohen, S.A., C. Kellogg, and O. Equils, *Neutralizing and cross-reacting antibodies: implications for immunotherapy and SARS-CoV-2 vaccine development*. Human Vaccines and Immunotherapeutics, 2020: p. 1-4.
684. Miller, M.S., et al., *Neutralizing antibodies against previously encountered influenza virus strains increase over time: a longitudinal analysis*. Sci Transl Med, 2013. **5**(198): p. 198ra107.
685. Huang, Y., et al., *Neutralizing antibodies against SARS-CoV-2: Current understanding, challenge and perspective*. Antibody Therapeutics, 2020. **3**(4): p. 285-299.
686. Jacobs, J.J.L., *Neutralizing antibodies mediate virus-immune pathology of COVID-19*. Medical Hypotheses, 2020. **143**.
687. Haveri, A., et al., *Neutralizing antibodies to SARS-CoV-2 Omicron variant after third mRNA vaccination in health care workers and elderly subjects*. European Journal of Immunology, 2022. **52**(5): p. 816-824.
688. Newman, J., et al., *Neutralizing antibody activity against 21 SARS-CoV-2 variants in older adults vaccinated with BNT162b2*. Nature Microbiology, 2022. **7**(8): p. 1180-1188.
689. Montesinos, I., et al., *Neutralizing antibody responses following natural SARS-CoV-2 infection: Dynamics and correlation with commercial serologic tests*. Journal of Clinical Virology, 2021. **144**.
690. Deshpande, G.R., et al., *Neutralizing antibody responses to SARS-CoV-2 in COVID-19 patients*. Indian Journal of Medical Research, 2020. **152**(1): p. 82-87.
691. Saunders, K.O., et al., *Neutralizing antibody vaccine for pandemic and pre-emergent coronaviruses*. Nature, 2021. **594**(7864): p. 553-559.
692. Kim, Y.J., et al., *Neutralizing human antibodies against severe acute respiratory syndrome coronavirus 2 isolated from a human synthetic fab phage display library*. International Journal of Molecular Sciences, 2021. **22**(4): p. 1-18.
693. Servellita, V., et al., *Neutralizing immunity in vaccine breakthrough infections from the SARS-CoV-2 Omicron and Delta variants*. Cell, 2022. **185**(9): p. 1539-1548.e5.
694. Maffey, A.F., et al., *[New respiratory viruses in children 2 months to 3 years old with recurrent wheeze]*. Arch Argent Pediatr, 2008. **106**(4): p. 302-9.
695. Gloeckner, S., et al., *Newborns' passive humoral SARS-CoV-2 immunity following heterologous vaccination of the mother during pregnancy*. American Journal of Obstetrics and Gynecology, 2022. **226**(2): p. 261-262.
696. Schwaiger, J., et al., *No SARS-CoV-2 neutralization by intravenous immunoglobulins produced from plasma collected before the 2020 pandemic*. Journal of Infectious Diseases, 2020. **222**(12): p. 1960-1964.
697. Murugavelu, P., et al., *Non-neutralizing SARS CoV-2 directed polyclonal antibodies demonstrate cross-reactivity with the HA glycans of influenza virus*. Int Immunopharmacol, 2021. **99**: p. 108020.
698. Sonabend, R., et al., *Non-pharmaceutical interventions, vaccination, and the SARS-CoV-2 delta variant in England: a mathematical modelling study*. The Lancet, 2021. **398**(10313): p. 1825-1835.
699. Abouelkhair, M.A., *Non-SARS-CoV-2 genome sequences identified in clinical samples from COVID-19 infected patients: Evidence for co-infections*. PeerJ, 2020. **8**.
700. Giwa, A.L., A. Desai, and A. Duca, *Novel 2019 coronavirus SARS-CoV-2 (COVID-19): An updated overview for emergency clinicians*. Emergency medicine practice, 2020. **22**(5): p. 1-28.
701. Kohler, H. and P. Nara, *A Novel Hypothesis for Original Antigenic Sin in the Severe Disease of SARS-CoV-2 Infection*. Monoclonal Antibodies in Immunodiagnosis and Immunotherapy, 2020. **39**(4): p. 107-111.
702. Francis, J.R., et al., *An observational study of febrile seizures: the importance of viral infection and immunization*. BMC Pediatr, 2016. **16**(1): p. 202.
703. Dan, J., et al., *Observations and Perspectives on Adaptive Immunity to Severe Acute Respiratory Syndrome Coronavirus 2 (SARS-CoV-2)*. Clinical infectious diseases : an official publication of the Infectious Diseases Society of America, 2022. **75**(1): p. S24-S29.
704. Michelitsch, A., et al., *Occurrence of antibodies against SARS-CoV-2 in the domestic cat population of Germany*. Vaccines, 2020. **8**(4): p. 1-10.
705. Chakrabarti, S.S., et al., *Of cross-immunity, herd immunity and country-specific plans: Experiences from COVID-19 in India*. Aging and Disease, 2020. **11**(6): p. 1339-1344.
706. Nicoli, F., D. Paudel, and M.T. Solis-Soto, *Old and new coronaviruses in the elderly*. Aging, 2021. **13**(9): p. 12295-12296.

707. Chumakov, K., et al., *Old vaccines for new infections: Exploiting innate immunity to control COVID-19 and prevent future pandemics*. Proceedings of the National Academy of Sciences of the United States of America, 2021. **118**(21).
708. Saletti, G., et al., *Older adults lack SARS CoV-2 cross-reactive T lymphocytes directed to human coronaviruses OC43 and NL63*. Scientific Reports, 2020. **10**(1).
709. Emmelot, M.E., et al., *Omicron BA.1 Mutations in SARS-CoV-2 Spike Lead to Reduced T-Cell Response in Vaccinated and Convalescent Individuals*. Viruses, 2022. **14**(7).
710. Jia, L., et al., *Omicron Booster in Ancestral Strain Vaccinated Mice Augments Protective Immunities Against Both Delta and Omicron Variants*. Frontiers in Immunology, 2022. **13**.
711. Khan, K., et al., *Omicron infection enhances Delta antibody immunity in vaccinated persons*. Nature, 2022. **607**(7918): p. 356-359.
712. Anichini, G., et al., *Omicron Infection Evokes Cross-Protection against SARS-CoV-2 Variants in Vaccinees*. Vaccines, 2022. **10**(5).
713. Burhan, E. and R.A. Rachmadi, *Omicron surge and the future of COVID-19 vaccinations*. Medical Journal of Indonesia, 2022. **31**(1): p. 80-84.
714. Fang, Z., et al., *Omicron-specific mRNA vaccination alone and as a heterologous booster against SARS-CoV-2*. Nature Communications, 2022. **13**(1).
715. Kanduc, D. and Y. Shoenfeld, *On the molecular determinants of the SARS-CoV-2 attack*. Clinical Immunology, 2020. **215**.
716. Gu, M., et al., *One dose of COVID-19 nanoparticle vaccine REVC-128 protects against SARS-CoV-2 challenge at two weeks post-immunization*. Emerging Microbes and Infections, 2021. **10**(1): p. 2016-2029.
717. To, K.K., et al., *Ongoing transmission of avian influenza A viruses in Hong Kong despite very comprehensive poultry control measures: A prospective seroepidemiology study*. J Infect, 2016. **72**(2): p. 207-13.
718. Malave Sanchez, M., et al., *Oral Polio Vaccine to Protect against COVID-19: Out of the Box Strategies?* Open Forum Infectious Diseases, 2021. **8**(8).
719. Pitcovski, J., et al., *Oral subunit SARS-CoV-2 vaccine induces systemic neutralizing IgG, IgA and cellular immune responses and can boost neutralizing antibody responses primed by an injected vaccine*. Vaccine, 2022. **40**(8): p. 1098-1107.
720. Noori, M., S.A. Nejadghaderi, and N. Rezaei, *Original antigenic sin: A potential threat beyond the development of booster vaccination against novel SARS-CoV-2 variants*. Infection Control and Hospital Epidemiology, 2022. **43**(8): p. 1091-1092.
721. Gillot, C., et al., *An original elisa-based multiplex method for the simultaneous detection of 5 sars-cov-2 igg antibodies directed against different antigens*. Journal of Clinical Medicine, 2020. **9**(11): p. 1-13.
722. Borgsteede, S.D., T.H. Geersing, and Ž. Tempels-Pavlica, *Other excipients than PEG might cause serious hypersensitivity reactions in COVID-19 vaccines*. Allergy: European Journal of Allergy and Clinical Immunology, 2021. **76**(6): p. 1941-1942.
723. Shang, W., et al., *The outbreak of SARS-CoV-2 pneumonia calls for viral vaccines*. npj Vaccines, 2020. **5**(1).
724. Rajatonirina, S., et al., *Outcome risk factors during respiratory infections in a paediatric ward in Antananarivo, Madagascar 2010-2012*. PLoS One, 2013. **8**(9): p. e72839.
725. Shah, V.K., et al., *Overview of Immune Response During SARS-CoV-2 Infection: Lessons From the Past*. Frontiers in Immunology, 2020. **11**.
726. Suzuki, Y., *Overview of the COVID-19 Pandemic in Japan: Public Health Perspectives in the first half of 2020*. Keio Journal of Medicine, 2021. **70**(4): p. 73-81.
727. Cordero, E., et al., *Pandemic influenza A(H1N1) virus infection in solid organ transplant recipients: impact of viral and non-viral co-infection*. Clin Microbiol Infect, 2012. **18**(1): p. 67-73.
728. Marchiori, R., et al., *Pandemic influenza A/H1N1: comparative analysis of microscopic lung histopathological findings*. Einstein (Sao Paulo), 2012. **10**(3): p. 306-11.
729. Ogunbayo, A.E., et al., *Pathogen Profile of Children Hospitalised with Severe Acute Respiratory Infections during COVID-19 Pandemic in the Free State Province, South Africa*. International journal of environmental research and public health, 2022. **19**(16).
730. Ji, Y.H., et al., *Pathogenic analysis of suspected COVID-19 patients in a SARS-CoV-2 non-epidemic area of China*. European Review for Medical and Pharmacological Sciences, 2020. **24**(17): p. 9196-9201.
731. Brecher, S.M., et al., *Patients with common cold coronaviruses tested negative for igg antibody to sars-cov-2*. Journal of Clinical Microbiology, 2020. **58**(8).
732. Tonkin-Hill, G., et al., *Patterns of within-host genetic diversity in SARS-COV-2*. eLife, 2021. **10**.
733. Wang, Z. and J. Sun, *PD-1hi exhausted-like CD8+resident memory T cells balance immunity and fibrotic sequela*. European Journal of Immunology, 2019. **49**: p. 1069.
734. Somogyi, E., et al., *A Peptide Vaccine Candidate Tailored to Individuals' Genetics Mimics the Multi-Targeted T Cell Immunity of COVID-19 Convalescent Subjects*. Frontiers in Genetics, 2021. **12**.
735. Iyer, A.S., et al., *Persistence and decay of human antibody responses to the receptor binding domain of SARS-CoV-2 spike protein in COVID-19 patients*. Sci Immunol, 2020. **5**(52).
736. Tanunliong, G., et al., *Persistence of Anti-SARS-CoV-2 Antibodies in Long Term Care Residents Over Seven Months After Two COVID-19 Outbreaks*. Frontiers in Immunology, 2021. **12**.

737. Duysburgh, E., et al., *Persistence of IgG response to SARS-CoV-2*. The Lancet Infectious Diseases, 2021. **21**(2): p. 163-164.
738. Fang, H., et al., *Persistent covid-19 symptoms minimally impact the development of sars-cov-2-specific t cell immunity*. Viruses, 2021. **13**(5).
739. Fonseca, M.H.G., et al., *Persistently positive SARS-CoV-2-specific IgM during 1-year follow-up*. Journal of Medical Virology, 2022. **94**(9): p. 4037-4039.
740. Arvin, A.M., et al., *A perspective on potential antibody-dependent enhancement of SARS-CoV-2*. Nature, 2020. **584**(7821): p. 353-363.
741. Weiskopf, D., et al., *Phenotype and kinetics of SARS-CoV-2-specific T cells in COVID-19 patients with acute respiratory distress syndrome*. Sci Immunol, 2020. **5**(48).
742. Jung, M.K. and E.C. Shin, *Phenotypes and functions of sars-cov-2-reactive t cells*. Molecules and Cells, 2021. **44**(6): p. 401-407.
743. Nishinarita, S., S. Sawada, and T. Horie, *Phosphorylcholine antibodies in pulmonary infection*. Med Microbiol Immunol, 1990. **179**(4): p. 205-14.
744. Bekçibaşı, M., *Physicians' Approaches to Prophylaxis and Vaccination During the COVID-19 Pandemic: A Cross-sectional Survey Study from Turkey*. Klimik Dergisi, 2022. **35**(1): p. 21-25.
745. Domenech de Cellès, M., et al., *The pitfalls of inferring virus-virus interactions from co-detection prevalence data: application to influenza and SARS-CoV-2*. Proceedings of the Royal Society B: Biological Sciences, 2022. **289**(1966): p. 20212358.
746. Samransamruajkit, R., et al., *Plasma endothelin-1 in infants and young children with acute bronchiolitis and viral pneumonia*. Asian Pac J Allergy Immunol, 2002. **20**(4): p. 229-34.
747. Qiu, S., et al., *Pneumonia Patients Caused by Co-infection With SARS-CoV-2 and Human Adenovirus in China*. Frontiers in Medicine, 2021. **8**.
748. Comunale, B.A., et al., *Poliovirus Vaccination Induces a Humoral Immune Response That Cross Reacts With SARS-CoV-2*. Frontiers in Medicine, 2021. **8**.
749. Sellaturay, P., et al., *The Polysorbate containing AstraZeneca COVID-19 vaccine is tolerated by polyethylene glycol (PEG) allergic patients*. Clinical and Experimental Allergy, 2022. **52**(1): p. 12-17.
750. Wehenkel, C., *Positive association between COVID-19 deaths and influenza vaccination rates in elderly people worldwide*. PeerJ, 2020. **8**.
751. Wu, D., et al., *Positive effects of COVID-19 control measures on influenza prevention*. International Journal of Infectious Diseases, 2020. **95**: p. 345-346.
752. Chan, C.P., et al., *Positive impact of measures against COVID-19 on reducing influenza in the Northern Hemisphere*. Journal of Travel Medicine, 2020. **27**(8).
753. Brotons, P., et al., *The positive rhinovirus/enterovirus detection and sars-cov-2 persistence beyond the acute infection phase: An intra-household surveillance study*. Viruses, 2021. **13**(8).
754. Nutalai, R., et al., *Potent cross-reactive antibodies following Omicron breakthrough in vaccinees*. Cell, 2022. **185**(12): p. 2116-2131.e18.
755. Halfmann, P.J., et al., *Potent neutralization of SARS-CoV-2 including variants of concern by vaccines presenting the receptor-binding domain multivalently from nanoscaffolds*. Bioengineering and Translational Medicine, 2021. **6**(3).
756. Rouet, R., et al., *Potent SARS-CoV-2 binding and neutralization through maturation of iconic SARS-CoV-1 antibodies*. mAbs, 2021. **13**(1).
757. Huang, K.Y., et al., *A Potent Virus-Specific Antibody-Secreting Cell Response to Acute Enterovirus 71 Infection in Children*. J Infect Dis, 2015. **212**(5): p. 808-17.
758. Lee, C.H., et al., *Potential CD8+ T Cell Cross-Reactivity Against SARS-CoV-2 Conferred by Other Coronavirus Strains*. Frontiers in Immunology, 2020. **11**.
759. Reche, P.A., *Potential Cross-Reactive Immunity to SARS-CoV-2 From Common Human Pathogens and Vaccines*. Frontiers in Immunology, 2020. **11**: p. 586984.
760. Pinotti, F., et al., *Potential impact of individual exposure histories to endemic human coronaviruses on age-dependent severity of COVID-19*. BMC Med, 2021. **19**(1): p. 19.
761. Rabets, A., et al., *The Potential of Developing Pan-Coronaviral Antibodies to Spike Peptides in Convalescent COVID-19 Patients*. Archivum Immunologiae et Therapiae Experimentalis, 2021. **69**(1).
762. Zost, S.J., et al., *Potently neutralizing and protective human antibodies against SARS-CoV-2*. Nature, 2020. **584**(7821): p. 443-449.
763. Gopaul, R., et al., *Practical Diagnostic Accuracy of Nasopharyngeal Swab Testing for Novel Coronavirus Disease 2019 (COVID-19)*. West J Emerg Med, 2020. **21**(6): p. 1-4.
764. Miyara, M., et al., *Pre-COVID-19 Immunity to Common Cold Human Coronaviruses Induces a Recall-Type IgG Response to SARS-CoV-2 Antigens Without Cross-Neutralisation*. Frontiers in Immunology, 2022. **13**.
765. Wang, J., et al., *Pre-Existing Cross-Reactive Antibody Responses Do Not Significantly Impact Inactivated COVID-19 Vaccine-Induced Neutralization*. Frontiers in Immunology, 2021. **12**.
766. Lin, C.Y., et al., *Pre-existing humoral immunity to human common cold coronaviruses negatively impacts the protective SARS-CoV-2 antibody response*. Cell Host Microbe, 2022. **30**(1): p. 83-96.e4.

767. Sette, A. and S. Crotty, *Pre-existing immunity to SARS-CoV-2: the knowns and unknowns*. Nature Reviews Immunology, 2020. **20**(8): p. 457-458.
768. Swadling, L., et al., *Pre-existing polymerase-specific T cells expand in abortive seronegative SARS-CoV-2*. Nature, 2022. **601**(7891): p. 110-117.
769. Casado, J.L., et al., *Pre-existing T cell immunity determines the frequency and magnitude of cellular immune response to two doses of mRNA vaccine against SARS-CoV-2*. Vaccine: X, 2022. **11**.
770. Tan, C.C.S., et al., *Pre-existing T cell-mediated cross-reactivity to SARS-CoV-2 cannot solely be explained by prior exposure to endemic human coronaviruses*. Infection, Genetics and Evolution, 2021. **95**.
771. Echeverría, G., et al., *Pre-existing T-cell immunity to SARS-CoV-2 in unexposed healthy controls in Ecuador, as detected with a COVID-19 Interferon-Gamma Release Assay*. International Journal of Infectious Diseases, 2021. **105**: p. 21-25.
772. Aparicio, B., et al., *Preclinical evaluation of a synthetic peptide vaccine against SARS-CoV-2 inducing multiepitopic and cross-reactive humoral neutralizing and cellular CD4 and CD8 responses*. Emerging Microbes and Infections, 2021. **10**(1): p. 1931-1946.
773. Banerjee, A., et al., *Predicting the recombination potential of severe acute respiratory syndrome coronavirus 2 and Middle East respiratory syndrome coronavirus*. Journal of General Virology, 2021. **101**(12): p. 1251-1260.
774. López, D., *Prediction of Conserved HLA Class I and Class II Epitopes from SARS-CoV-2 Licensed Vaccines Supports T-Cell Cross-Protection against SARS-CoV-1*. Biomedicines, 2022. **10**(7).
775. Alshanbari, H.M., et al., *Prediction of COVID-19 severity from clinical and biochemical markers: a single-center study from Saudi Arabia*. Eur Rev Med Pharmacol Sci, 2022. **26**(7): p. 2592-2601.
776. Valkenburg, S.A., et al., *Preexisting Antibody-Dependent Cellular Cytotoxicity-Activating Antibody Responses Are Stable Longitudinally and Cross-reactive Responses Are Not Boosted by Recent Influenza Exposure*. J Infect Dis, 2016. **214**(8): p. 1159-63.
777. Yamashita, T., et al., *Preexisting Humoral Immunity Cross-Reacting with SARS-CoV-2 Might Prevent Death Due to COVID-19 in Critical Patients*. Journal of Clinical Medicine, 2022. **11**(13).
778. Oshiro, S., et al., *Presence of antibodies against SARS-CoV-2 spike protein in bovine whey IgG enriched fraction*. International Dairy Journal, 2021. **117**.
779. Richardson, S., et al., *Presenting Characteristics, Comorbidities, and Outcomes among 5700 Patients Hospitalized with COVID-19 in the New York City Area*. JAMA - Journal of the American Medical Association, 2020. **323**(20): p. 2052-2059.
780. García-García, M.L., et al., *Prevalence and clinical characteristics of human metapneumovirus infections in hospitalized infants in Spain*. Pediatr Pulmonol, 2006. **41**(9): p. 863-71.
781. Tatarelli, P., et al., *Prevalence and clinical impact of VIral Respiratory tract infections in patients hospitalized for Community-Acquired Pneumonia: the VIRCAP study*. Intern Emerg Med, 2020. **15**(4): p. 645-654.
782. Meena, J.P., et al., *Prevalence and clinical outcome of respiratory viral infections among children with cancer and febrile neutropenia*. Pediatr Hematol Oncol, 2019. **36**(6): p. 330-343.
783. Uribe-Gutiérrez, G., et al., *Prevalence and genotypes of the adenovirus infection as well detection of co-infection with bocavirus in Mexican immunosuppressed and non-immunosuppressed children with pneumonia*. Clin Lab, 2014. **60**(8): p. 1277-85.
784. Musuuza, J.S., et al., *Prevalence and outcomes of co-infection and superinfection with SARS-CoV-2 and other pathogens: A systematic review and metaanalysis*. PLoS ONE, 2021. **16**(5 May).
785. Ramaekers, K., et al., *Prevalence and seasonality of six respiratory viruses during five consecutive epidemic seasons in Belgium*. J Clin Virol, 2017. **94**: p. 72-78.
786. Karaba, S.M., et al., *Prevalence of co-infection at the time of hospital admission in COVID-19 Patients, A multicenter study*. Open Forum Infectious Diseases, 2021. **8**(1).
787. Meskill, S.D., et al., *Prevalence of co-infection between respiratory syncytial virus and influenza in children*. Am J Emerg Med, 2017. **35**(3): p. 495-498.
788. Peci, A., et al., *Prevalence of co-infections with respiratory viruses in individuals investigated for sars-cov-2 in Ontario, Canada*. Viruses, 2021. **13**(1).
789. Celik, K., et al., *Prevalence of respiratory pathogens during two consecutive respiratory syncytial virus seasons at a tertiary medical care center*. Arch Argent Pediatr, 2019. **117**(4): p. e356-e362.
790. Mahajan, N.N., et al., *Prevalence, Clinical Presentations and Treatment Outcomes of COVID-19 among Healthcare Workers at a Dedicated Hospital in India*. The Journal of the Association of Physicians of India, 2020. **68**(12): p. 16-21.
791. Focosi, D., et al., *Previous humoral immunity to the endemic seasonal alphacoronaviruses NL63 and 229E is associated with worse clinical outcome in COVID-19 and suggests original antigenic sin*. Life, 2021. **11**(4).
792. Trinité, B., et al., *Previous sars-cov-2 infection increases b.1.1.7 cross-neutralization by vaccinated individuals*. Viruses, 2021. **13**(6).
793. Cox, G., et al., *Priming With Rhinovirus Protects Mice Against a Lethal Pulmonary Coronavirus Infection*. Frontiers in Immunology, 2022. **13**.

794. Casadevall, A., L.A. Pirofski, and M.J. Joyner, *The principles of antibody therapy for infectious diseases with relevance for covid-19.* mBio, 2021. **12**(2): p. 1-13.
795. Aran, D., et al., *Prior presumed coronavirus infection reduces COVID-19 risk: A cohort study.* J Infect, 2020. **81**(6): p. 923-930.
796. Reynolds, C.J., et al., *Prior SARS-CoV-2 infection rescues B and T cell responses to variants after first vaccine dose.* Science, 2021. **372**(6549): p. 1418-1423.
797. Guo, L., et al., *Profiling early humoral response to diagnose novel coronavirus disease (COVID-19).* Clinical Infectious Diseases, 2020. **71**(15): p. 778-785.
798. Sakharkar, M., et al., *Prolonged evolution of the human B cell response to SARS-CoV-2 infection.* Sci Immunol, 2021. **6**(56).
799. Chen, W., *Promise and challenges in the development of COVID-19 vaccines.* Human Vaccines and Immunotherapeutics, 2020. **16**(11): p. 2604-2608.
800. Mattoo, S.U.S. and J. Myoung, *A Promising Vaccination Strategy against COVID-19 on the Horizon: Heterologous Immunization.* Journal of Microbiology and Biotechnology, 2021. **31**(12): p. 1601-1614.
801. Jackson-Thompson, B.M., et al., *Prospective Assessment of SARS-CoV-2 Seroconversion (PASS) study: an observational cohort study of SARS-CoV-2 infection and vaccination in healthcare workers.* BMC Infect Dis, 2021. **21**(1): p. 544.
802. Krueger, W.S., et al., *Prospective study of avian influenza virus infections among rural Thai villagers.* PLoS One, 2013. **8**(8): p. e72196.
803. Sabharwal, S., et al., *A prospective study of prevalence of respiratory viruses causing acute respiratory infection in pediatric in-patients during pre- COVID times.* Indian J Med Microbiol, 2021. **39**(4): p. 429-433.
804. Coman, A., et al., *A prospective study of Romanian agriculture workers for zoonotic influenza infections.* PLoS One, 2014. **9**(5): p. e98248.
805. Ortiz-Lana, N., et al., *[A prospective study to assess the burden of influenza-related hospitalizations and emergency department visits among children in Bilbao, Spain (2010-2011)].* An Pediatr (Barc), 2017. **87**(6): p. 311-319.
806. Mysore, V., et al., *Protective heterologous T cell immunity in COVID-19 induced by the trivalent MMR and Tdap vaccine antigens.* Med, 2021. **2**(9): p. 1050-1071.e7.
807. Choudhary, O.P. and I. Singh, *Protective immunity against COVID-19: Unravelling the evidences for humoral vs. cellular components.* Travel Medicine and Infectious Disease, 2021. **39**.
808. Pavot, V., et al., *Protein-based SARS-CoV-2 spike vaccine booster increases cross-neutralization against SARS-CoV-2 variants of concern in non-human primates.* Nature Communications, 2022. **13**(1).
809. Rajsri, K.S., et al., *A Rapid and Sensitive Microfluidics-Based Tool for Seroprevalence Immunity Assessment of COVID-19 and Vaccination-Induced Humoral Antibody Response at the Point of Care.* Biosensors, 2022. **12**(8).
810. Yuan, T.Z., et al., *Rapid discovery of diverse neutralizing SARS-CoV-2 antibodies from large-scale synthetic phage libraries.* mAbs, 2022. **14**(1).
811. Zost, S.J., et al., *Rapid isolation and profiling of a diverse panel of human monoclonal antibodies targeting the SARS-CoV-2 spike protein.* Nature Medicine, 2020. **26**(9): p. 1422-1427.
812. Kim, D., et al., *Rates of Co-infection between SARS-CoV-2 and Other Respiratory Pathogens.* JAMA - Journal of the American Medical Association, 2020. **323**(20): p. 2085-2086.
813. Pan, X., et al., *RBD-homodimer, a COVID-19 subunit vaccine candidate, elicits immunogenicity and protection in rodents and nonhuman primates.* Cell Discovery, 2021. **7**(1).
814. Zeng, G., *Re: Vaccine effectiveness of ChAdOx1 nCoV-19 against COVID-19 in a socially vulnerable community in Rio de Janeiro, Brazil by Ranzani et al.* Clinical Microbiology and Infection, 2022. **28**(8): p. 1165.
815. Vojdani, A., et al., *Reaction of SARS-CoV-2 antibodies with other pathogens, vaccines, and food antigens.* Frontiers in Immunology, 2022. **13**.
816. Sibbel, S., et al., *Real-World Effectiveness and Immunogenicity of BNT162b2 and mRNA-1273 SARS-CoV-2 Vaccines in Patients on Hemodialysis.* Journal of the American Society of Nephrology, 2022. **33**(1): p. 49-57.
817. Muehling, L., et al., *Recent Common Cold Does Not Reliably Protect from Re-Infection and Fails to Cross-Protect Fifteen Weeks Later, Despite Involvement of Cross-Reactive T Cells.* Journal of Allergy and Clinical Immunology, 2021. **147**(2): p. AB164.
818. Zhu, Y., J. Li, and Z. Pang, *Recent insights for the emerging COVID-19: Drug discovery, therapeutic options and vaccine development.* Asian Journal of Pharmaceutical Sciences, 2021. **16**(1): p. 4-23.
819. Shi, J., et al., *Receptor-binding domain proteins of sars-cov-2 variants elicited robust antibody responses cross-reacting with wild-type and mutant viruses in mice.* Vaccines, 2021. **9**(12).
820. Trigueros, M., et al., *Reduced humoral response 3 months following BNT162b2 vaccination in SARS-CoV-2 uninfected residents of long-term care facilities.* Age and Ageing, 2022. **51**(5).
821. Sung, M., et al., *Regional and annual patterns in respiratory virus co-infection etiologies and antibiotic prescriptions for pediatric mycoplasma pneumoniae pneumonia.* Eur Rev Med Pharmacol Sci, 2022. **26**(16): p. 5844-5856.

822. Galipeau, Y., et al., *Relative Ratios of Human Seasonal Coronavirus Antibodies Predict the Efficiency of Cross-Neutralization of SARS-CoV-2 Spike Binding to ACE2*. EBioMedicine, 2021. **74**.
823. Sozzi, G., et al., *Reply to comments on: Unexpected detection of SARS-CoV-2 antibodies in the prepandemic period in Italy*. Tumori, 2021. **107**(5): p. 472-473.
824. Guo, L., et al., *Reply to Yamaoka, et al.* Clinical Infectious Diseases, 2021. **72**(7): p. 1293.
825. Paul, S.S. and G. Biswas, *Repurposed antiviral drugs for the treatment of COVID-19: Syntheses, mechanism of infection and clinical trials*. Mini-Reviews in Medicinal Chemistry, 2021. **21**(9): p. 1123-1143.
826. Kumar, S., D. Pandey, and A. Kumar, *REPURPOSING OF POLIO VACCINE IN PREVENTION OF COVID-19: THINKING TOWARDS MORE OPTIONS*. Asia Pacific Journal of Health Management, 2022. **17**(1).
827. Sharma, D., *Repurposing of the childhood vaccines: could we train the immune system against the SARS-CoV-2.* Expert Review of Vaccines, 2021. **20**(9): p. 1051-1057.
828. Schirmer, P., et al., *Respiratory co-infections with COVID-19 in the Veterans Health Administration, 2020*. Diagnostic Microbiology and Infectious Disease, 2021. **100**(1).
829. Mehta, P., et al., *Respiratory Co-Infections: Modulators of SARS-CoV-2 Patients' Clinical Sub-Phenotype*. Frontiers in Microbiology, 2021. **12**.
830. Perezbusta-Lara, N., R. Tirado-Mendoza, and J.R. Ambrosio-Hernández, *Respiratory infections and coinfections: geographical and population patterns*. Gac Med Mex, 2020. **156**(4): p. 265-272.
831. Sonawane, A.A., J. Shastri, and S.B. Bavdekar, *Respiratory Pathogens in Infants Diagnosed with Acute Lower Respiratory Tract Infection in a Tertiary Care Hospital of Western India Using Multiplex Real Time PCR*. Indian J Pediatr, 2019. **86**(5): p. 433-438.
832. Volling, C., et al., *Respiratory syncytial virus infection-associated hospitalization in adults: a retrospective cohort study*. BMC Infect Dis, 2014. **14**: p. 665.
833. Saravanos, G.L., et al., *Respiratory syncytial virus subtype circulation and associated disease severity at an Australian paediatric referral hospital, 2014-2018*. J Paediatr Child Health, 2021. **57**(8): p. 1190-1195.
834. Rodríguez-Martínez, C.E., D.A. Rodríguez, and G. Nino, *Respiratory syncytial virus, adenoviruses, and mixed acute lower respiratory infections in children in a developing country*. J Med Virol, 2015. **87**(5): p. 774-81.
835. Yoshida, L.M., et al., *Respiratory syncytial virus: co-infection and paediatric lower respiratory tract infections*. Eur Respir J, 2013. **42**(2): p. 461-9.
836. Kim, K.W., et al., *Respiratory viral co-infections among SARS-CoV-2 cases confirmed by virome capture sequencing*. Scientific Reports, 2021. **11**(1).
837. Krumbein, H., et al., *Respiratory viral co-infections in patients with COVID-19 and associated outcomes: A systematic review and meta-analysis*. Reviews in Medical Virology, 2022.
838. Utokaparch, S., et al., *Respiratory viral detection and small airway inflammation in lung tissue of patients with stable, mild COPD*. Copd, 2014. **11**(2): p. 197-203.
839. Horn, M.E.C., E.A. Brain, and I. Gregg, *Respiratory viral infection and wheezy bronchitis in childhood*. Thorax, 1979. **34**(1): p. 23-28.
840. Ljungström, L.R., et al., *Respiratory viral infections are underdiagnosed in patients with suspected sepsis*. Eur J Clin Microbiol Infect Dis, 2017. **36**(10): p. 1767-1776.
841. Gaytán-Morales, J.F., et al., *Respiratory viral infections in pediatric patients with hematopoietic stem cell transplantation*. Bol Med Hosp Infant Mex, 2021. **78**(3): p. 191-199.
842. Sapra, M., et al., *Respiratory viral infections other than SARS CoV-2 among the North Indian patients presenting with acute respiratory illness during the first COVID-19 wave*. VirusDisease, 2022. **33**(1): p. 57-64.
843. Kanji, J.N., et al., *Respiratory virus co-infections with SARS-CoV-2 continue to be rare one year into the pandemic in Alberta, Canada (June 2020 - May 2021)*. Infection Control and Hospital Epidemiology, 2021.
844. Ching, N.S., et al., *Respiratory virus detection and co-infection in children and adults in a large Australian hospital in 2009-2015*. J Paediatr Child Health, 2018. **54**(12): p. 1321-1328.
845. To, K.K.W., et al., *Respiratory virus infection among hospitalized adult patients with or without clinically apparent respiratory infection: a prospective cohort study*. Clin Microbiol Infect, 2019. **25**(12): p. 1539-1545.
846. Zhan, Y., et al., *Respiratory virus is a real pathogen in immunocompetent community-acquired pneumonia: comparing to influenza like illness and volunteer controls*. BMC Pulm Med, 2014. **14**: p. 144.
847. Dia, N., et al., *Respiratory viruses associated with patients older than 50 years presenting with ILI in Senegal, 2009 to 2011*. BMC Infect Dis, 2014. **14**: p. 189.
848. Ali, A., et al., *Respiratory viruses associated with severe pneumonia in children under 2 years old in a rural community in Pakistan*. J Med Virol, 2016. **88**(11): p. 1882-90.
849. Wong-Chew, R.M., et al., *Respiratory viruses detected in Mexican children younger than 5 years old with community-acquired pneumonia: a national multicenter study*. Int J Infect Dis, 2017. **62**: p. 32-38.
850. Rhedin, S., et al., *Respiratory viruses in hospitalized children with influenza-like illness during the h1n1 2009 pandemic in Sweden [corrected]*. PLoS One, 2012. **7**(12): p. e51491.
851. Chen, H., et al., *Response of memory CD8+ T cells to severe acute respiratory syndrome (SARS) coronavirus in recovered SARS patients and healthy individuals*. J Immunol, 2005. **175**(1): p. 591-8.
852. Mathian, A., et al., *Response to: Â € Presence of anti-phospholipid antibodies in COVID-19: A case series study' by Amezcua-Guerra et al.* Annals of the Rheumatic Diseases, 2021. **80**(5).

853. Pérez-López, A., et al., *Resurgence of influenza A infections in children after the relaxation of COVID-19-related social distancing measures and normalization of international travel in Qatar.* Journal of Travel Medicine, 2022.
854. Ujiie, M., et al., *Resurgence of respiratory syncytial virus infections during COVID-19 pandemic, Tokyo, Japan.* Emerging Infectious Diseases, 2021. **27**(11): p. 2969.
855. Lakhan, N., et al., *Retrospective review of factors associated with severe hospitalised community-acquired influenza in a tertiary paediatric hospital in South Australia.* Influenza Other Respir Viruses, 2016. **10**(6): p. 479-485.
856. Rutvisuttinunt, W., et al., *Retrospective use of next-generation sequencing reveals the presence of Enteroviruses in acute influenza-like illness respiratory samples collected in South/South-East Asia during 2010-2013.* J Clin Virol, 2017. **94**: p. 91-99.
857. Chan, H.C., et al., *Return of other respiratory viruses despite the disappearance of influenza during COVID-19 control measures in Singapore.* J Clin Virol, 2021. **144**: p. 104992.
858. Messacar, K., et al., *Rhino/enteroviruses in hospitalized children: a comparison to influenza viruses.* J Clin Virol, 2013. **56**(1): p. 41-5.
859. Solomon, M.D., et al., *Risk of severe COVID-19 infection among adults with prior exposure to children.* Proceedings of the National Academy of Sciences of the United States of America, 2022. **119**(33).
860. Renk, H., et al., *Robust and durable serological response following pediatric SARS-CoV-2 infection.* Nature Communications, 2022. **13**(1).
861. Moga, E., E. Lynton-Pons, and P. Domingo, *The Robustness of Cellular Immunity Determines the Fate of SARS-CoV-2 Infection.* Frontiers in Immunology, 2022. **13**.
862. Smuts, H., L. Workman, and H.J. Zar, *Role of human metapneumovirus, human coronavirus NL63 and human bocavirus in infants and young children with acute wheezing.* J Med Virol, 2008. **80**(5): p. 906-12.
863. Al Mosawi, A.M.T., H.M. Kadhim, and H.M. Hameed, *The role of influenza vaccination in the COVID-19 infection: Impact on incidence and severity in Iraq.* Journal of Applied Pharmaceutical Science, 2022. **12**(8): p. 130-136.
864. Cong, B., et al., *The role of respiratory co-infection with influenza or respiratory syncytial virus in the clinical severity of COVID-19 patients: A systematic review and meta-analysis.* J Glob Health, 2022. **12**: p. 05040.
865. Gregg, I., *The role of viral infection in asthma and bronchitis.* Scandinavian Journal of Respiratory Diseases, 1975. **56**(93 sup.): p. 14.
866. Amat, F., et al., *RSV-hRV co-infection is a risk factor for recurrent bronchial obstruction and early sensitization 3 years after bronchiolitis.* J Med Virol, 2018. **90**(5): p. 867-872.
867. Getz, W.M., et al., *A runtime alterable epidemic model with genetic drift, waning immunity and vaccinations.* Journal of the Royal Society Interface, 2021. **18**(184).
868. Nguyen-Contant, P., et al., *S protein-reactive IGG and memory B cell production after human SARS-CoV-2 infection includes broad reactivity to the S2 subunit.* mBio, 2020. **11**(5): p. 1-11.
869. Logunov, D.Y., et al., *Safety and efficacy of the Russian COVID-19 vaccine: more information needed – Authors' reply.* The Lancet, 2020. **396**(10256): p. e54-e55.
870. Kaabi, N.A., et al., *Safety and immunogenicity of a hybrid-type vaccine booster in BBIBP-CorV recipients in a randomized phase 2 trial.* Nature Communications, 2022. **13**(1).
871. Li, J.X., et al., *Safety and immunogenicity of heterologous boost immunisation with an orally administered aerosolised Ad5-nCoV after two-dose priming with an inactivated SARS-CoV-2 vaccine in Chinese adults: a randomised, open-label, single-centre trial.* The Lancet Respiratory Medicine, 2022. **10**(8): p. 739-748.
872. Jin, P., et al., *Safety and immunogenicity of heterologous boost immunization with an adenovirus type-5-vectored and protein-subunit-based COVID-19 vaccine (Convidecia/ZF2001): A randomized, observer-blinded, placebo-controlled trial.* PLoS Medicine, 2022. **19**(5).
873. Seki, Y., et al., *Safety and immunogenicity of the Pfizer/BioNTech SARS-CoV-2 mRNA third booster vaccine dose against the BA.1 and BA.2 Omicron variants.* Med, 2022. **3**(6): p. 406-421.e4.
874. Picard, M., et al., *Safety of COVID-19 vaccination in patients with polyethylene glycol allergy: A case series.* Journal of Allergy and Clinical Immunology: In Practice, 2022. **10**(2): p. 620-625.e1.
875. Rojas-Pérez-ezquerra, P., et al., *Safety of new mrna vaccines against covid-19 in severely allergic patients.* Journal of Investigational Allergology and Clinical Immunology, 2021. **31**(2): p. 180-181.
876. Rigo, M.M., et al., *SARS-Arena: Sequence and Structure-Guided Selection of Conserved Peptides from SARS-related Coronaviruses for Novel Vaccine Development.* Frontiers in Immunology, 2022. **13**.
877. Munivenkatappa, A., et al., *SARS-CoV-2 & influenza A virus co-infection in an elderly patient with pneumonia.* Indian Journal of Medical Research, 2021. **153**(1): p. 190-195.
878. Van Tan, L., et al., *SARS-CoV-2 and co-infections detection in nasopharyngeal throat swabs of COVID-19 patients by metagenomics.* Journal of Cleaner Production, 2020.
879. Cuadrado-Payán, E., et al., *SARS-CoV-2 and influenza virus co-infection.* Lancet, 2020. **395**(10236): p. e84.
880. Aggarwal, N., et al., *SARS-CoV-2 and Influenza Virus Co-Infection Cases Identified through ILI/SARI Sentinel Surveillance: A Pan-India Report.* Viruses, 2022. **14**(3).
881. Orozco-Hernández, J.P., et al., *SARS-CoV-2 and rhinovirus/enterovirus co-infection in a critically ill young adult patient in Colombia.* Biomedica : revista del Instituto Nacional de Salud, 2020. **40**(2): p. 34-43.

882. Zervou, F.N., et al., *SARS-CoV-2 antibodies: IgA correlates with severity of disease in early COVID-19 infection*. Journal of Medical Virology, 2021. **93**(9): p. 5409-5415.
883. Onodera, T., et al., *A SARS-CoV-2 antibody broadly neutralizes SARS-related coronaviruses and variants by coordinated recognition of a virus-vulnerable site.* Immunity, 2021. **54**(10): p. 2385-2398.e10.
884. Phipps, W.S., et al., *SARS-CoV-2 Antibody Responses Do Not Predict COVID-19 Disease Severity.* Am J Clin Pathol, 2020. **154**(4): p. 459-465.
885. Sasaki, M., et al., *SARS-CoV-2 Bearing a Mutation at the S1/S2 Cleavage Site Exhibits Attenuated Virulence and Confers Protective Immunity.* mBio, 2021. **12**(4).
886. Swets, M.C., et al., *SARS-CoV-2 co-infection with influenza viruses, respiratory syncytial virus, or adenoviruses*. The Lancet, 2022. **399**(10334): p. 1463-1464.
887. Olvera, A., et al., *Sars-cov-2 consensus-sequence and matching overlapping peptides design for covid19 immune studies and vaccine development*. Vaccines, 2020. **8**(3): p. 1-14.
888. van der Heide, V., *SARS-CoV-2 cross-reactivity in healthy donors*. Nature Reviews Immunology, 2020. **20**(7): p. 408.
889. Musicò, A., et al., *SARS-CoV-2 epitope mapping on microarrays highlights strong immune-response to n protein region*. Vaccines, 2021. **9**(1): p. 1-11.
890. Carmen, J.M., et al., *SARS-CoV-2 ferritin nanoparticle vaccine induces robust innate immune activity driving polyfunctional spike-specific T cell responses*. npj Vaccines, 2021. **6**(1).
891. Grifoni, A., et al., *SARS-CoV-2 human T cell epitopes: Adaptive immune response against COVID-19*. Cell Host and Microbe, 2021. **29**(7): p. 1076-1092.
892. Karamloo, F. and R. König, *SARS-CoV-2 immunogenicity at the crossroads*. Allergy: European Journal of Allergy and Clinical Immunology, 2020. **75**(7): p. 1822-1824.
893. Nannu Shankar, S., et al., *SARS-CoV-2 in residential rooms of two self-isolating persons with COVID-19*. Journal of Aerosol Science, 2022. **159**.
894. Sun, D., et al., *SARS-CoV-2 infection in infants under 1 year of age in Wuhan City, China*. World J Pediatr, 2020. **16**(3): p. 260-266.
895. Guthmiller, J.J., et al., *SARS-CoV-2 infection severity is linked to superior humoral immunity against the spike*. mBio, 2021. **12**(1): p. 1-13.
896. Felsenstein, S. and C.M. Hedrich, *SARS-CoV-2 infections in children and young people*. Clinical Immunology, 2020. **220**.
897. Brodin, P., *SARS-CoV-2 infections in children: Understanding diverse outcomes*. Immunity, 2022. **55**(2): p. 201-209.
898. Mileto, D., et al., *SARS-CoV-2 mRNA vaccine BNT162b2 triggers a consistent cross-variant humoral and cellular response*. Emerging Microbes and Infections, 2021. **10**(1): p. 2235-2243.
899. Narowski, T.M., et al., *SARS-CoV-2 mRNA vaccine induces robust specific and cross-reactive IgG and unequal neutralizing antibodies in naive and previously infected people*. Cell Reports, 2022. **38**(5).
900. Woldemeskel, B.A., C.C. Garliss, and J.N. Blankson, *SARS-CoV-2 mRNA vaccines induce broad CD4+ T cell responses that recognize SARS-CoV-2 variants and HCoV-NL63*. Journal of Clinical Investigation, 2021. **131**(10).
901. Ahlén, G., et al., *The SARS-CoV-2 N protein is a good component in a vaccine*. Journal of Virology, 2020. **94**(18).
902. Elkoshi, Z., *SARS-CoV-2 Omicron (B.1.1.529) Variant: Corticosteroids Treatment/Respiratory Coinfection*. Frontiers in Immunology, 2022. **13**.
903. Richardson, S.I., et al., *SARS-CoV-2 Omicron triggers cross-reactive neutralization and Fc effector functions in previously vaccinated, but not unvaccinated, individuals*. Cell Host and Microbe, 2022. **30**(6): p. 880-886.e4.
904. Sałkowska, A., et al., *Sars-cov-2 proteins induce ifng in th1 lymphocytes generated from cd4+ cells from healthy, unexposed polish donors*. Vaccines, 2020. **8**(4): p. 1-10.
905. Starr, T.N., et al., *SARS-CoV-2 RBD antibodies that maximize breadth and resistance to escape*. Nature, 2021. **597**(7874): p. 97-102.
906. Singh, V., et al., *SARS-CoV-2 respiratory co-infections: Incidence of viral and bacterial co-pathogens*. International Journal of Infectious Diseases, 2021. **105**: p. 617-620.
907. Algaissi, A., et al., *SARS-CoV-2 S1 and N-based serological assays reveal rapid seroconversion and induction of specific antibody response in COVID-19 patients*. Sci Rep, 2020. **10**(1): p. 16561.
908. Ng, K.W., et al., *SARS-CoV-2 S2-targeted vaccination elicits broadly neutralizing antibodies*. Science translational medicine, 2022. **14**(655): p. eabn3715.
909. Choy, K.W., *SARS-CoV-2 serological cross-reactivity with autoantibodies*. The Lancet Rheumatology, 2021. **3**(1): p. e15.
910. Teng, J. and J. Dai, *SARS-CoV-2 serological cross-reactivity with autoantibodies – Authors' reply*. The Lancet Rheumatology, 2021. **3**(1): p. e16.
911. Cassaniti, I., et al., *SARS-CoV-2 specific T-cell immunity in COVID-19 convalescent patients and unexposed controls measured by ex vivo ELISpot assay*. Clin Microbiol Infect, 2021. **27**(7): p. 1029-1034.

912. Morris, R.S., *SARS-CoV-2 spike protein seropositivity from vaccination or infection does not cause sterility.* F and S Reports, 2021. **2**(3): p. 253-255.
913. Altmann, D.M. and R.J. Boyton, *SARS-CoV-2 T cell immunity: Specificity, function, durability, and role in protection.* Science Immunology, 2020. **5**(49).
914. Ameratunga, R., *SARS-CoV-2 the ASIA virus (Autoimmune/autoinflammatory Syndrome Induced by Adjuvants), the risk of infertility and vaccine hesitancy.* Expert Review of Vaccines, 2022.
915. Duan, L.J., et al., *SARS-CoV-2 vaccine-induced antibody and T cell response in SARS-CoV-1 survivors.* Cell Reports, 2022. **40**(9).
916. Mullbacher, A., J. Pardo, and Y. Furuya, *SARS-CoV-2 vaccines: Inactivation by gamma irradiation for T and B cell immunity.* Pathogens, 2020. **9**(11): p. 1-5.
917. Geers, D., et al., *SARS-CoV-2 variants of concern partially escape humoral but not T-cell responses in COVID-19 convalescent donors and vaccinees.* Science Immunology, 2021. **6**(59).
918. Danchin, A. and K. Timmis, *SARS-CoV-2 variants: Relevance for symptom granularity, epidemiology, immunity (herd, vaccines), virus origin and containment?* Environmental Microbiology, 2020. **22**(6): p. 2001-2006.
919. Oberemok, V.V., et al., *SARS-CoV-2 will constantly sweep its tracks: a vaccine containing CpG motifs in 'lasso' for the multi-faced virus.* Inflammation Research, 2020. **69**(9): p. 801-812.
920. Parrill, A., et al., *SARS-CoV-2-induced immunodysregulation and the need for higher clinical suspicion for co-infection and secondary infection in COVID-19 patients.* Journal of Microbiology, Immunology and Infection, 2021. **54**(1): p. 105-108.
921. Braun, J., et al., *SARS-CoV-2-reactive T cells in healthy donors and patients with COVID-19.* Nature, 2020. **587**(7833): p. 270-274.
922. Pia, L., *SARS-CoV-2-reactive T cells in patients and healthy donors.* Nature Reviews Immunology, 2020. **20**(6): p. 353.
923. Sharov, K.S., *SARS-CoV-2-related pneumonia cases in pneumonia picture in Russia in March-May 2020: Secondary bacterial pneumonia and viral co-infections.* Journal of global health, 2020. **10**(2): p. 020504.
924. Schmidt, K.G., et al., *SARS-CoV-2-Seronegative Subjects Target CTL Epitopes in the SARS-CoV-2 Nucleoprotein Cross-Reactive to Common Cold Coronaviruses.* Frontiers in Immunology, 2021. **12**.
925. Guo, L., et al., *SARS-CoV-2-specific antibody and T-cell responses 1 year after infection in people recovered from COVID-19: a longitudinal cohort study.* Lancet Microbe, 2022. **3**(5): p. e348-e356.
926. Le Bert, N., et al., *SARS-CoV-2-specific T cell immunity in cases of COVID-19 and SARS, and uninfected controls.* Nature, 2020. **584**(7821): p. 457-462.
927. Deng, Y., et al., *SARS-CoV-2-specific T cell immunity to structural proteins in inactivated COVID-19 vaccine recipients.* Cellular and Molecular Immunology, 2021. **18**(8): p. 2040-2041.
928. Rha, M.S., A. Reum Kim, and E.C. Shin, *SARS-CoV-2-specific T cell responses in patients with COVID-19 and unexposed individuals.* Immune Network, 2021. **21**(1): p. 1-11.
929. Bozkus, C.C., *SARS-CoV-2-specific T cells without antibodies.* Nature Reviews Immunology, 2020. **20**(8): p. 463.
930. de Vries, R.D., *SARS-CoV-2-specific T-cells in unexposed humans: presence of cross-reactive memory cells does not equal protective immunity.* Signal Transduction and Targeted Therapy, 2020. **5**(1).
931. Petrosillo, N., *SARS-CoV-2, "common cold" coronaviruses' cross-reactivity and "herd immunity": The razor of Ockham (1285-1347)?* Infectious Disease Reports, 2020. **12**(2).
932. Mohsen, M.O., et al., *A scalable and highly immunogenic virus-like particle-based vaccine against SARS-CoV-2.* Allergy: European Journal of Allergy and Clinical Immunology, 2022. **77**(1): p. 243-257.
933. Kleanthous, H., et al., *Scientific rationale for developing potent RBD-based vaccines targeting COVID-19.* npj Vaccines, 2021. **6**(1).
934. Rubin, R., *The Search for a Single Vaccine against Coronaviruses Yet to Come.* JAMA - Journal of the American Medical Association, 2021. **326**(2): p. 118-120.
935. Aguilar-Bretones, M., et al., *Seasonal coronavirus-specific B cells with limited SARS-CoV-2 cross-reactivity dominate the IgG response in severe COVID-19.* J Clin Invest, 2021. **131**(21).
936. Anderson, E.M., et al., *Seasonal human coronavirus antibodies are boosted upon SARS-CoV-2 infection but not associated with protection.* Cell, 2021. **184**(7): p. 1858-1864.e10.
937. Mateus, J., et al., *Selective and cross-reactive SARS-CoV-2 T cell epitopes in unexposed humans.* Science, 2020. **370**(6512).
938. Palladino, G., et al., *Self-amplifying mRNA SARS-CoV-2 vaccines raise cross-reactive immune response to variants and prevent infection in animal models.* Molecular Therapy - Methods and Clinical Development, 2022. **25**: p. 225-235.
939. Qiao, Y., et al., *A self-assembling nanoparticle vaccine targeting the conserved epitope of influenza virus hemagglutinin stem elicits a cross-protective immune response.* Nanoscale, 2022. **14**(8): p. 3250-3260.
940. Planas, D., et al., *Sensitivity of infectious SARS-CoV-2 B.1.1.7 and B.1.351 variants to neutralizing antibodies.* Nature Medicine, 2021. **27**(5): p. 917-924.
941. Comach, G., et al., *Sentinel surveillance of influenza-like illness in two hospitals in Maracay, Venezuela: 2006-2010.* PLoS One, 2012. **7**(9): p. e44511.

942. da Silva Ramos, F.J., F.G.R. de Freitas, and F.R. Machado, *Sepsis in patients hospitalized with coronavirus disease 2019: how often and how severe?* Current opinion in critical care, 2021. **27**(5): p. 474-479.
943. Song, S., et al., *Sequential immunization with SARS-CoV-2 RBD vaccine induces potent and broad neutralization against variants in mice*. Virology Journal, 2022. **19**(1).
944. Bao, L., et al., *Sequential infection with H1N1 and SARS-CoV-2 aggravated COVID-19 pathogenesis in a mammalian model, and co-vaccination as an effective method of prevention of COVID-19 and influenza*. Signal Transduction and Targeted Therapy, 2021. **6**(1).
945. Essaidi-Laziosi, M., et al., *Sequential infections with rhinovirus and influenza modulate the replicative capacity of SARS-CoV-2 in the upper respiratory tract.* Emerging Microbes and Infections, 2022. **11**(1): p. 412-423.
946. Clapham, H., et al., *Seroepidemiologic study designs for determining SARS-COV-2 transmission and immunity.* Emerging Infectious Diseases, 2020. **26**(9): p. 1978-1986.
947. Hicks, J., et al., *Serologic Cross-Reactivity of SARS-CoV-2 with Endemic and Seasonal Betacoronaviruses*. Journal of Clinical Immunology, 2021. **41**(5): p. 906-913.
948. Chia, W.N., et al., *Serological differentiation between COVID-19 and SARS infections*. Emerging Microbes and Infections, 2020. **9**(1): p. 1497-1505.
949. Trombetta, C.M., et al., *A serological investigation in Southern Italy: was SARS-CoV-2 circulating in late 2019?* Human Vaccines and Immunotherapeutics, 2022. **18**(5).
950. Chaudhury, S., et al., *Serological profiles of pan-coronavirus-specific responses in COVID-19 patients using a multiplexed electro-chemiluminescence-based testing platform*. PLoS ONE, 2021. **16**(6 June).
951. Peroni, L.A., et al., *Serological Testing for COVID-19, Immunological Surveillance, and Exploration of Protective Antibodies*. Frontiers in Immunology, 2021. **12**.
952. Tamminen, K., M. Salminen, and V. Blazevic, *Seroprevalence and SARS-CoV-2 cross-reactivity of endemic coronavirus OC43 and 229E antibodies in Finnish children and adults.* Clinical Immunology, 2021. **229**.
953. Alserehi, H.A., et al., *Seroprevalence of SARS-CoV-2 (COVID-19) among healthcare workers in Saudi Arabia: comparing case and control hospitals.* Diagn Microbiol Infect Dis, 2021. **99**(3): p. 115273.
954. Tseng, W.P., et al., *Seroprevalence Surveys for Anti-SARS-CoV-2 Antibody in Different Populations in Taiwan With Low Incidence of COVID-19 in 2020 and Severe Outbreaks of SARS in 2003*. Frontiers in Immunology, 2021. **12**.
955. Karunarathna, H., et al., *Serum anti-neuraminidase antibody responses in human influenza A(H1N1)pdm09 virus infections*. Emerg Microbes Infect, 2019. **8**(1): p. 404-412.
956. Ortega, N., et al., *Seven-month kinetics of SARS-CoV-2 antibodies and role of pre-existing antibodies to human coronaviruses.* Nature Communications, 2021. **12**(1).
957. Kim, H.K., et al., *Severe acute respiratory syndrome coronavirus 2 and influenza A virus co-infection alters viral tropism and haematological composition in Syrian hamsters*. Transboundary and Emerging Diseases, 2022.
958. Tomasik, A., et al., *Severe acute respiratory syndrome coronavirus 2 and influenza A virus co-infection in a nine-year-old boy from a family cluster - case report.* Pediatria Polska, 2020. **95**(3): p. 1-3.
959. Cooksey, G.L.S., et al., *Severe Acute Respiratory Syndrome Coronavirus 2 and Respiratory Virus Sentinel Surveillance, California, USA, May 10, 2020-June 12, 2021*. Emerging Infectious Diseases, 2022. **28**(1): p. 9-19.
960. Stapleton, J.T., *Severe Acute Respiratory Syndrome Coronavirus 2 Antibody Testing: Important but Imperfect.* Clinical Infectious Diseases, 2021. **73**(9): p. E3074-E3076.
961. Yang, F., et al., *Shared B cell memory to coronaviruses and other pathogens varies in human age groups and tissues*. Science, 2021. **372**(6543): p. 738-741.
962. Giannattasio, A., et al., *Silent RSV in infants with SARS-CoV-2 infection: A case series.* Pediatric Pulmonology, 2021.
963. Streng, A., et al., *Similar severity of influenza primary and re-infections in pre-school children requiring outpatient treatment due to febrile acute respiratory illness: prospective, multicentre surveillance study (2013-2015).* BMC Infect Dis, 2022. **22**(1): p. 12.
964. Jawalagatti, V., et al., *A Simplified SARS-CoV-2 Mouse Model Demonstrates Protection by an Oral Replicon-Based mRNA Vaccine*. Frontiers in Immunology, 2022. **13**.
965. Wishaupt, J.O., et al., *Single- and multiple viral respiratory infections in children: disease and management cannot be related to a specific pathogen.* BMC Infect Dis, 2017. **17**(1): p. 62.
966. Kramer, K.J., et al., *Single-cell profiling of the antigen-specific response to BNT162b2 SARS-CoV-2 RNA vaccine.* Nature Communications, 2022. **13**(1).
967. Gisondi, P., F. Bellinato, and G. Girolomoni, *Skin adverse reactions to Sars-CoV-2 vaccination: a relevant responsibility issue for dermatologists*. Journal of the European Academy of Dermatology and Venereology, 2022. **36**(2): p. 165-166.
968. Hu, W., et al., *Specific cd8+ tcr repertoire recognizing conserved antigens of sars-cov-2 in unexposed population: A prerequisite for broad-spectrum cd8+ t cell immunity*. Vaccines, 2021. **9**(10).
969. Choy, K.W., *Specificity and cross-reactivity of a test for anti-SARS-CoV-2 antibodies.* Lancet Infect Dis, 2021. **21**(5): p. e118.

970. Iversen, K., et al., *Specificity and cross-reactivity of a test for anti-SARS-CoV-2 antibodies – Authors' reply*. The Lancet Infectious Diseases, 2021. **21**(5): p. e119.
971. Kurz, H., et al., *Spectrum of pathogens of in-patient children and youths with community acquired pneumonia: a 3 year survey of a community hospital in Vienna, Austria*. Wien Klin Wochenschr, 2013. **125**(21-22): p. 674-9.
972. Ng, K.T., N.K. Mohd-Ismail, and Y.J. Tan, *Spike s2 subunit: The dark horse in the race for prophylactic and therapeutic interventions against sars-cov-2*. Vaccines, 2021. **9**(2): p. 1-12.
973. Stanojevic, M., et al., *Spike-directed vaccination elicits robust spike-specific T-cell response, including to mutant strains*. Cytotherapy, 2022. **24**(1): p. 10-15.
974. Zhang, J., et al., *Spike-specific circulating T follicular helper cell and cross-neutralizing antibody responses in COVID-19-convalescent individuals*. Nature Microbiology, 2021. **6**(1): p. 51-58.
975. Hutto, S.K., O. Rapalino, and N. Venna, *Spinomedullary Weston Hurst Syndrome After COVID-19 and Influenza Co-Infection: A Case Report*. Neurohospitalist, 2022. **12**(2): p. 337-340.
976. Shirley, K. and J.M. Loftis, *A spotlight on HCV and SARS-CoV-2 co-infection and brain function*. Pharmacology Biochemistry and Behavior, 2022. **217**.
977. Nasser, A. and F. Zakham, *A strategy for SARS-CoV-2 vaccination in Yemen*. The Lancet, 2021. **397**(10291): p. 2247.
978. Tauzin, A., et al., *Strong humoral immune responses against SARS-CoV-2 Spike after BNT162b2 mRNA vaccination with a 16-week interval between doses*. Cell Host and Microbe, 2022. **30**(1): p. 97-109.e5.
979. Agrati, C., et al., *Strong immunogenicity of heterologous prime-boost immunizations with the experimental vaccine GRAd-COV2 and BNT162b2 or ChAdOx1-nCOV19*. npj Vaccines, 2021. **6**(1): p. 131.
980. Finkelstein, M.T., et al., *Structural analysis of neutralizing epitopes of the sars-cov-2 spike to guide therapy and vaccine design strategies*. Viruses, 2021. **13**(1).
981. Gentile, Á., et al., *[Study of respiratory influenza A H1N1 Virus (pH1N1) in hospitalized children in the pandemic year. Experience in 34 centers in Argentina]*. Arch Argent Pediatr, 2011. **109**(3): p. 198-203.
982. Pourhassan, H., et al., *Successful outcome of pre-engraftment COVID-19 in an HCT patient: impact of targeted therapies and cellular immunity*. Blood Advances, 2022. **6**(6): p. 1645-1650.
983. Zhao, Q., et al., *Synthesis and immunological evaluation of synthetic peptide based anti-SARS-CoV-2 vaccine candidates*. Chemical Communications, 2021. **57**(12): p. 1474-1477.
984. Chiuppesi, F., et al., *Synthetic multiantigen MVA vaccine COH04S1 protects against SARS-CoV-2 in Syrian hamsters and non-human primates*. npj Vaccines, 2022. **7**(1).
985. Li, Y., et al., *Systematic evaluation of IgG responses to SARS-CoV-2 spike protein-derived peptides for monitoring COVID-19 patients*. Cellular and Molecular Immunology, 2021. **18**(3): p. 621-631.
986. Huang, A.T., et al., *A systematic review of antibody mediated immunity to coronaviruses: kinetics, correlates of protection, and association with severity*. Nature Communications, 2020. **11**(1).
987. Ogbe, A., et al., *T cell assays differentiate clinical and subclinical SARS-CoV-2 infections from cross-reactive antiviral responses*. Nature Communications, 2021. **12**(1).
988. Moss, P., *The T cell immune response against SARS-CoV-2*. Nature Immunology, 2022. **23**(2): p. 186-193.
989. Jordan, S.C., et al., *T cell immune responses to SARS-CoV-2 and variants of concern (Alpha and Delta) in infected and vaccinated individuals*. Cellular and Molecular Immunology, 2021. **18**(11): p. 2554-2556.
990. Niessl, J., T. Sekine, and M. Buggert, *T cell immunity to SARS-CoV-2*. Seminars in Immunology, 2021. **55**.
991. Jarjour, N.N., D. Masopust, and S.C. Jameson, *T Cell Memory: Understanding COVID-19*. Immunity, 2021. **54**(1): p. 14-18.
992. Shrotri, M., et al., *T cell response to SARS-cov-2 infection in humans: A systematic review*. PLoS ONE, 2021. **16**(1 January).
993. Nguyen, T.H.O., et al., *T Cells Targeting SARS-CoV-2: By Infection, Vaccination, and Against Future Variants*. Frontiers in Medicine, 2021. **8**.
994. Casado, J.L., et al., *T-cell response after first dose of BNT162b2 SARS-CoV-2 vaccine among healthcare workers with previous infection or cross-reactive immunity*. Clinical and Translational Immunology, 2021. **10**(9).
995. Nunes, M.C., et al., *T-cell responses to SARS-CoV-2 in unexposed South African women*. Gates Open Research, 2022. **5**.
996. Zeng, Q., et al., *Tackling COVID19 by Exploiting Pre-existing Cross-Reacting Spike-Specific Immunity*. Molecular Therapy, 2020. **28**(11): p. 2314-2315.
997. Grifoni, A., et al., *Targets of T Cell Responses to SARS-CoV-2 Coronavirus in Humans with COVID-19 Disease and Unexposed Individuals*. Cell, 2020. **181**(7): p. 1489-1501.e15.
998. Ghazvini, K., M. Karbalaei, and M. Keikha, *Third booster vaccination and stopping the Omicron, a new variant of concern*. Vacunas, 2022.
999. Kanakan, A., et al., *Threading the pieces together: Integrative perspective on sars-cov-2*. Pathogens, 2020. **9**(11): p. 1-32.
1000. Xie, T., et al., *Three doses of prototypic SARS-CoV-2 inactivated vaccine induce cross-protection against its variants of concern*. Signal Transduction and Targeted Therapy, 2022. **7**(1).

1001. Pujol, A., et al., *Thyroid as a target of adjuvant autoimmunity/inflammatory syndrome due to mRNA-based SARS-CoV2 vaccination: from Graves' disease to silent thyroiditis.* Journal of Endocrinological Investigation, 2022. **45**(4): p. 875-882.
1002. Peng, J.Y., et al., *Time-dependent viral interference between influenza virus and coronavirus in the infection of differentiated porcine airway epithelial cells.* Virulence, 2021. **12**(1): p. 1111-1121.
1003. Pereson, M.J., et al., *TNF-α Levels in Respiratory Samples Are Associated with SARSCoV- 2 Infection.* Microbiology Spectrum, 2022. **10**(1).
1004. Zhou, Q., et al., *To Be or Not To Be Vaccinated: That Is a Question in Myasthenia Gravis.* Frontiers in Immunology, 2021. **12**.
1005. Saurabh, S., et al., *Tobacco, alcohol use and other risk factors for developing symptomatic COVID-19 vs asymptomatic SARS-CoV-2 infection: a case-control study from western Rajasthan, India.* Trans R Soc Trop Med Hyg, 2021. **115**(7): p. 820-831.
1006. Ieven, T., et al., *Tolerability of polysorbate 80–containing COVID-19 vaccines in confirmed polyethylene glycol–allergic patients.* Journal of Allergy and Clinical Immunology: In Practice, 2021. **9**(12): p. 4470-4472.e1.
1007. Shi, M., et al., *Total infectome characterization of respiratory infections in pre-COVID-19 Wuhan, China.* PLoS Pathog, 2022. **18**(2): p. e1010259.
1008. Corona, A., et al., *Treating critically ill patients experiencing sars-cov-2 severe infection with ig-m and ig-a enriched ig-g infusion.* Antibiotics, 2021. **10**(8).
1009. González-Domínguez, I., et al., *Trivalent NDV-HXP-S Vaccine Protects against Phylogenetically Distant SARS-CoV-2 Variants of Concern in Mice.* Microbiology Spectrum, 2022. **10**(3).
1010. Sim, K.Y., et al., *Two Opposing Roles of SARS-CoV-2 RBD-Reactive Antibodies in Pre-Pandemic Plasma Samples From Elderly People in ACE2-Mediated Pseudovirus Infection.* Frontiers in Immunology, 2021. **12**.
1011. Canducci, F., et al., *Two-year prospective study of single infections and co-infections by respiratory syncytial virus and viruses identified recently in infants with acute respiratory disease.* J Med Virol, 2008. **80**(4): p. 716-23.
1012. Pawelec, G. and J. McElhaney, *Unanticipated efficacy of SARS-CoV-2 vaccination in older adults.* Immunity and Ageing, 2021. **18**(1).
1013. Okonji, E.F., et al., *Understanding varying COVID-19 mortality rates reported in Africa compared to Europe, Americas and Asia.* Tropical Medicine and International Health, 2021. **26**(7): p. 716-719.
1014. Rotulo, G.A., et al., *Unexpected peak of bronchiolitis requiring oxygen therapy in February 2020: Could an undetected SARS-CoV2-RSV co-infection be the cause?* Pediatric Pulmonology, 2021. **56**(6): p. 1803-1805.
1015. Appelberg, S., et al., *A universal SARS-CoV DNA vaccine inducing highly cross-reactive neutralizing antibodies and T cells.* EMBO Molecular Medicine, 2022.
1016. Niemeyer, B.F. and K.H. Benam, *Untapping host-targeting cross-protective efficacy of anticoagulants against SARS-CoV-2.* Pharmacology and Therapeutics, 2022. **233**.
1017. Thongpan, I., et al., *Upsurge of human rhinovirus infection followed by a delayed seasonal respiratory syncytial virus infection in Thai children during the coronavirus pandemic.* Influenza Other Respir Viruses, 2021. **15**(6): p. 711-720.
1018. El Kholy, A.A., et al., *The use of multiplex PCR for the diagnosis of viral severe acute respiratory infection in children: a high rate of co-detection during the winter season.* Eur J Clin Microbiol Infect Dis, 2016. **35**(10): p. 1607-13.
1019. Visseaux, B., et al., *Usefulness of multiplex PCR methods and respiratory viruses' distribution in children below 15 years old according to age, seasons and clinical units in France: A 3 years retrospective study.* PLoS One, 2017. **12**(2): p. e0172809.
1020. Karagöz, I.K., et al., *Using bioinformatic protein sequence similarity to investigate if SARS CoV-2 infection could cause an ocular autoimmune inflammatory reactions?* Experimental Eye Research, 2021. **203**.
1021. Warimwe, G.M., et al., *Using cross-species vaccination approaches to counter emerging infectious diseases.* Nature Reviews Immunology, 2021. **21**(12): p. 815-822.
1022. Alger, J., et al., *Using Prenatal Blood Samples to Evaluate COVID-19 Rapid Serologic Tests Specificity.* Matern Child Health J, 2020. **24**(9): p. 1099-1103.
1023. Price, O.H., et al., *Using routine testing data to understand circulation patterns of influenza A, respiratory syncytial virus and other respiratory viruses in Victoria, Australia.* Epidemiol Infect, 2019. **147**: p. e221.
1024. Sotgia, F. and M.P. Lisanti, *Using the common cold virus as a naturally occurring vaccine to prevent COVID-19: Lessons from Edward Jenner.* Aging, 2020. **12**(19): p. 18797-18803.
1025. Youssef, D., et al., *Vaccination against influenza among Lebanese health care workers in the era of coronavirus disease 2019.* BMC Public Health, 2022. **22**(1).
1026. Petersen, E. and P. Buchy, *Vaccination against SARS-CoV-2 should be included in childhood vaccination programs.* International Journal of Infectious Diseases, 2021. **106**: p. 429-430.
1027. Popowicz, G.M., K. Pyrc, and K. Hadian, *Vaccination versus SARS-CoV-2 Omicron: three vaccine doses win the battle.* Signal Transduction and Targeted Therapy, 2022. **7**(1).
1028. Blanas, A., et al., *Vaccination with a bacterial peptide conjugated to SARS-CoV-2 receptor-binding domain accelerates immunity and protects against COVID-19.* iScience, 2022. **25**(8).

1029. Gerges, D., et al., *Vaccination with BNT162b2 and ChAdOx1 nCoV-19 Induces Cross-Reactive Anti-RBD IgG against SARS-CoV-2 Variants including Omicron*. Viruses, 2022. **14**(6).
1030. Amanat, F., et al., *Vaccination with SARS-CoV-2 variants of concern protects mice from challenge with wild-type virus*. PLoS Biology, 2021. **19**(12).
1031. He, J., et al., *Vaccine design based on 16 epitopes of SARS-CoV-2 spike protein*. Journal of Medical Virology, 2021. **93**(4): p. 2115-2131.
1032. Ranzani, O.T. and F.A. Bozza, *Vaccine effectiveness of ChAdOx1 nCoV-19 against COVID-19 in a socially vulnerable community in Rio de Janeiro, Brazil: author's response*. Clinical Microbiology and Infection, 2022. **28**(8): p. 1166-1167.
1033. Anbarasu, A., S. Ramaiah, and P. Livingstone, *Vaccine repurposing approach for preventing COVID 19: can MMR vaccines reduce morbidity and mortality?* Human Vaccines and Immunotherapeutics, 2020. **16**(9): p. 2217-2218.
1034. Poland, G.A. and R.B. Kennedy, *Vaccine safety in an era of novel vaccines: a proposed research agenda*. Nature Reviews Immunology, 2022. **22**(4): p. 203-204.
1035. Kostoff, R.N., et al., *Vaccine- and natural infection-induced mechanisms that could modulate vaccine safety*. Toxicology Reports, 2020. **7**: p. 1448-1458.
1036. Gartlan, C., et al., *Vaccine-Associated Enhanced Disease and Pathogenic Human Coronaviruses*. Frontiers in Immunology, 2022. **13**.
1037. Zhou, R., et al., *Vaccine-breakthrough infection by the SARS-CoV-2 omicron variant elicits broadly cross-reactive immune responses*. Clinical and Translational Medicine, 2022. **12**(1).
1038. Park, T., et al., *Vaccines against SARS-CoV-2 variants and future pandemics*. Expert Review of Vaccines, 2022.
1039. Liu, J., et al., *Vaccines elicit highly conserved cellular immunity to SARS-CoV-2 Omicron*. Nature, 2022. **603**(7901): p. 493-496.
1040. Bauer, G., *The variability of the serological response to SARS-corona virus-2: Potential resolution of ambiguity through determination of avidity (functional affinity)*. Journal of Medical Virology, 2021. **93**(1): p. 311-322.
1041. Janahi, I., et al., *Viral aetiology of bronchiolitis in hospitalised children in Qatar*. BMC Infect Dis, 2017. **17**(1): p. 139.
1042. Bezerra, P.G., et al., *Viral and atypical bacterial detection in acute respiratory infection in children under five years*. PLoS One, 2011. **6**(4): p. e18928.
1043. Pigny, F., et al., *Viral co-infections among SARS-CoV-2-infected children and infected adult household contacts*. European Journal of Pediatrics, 2021. **180**(6): p. 1991-1995.
1044. Cebey-López, M., et al., *Viral Co-Infections in Pediatric Patients Hospitalized with Lower Tract Acute Respiratory Infections*. PLoS One, 2015. **10**(9): p. e0136526.
1045. Kriger, O., et al., *Viral co-pathogens in COVID-19 acute respiratory syndrome – what did we learn from the first year of pandemic?* International Journal of Infectious Diseases, 2022. **116**: p. 226-229.
1046. Spagnolello, O., et al., *Viral community acquired pneumonia at the emergency department: Report from the pre COVID-19 age*. J Med Virol, 2021. **93**(7): p. 4399-4404.
1047. Trovato, M., et al., *Viral Emerging Diseases: Challenges in Developing Vaccination Strategies*. Frontiers in Immunology, 2020. **11**.
1048. Feret, V., et al., *[Viral epidemiology and clinical severity during the peak of the influenza A(H1N1) variant epidemic in febrile respiratory diseases of children]*. Arch Pediatr, 2014. **21**(7): p. 709-15.
1049. Le Hingrat, Q., et al., *Viral epidemiology and SARS-CoV-2 co-infections with other respiratory viruses during the first COVID-19 wave in Paris, France*. Influenza and other Respiratory Viruses, 2021. **15**(4): p. 425-428.
1050. Kozinska, A., et al., *Viral Etiological Agent(s) of Respiratory Tract Infections in Symptomatic Individuals during the Second Wave of COVID-19 Pandemic: A Single Drive-Thru Mobile Collection Site Study*. Pathogens, 2022. **11**(4).
1051. Do, A.H., et al., *Viral etiologies of acute respiratory infections among hospitalized Vietnamese children in Ho Chi Minh City, 2004-2008*. PLoS One, 2011. **6**(3): p. e18176.
1052. Suryadevara, M., et al., *Viral etiology of acute febrile respiratory illnesses in hospitalized children younger than 24 months*. Clin Pediatr (Phila), 2011. **50**(6): p. 513-7.
1053. Becerra, M., et al., *[Viral etiology of severe acute respiratory infections in a pediatric intensive care unit]*. Rev Peru Med Exp Salud Publica, 2019. **36**(2): p. 231-238.
1054. Uwishema, O., et al., *Viral infections amidst COVID-19 in Africa: Implications and recommendations*. Journal of Medical Virology, 2021. **93**(12): p. 6798-6802.
1055. Sanchez-Codez, M.I., et al., *Viral Loads and Disease Severity in Children with Rhinovirus-Associated Illnesses*. Viruses, 2021. **13**(2).
1056. Martínez, P., et al., *[Viral respiratory co-infections in pediatric patients admitted for acute respiratory infection and their impact on clinical severity]*. Rev Chilena Infectol, 2012. **29**(2): p. 169-74.
1057. Bicer, S., et al., *Virological and clinical characterizations of respiratory infections in hospitalized children*. Ital J Pediatr, 2013. **39**: p. 22.

1058. Pillet, S., et al., *Virological diagnosis of lower respiratory tract infections.* Revue des Maladies Respiratoires, 2021. **38**(1): p. 58-73.
1059. Baay, M., et al., *Virology, epidemiology, immunology and vaccine development of SARS-CoV-2, update after nine months of pandemic.* Biologicals, 2021. **69**: p. 76-82.
1060. Berrajah, L.F., et al., *[Virus and Atypical Pathogens Detected in Community-Acquired Lower Respiratory Tract Infection in Infants and Children of Sfax Region, Tunisia].* Bull Soc Pathol Exot, 2018. **111**(2): p. 90-98.
1061. Kawaguchi, A., et al., *Virus detection in critically ill children with acute respiratory disease: a new profile in view of new technology.* Acta Paediatr, 2018. **107**(3): p. 504-510.
1062. Chen, N., et al., *Virus-host interaction networks as new antiviral drug targets for IAV and SARS-CoV-2.* Emerging Microbes and Infections, 2022. **11**(1): p. 1371-1389.
1063. Kono, J., et al., *Viruses associated with influenza-like-illnesses in Papua New Guinea, 2010.* J Med Virol, 2014. **86**(5): p. 899-904.
1064. Eissa, S., et al., *Voltammetric-based immunosensor for the detection of SARS-CoV-2 nucleocapsid antigen.* Microchimica Acta, 2021. **188**(6).
1065. Hamady, A., J.J. Lee, and Z.A. Loboda, *Waning antibody responses in COVID-19: what can we learn from the analysis of other coronaviruses?* Infection, 2022. **50**(1): p. 11-25.
1066. Housset, P., et al., *Waning but persistent humoral response 6 months after the third dose of the mRNA BNT162b2 vaccine in hemodialysis and peritoneal dialysis patients.* Journal of Nephrology, 2022. **35**(3): p. 783-785.
1067. Faleye, T.O.C., et al., *Wastewater-based epidemiology and long-read sequencing to identify enterovirus circulation in three municipalities in maricopa county, arizona, southwest united states between june and october 2020.* Viruses, 2021. **13**(9).
1068. Hellerstein, M., *What are the roles of antibodies versus a durable, high quality T-cell response in protective immunity against SARS-CoV-2?* Vaccine: X, 2020. **6**.
1069. Lalaoui, R., et al., *What could explain the late emergence of COVID-19 in Africa?* New Microbes and New Infections, 2020. **38**.
1070. Willyard, C., *What the Omicron wave is revealing about human immunity.* Nature, 2022. **602**(7895): p. 22-25.
1071. Palatnik-de-Sousa, C.B., *What Would Jenner and Pasteur Have Done About COVID-19 Coronavirus? The Urges of a Vaccinologist.* Frontiers in Immunology, 2020. **11**.
1072. Mahmood, M.M., et al., *What you need to know about children's COVID-19: A systematic review.* Chinese Journal of Contemporary Pediatrics, 2021. **23**(11): p. 1080-1090.
1073. Thaden, J.T. and S.A. Maskarinec, *When two for the price of one isn't a bargain: estimating prevalence and microbiology of bacterial co-infections in patients with COVID-19.* Clinical Microbiology and Infection, 2020. **26**(12): p. 1602-1603.
1074. Sinaei, R., et al., *Why COVID-19 is less frequent and severe in children: a narrative review.* World Journal of Pediatrics, 2021. **17**(1): p. 10-20.
1075. Sposato, B. and M. Scalese, *Why do children seem to be more protected against COVID-19? A hypothesis.* Medical Hypotheses, 2020. **143**.
1076. Zimmermann, P. and N. Curtis, *Why Does the Severity of COVID-19 Differ With Age? Understanding the Mechanisms Underlying the Age Gradient in Outcome Following SARS-CoV-2 Infection.* Pediatric Infectious Disease Journal, 2022. **41**(2): p. E36-E45.
1077. Le Bert, N., et al., *Widely heterogeneous humoral and cellular immunity after mild SARS-CoV-2 infection in a homogeneous population of healthy young men: Heterogenous immunity to SARS-CoV-2.* Emerging Microbes and Infections, 2021. **10**(1): p. 2141-2150.
1078. Kim, D.S., S. Rowland-Jones, and E. Gea-Mallorquí, *Will SARS-CoV-2 Infection Elicit Long-Lasting Protective or Sterilising Immunity? Implications for Vaccine Strategies (2020).* Frontiers in Immunology, 2020. **11**.
1079. Mancuso, M., S.E. Eikenberry, and A.B. Gumel, *Will vaccine-derived protective immunity curtail COVID-19 variants in the US?* Infectious Disease Modelling, 2021. **6**: p. 1110-1134.
1080. O'Hagan, D.T., et al., *"World in motion" – emulsion adjuvants rising to meet the pandemic challenges.* npj Vaccines, 2021. **6**(1).

References 179, 186, 195, 752 and 857 are cited in the main text.

[1-250][251-500][501-750][751-1000][1001-1080]
